# Reno-Protective Effect of Low Protein Diet Supplemented With α-Ketoacid Through Gut Microbiota and Fecal Metabolism in 5/6 Nephrectomized Mice

**DOI:** 10.3389/fnut.2022.889131

**Published:** 2022-06-30

**Authors:** Yifan Zhu, Haidong He, Yuyan Tang, Yinshun Peng, Ping Hu, Weiqian Sun, Ping Liu, Meiping Jin, Xudong Xu

**Affiliations:** ^1^Department of Nephrology, Minhang Hospital, Fudan University, Shanghai, China; ^2^Department of Nutrition and Food Hygiene, School of Public Health, Fudan University, Shanghai, China

**Keywords:** chronic kidney disease, low-protein diet supplemented with α-ketoacid, gut microbiota, fecal metabolism, renal fibrosis, 5/6Nx mice

## Abstract

**Background:**

Low protein supplemented with α-ketoacid diet (LKD) was recommended to be an essential intervention to delay the progression of chronic kidney disease (CKD) in patients who were not yet on dialysis. Aberrant gut microbiota and metabolism have been reported to be highly associated with CKD. However, the effect of LKD on gut microbiota and related fecal metabolism in CKD remains unclear.

**Methods:**

Mice were fed with normal protein diet (NPD group), low protein diet (LPD group), and low protein diet supplemented with α-ketoacid (LKD group) after 5/6 nephrectomy. At the end of the study, blood, kidney tissues, and feces were collected for biochemical analyses, histological, 16S rRNA sequence of gut microbiome, and untargeted fecal metabolomic analyses.

**Results:**

Both LKD and LPD alleviate renal failure and fibrosis, and inflammatory statement in 5/6 nephrectomized mice, especially the LKD. In terms of gut microbiome, LKD significantly improved the dysbiosis induced by 5/6Nx, representing increased α-diversity and decreased F/B ratio. Compared with NPD, LKD significantly increased the abundance of *g_Parasutterella, s_Parabacteroides_sp_CT06, f_Erysipelotrichaceae, g_Akkermansia, g_Gordonibacter, g_Faecalitalea*, and *s_Mucispirillum_sp_69*, and decreased *s_Lachnospiraceae_bacterium_28-4* and *g_Lachnoclostridium*. Moreover, 5/6Nx and LKD significantly altered fecal metabolome. Then, multi-omics analysis revealed that specific metabolites involved in glycerophospholipid, purine, vitamin B6, sphingolipid, phenylalanine, tyrosine and tryptophan biosynthesis, and microbes associated with LKD were correlated with the amelioration of CKD.

**Conclusion:**

LKD had a better effect than LPD on delaying renal failure in 5/6 nephrectomy-induced CKD, which may be due to the regulation of affecting the gut microbiome and fecal metabolic profiles.

## Introduction

Chronic kidney disease (CKD) affects nearly 850 million people worldwide, contributing to high incidences of morbidity and mortality (1). The studies on low protein diet (LPD) in delaying the progression of CKD have gradually deepened in both experimental models and clinical research (2–4). It has been recognized that restriction of dietary protein intake could reduce glomerular hyperfiltration and nitrogen waste products. However, given the potential side effects of LPD (such as insufficient amino acid supply and malnutrition), the implementation of restricting dietary protein intake has been vigorously debated. For these reasons, α-ketoacid, a nitrogen-free substitution for the essential amino acids, has been prescribed together with LPD to patients to delay CKD progressions (5). The studies currently available suggest that the effectiveness of LPD supplemented with α-ketoacid (LKD) was greater than LPD in reducing metabolic burden, protective role against oxidative stress of kidney tissue (6), and blood pressure control (7), while the mechanisms of LPD and LKD to slow down the progression of CKD are unclear.

In recent years, many studies have reported that gut dysbiosis altered host metabolome and impacted renal failure and complication of CKD both in humans and rodents (8–10). The composition and changes in the dietary structure have profound effects on the structure and metabolism of the gut microbiome. Although 5–10% of dietary amino acids reach the colon, where most gut microbiotas grow and metabolize, many diets-microbiome studies usually focused on the effects of dietary fiber, fat, and carbohydrates (11). Less is known about the specific effects of dietary protein on gut microbiota and metabolism in CKD. As LKD is a cornerstone of CKD treatment, the mechanistic roles of LKD-microbiota-metabolism interactions in CKD pathogenesis and treatment remain unclear.

Based on the above, we adopted different feeding methods for CKD mice induced by 5/6 subtotal nephrectomy to explore the effects of LKD and LPD on gut microbiota and fecal metabolism.

## Methods

### Experimental Animals and Study Design

**Figure 1:**
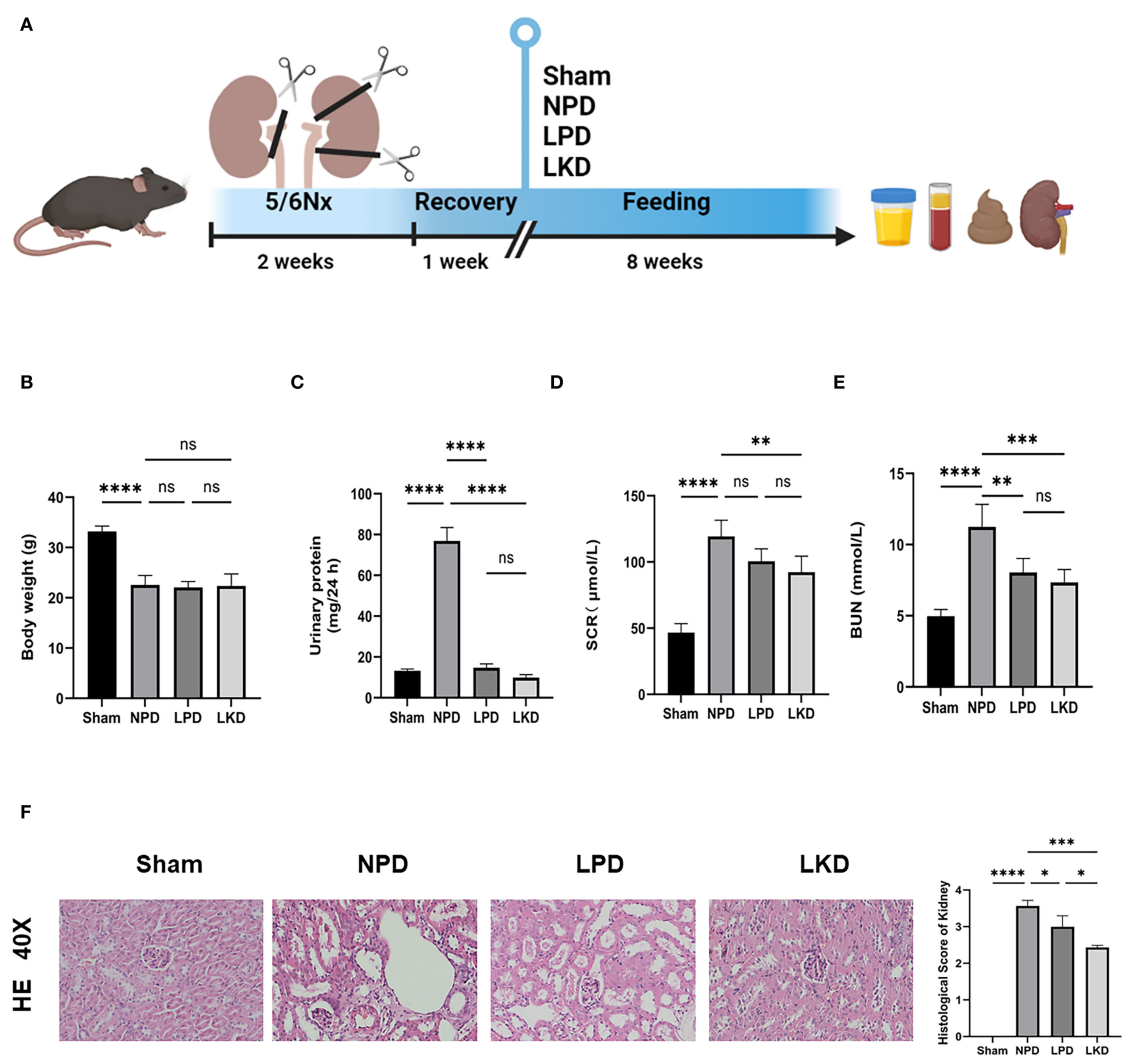
Flowchart, analysis of renal function, and kidney H&E staining. **(A)** The flowchart of the animal experiment. Mice were randomly divided into three groups after 5/6Nx and fed with different weight of protein. **(B)** Comparison of the body weight after 8-week feeding (ANOVA test). **(C)** Comparison of the urinary protein (24 h) (ANOVA test). **(D)** Comparison of the serum level of creatinine (ANOVA test). **(E)** Comparison of the level of blood urea nitrogen (ANOVA test). **(F)** Representative images (40×) for H&E staining of kidney tissues and score of renal injury (ANOVA test). Ns, not significant; *p < 0.05; **p < 0.01; ***p < 0.001; ****p < 0.0001.

SPF-level C57bl/6 wild-type mice (8 weeks old) were obtained from the Slack Company and raised at a standard environment (average temperature of 22°C with a standard 12 h/12 h light/dark cycle). After 1 week of adaptive feeding, we adopted the method of 5/6 subtotal nephrectomy (5/6Nx) to construct an animal model of CKD as follows: 1/3 of the upper and lower poles of the left kidney, a total of 2/3, was removed, and the entire right kidney was removed 7 days later (12). Sham operations (sham group, *n* = 5) were conducted at the same time points. After the CKD model was successfully constructed, the mice were randomly divided into three experimental groups (*n* = 6 per group), namely, (1) NPD group: CKD mice fed with a normal protein diet (protein accounts for 20% of the weight of feed); (2) LPD group: CKD mice fed with an LPD consisting of wheat starch (protein accounts for 5% of the weight); (3) LKD group: CKD mice fed with an LPD consisting of wheat starch (protein accounts for 5% of the weight of feed, 1% of which comes from α-ketoacid). In this study, only diets were given according to groups after modeling, without other intervention. The basic component of LPD and LKD diet is wheat starch. After 8 weeks of feeding, 24 h urine samples of mice were collected using the metabolic cages before they were euthanized under deep anesthesia. Plasma and feces samples were frozen and stored at −80°C for later analyses. Kidneys were collected for renal histological analyses. All experiments and operations in mice with CKD were performed in SPF-level animal breeding rooms. The flowchart of the treatment for mice is shown in Figure 1A.

### Biochemical and ELISA

Serum creatinine (SCR) and blood urea nitrogen (BUN) and 24 h urinary protein excretion were measured by commercial kits. Proinflammatory factors [interleukin 6 (IL-6), tumor necrosis factor alpha (TNF-α), and IL-1β] were analyzed by ELISA kits.

### Histological and Immunohistochemistry Analysis

Kidneys were fixed in 4% paraformaldehyde for 48 h and embedded in paraffin. Hematoxylin and eosin (H&E) staining was performed to evaluate the renal pathological injury. The renal injury score was estimated by morphometric assessment of tubular damage and interstitial fibrosis, which was analyzed by a blinded renal pathologist quantified staining in 10 randomly selected fields. Masson and Sirius red staining, and immunohistochemistry of smooth muscle alpha (α-SMA) antibody were performed to access the degree of renal fibrosis. The quantification of data was analyzed using the Image Pro Plus software.

### 16S rRNA Gene Sequencing

At week 12, the fresh feces of the mouse were shipped on dry ice and stored at −80°C until DNA extraction. DNA from samples was extracted using hexadecyltrimethylammonium bromide/sodium dodecyl sulfate (CTAB/SDS) method. DNA concentration and purity were monitored on 1% agarose gels and then diluted to l μg/μl using sterile water.

The 16S rRNA of V3–V4 regions were amplified using primer: 338F (5′-ACTCCTACGGGAGGCAGCAG-3′) and 806R (5′-GGACTACHVGGGTWTCTAAT-3′) with the barcode. All PCR reactions were carried out using 15 μl of Phusion^®^ High-Fidelity PCR Master Mix (New England Biolabs). The PCR products were mixed in equidensity ratios and purified using QIAGEN Gel Extraction Kit (QIAGEN, Germany).

Sequencing libraries were generated using TruSeq^®^ DNA PCR-Free Sample Preparation Kit (Illumina, USA). The library was sequenced on an Illumina NovaSeq platform to generate 250 bp paired-end reads.

Paired-end reads were assigned to samples and merged using FLASH (VI.2.7) (13). Quality filtering of the raw tags was performed to obtain the high-quality clean tag on QIIME (V1.9.1) (14). The tags were compared with the Silva database using the UCHIME algorithm (15) to remove the chimera sequences (16). Sequences with more than 97% similarity were assigned to the same operational taxonomic units (OTUs) using UPARSE (V 7.0.1001). The Silva Database (17) was used to annotate taxonomic information for each representative sequence based on the Mothur algorithm. The α-diversity and β-diversity were calculated using the QIIME software (V 1.9.1). Cluster analysis was preceded by non-metric multi-dimensional scaling (NMDS) analysis, which was a non-linear model analyzed based on the Bray-Curtis distance using the vegan package in the R software.

### Untargeted Fecal Metabolomic Analysis

Untargeted metabolomic analyses were performed on the fecal samples *via* the liquid chromatography-tandem mass spectrometry (LC-MS/MS). The UHPLC system (Vanquish, Thermo Fisher Scientific) with UPLC BEH Amide column (2.1 mm × 100 mm, 1.7 μm) were coupled to Q Exactive HFX mass spectrometer (Orbitrap MS, Thermo). The metabolite extraction, LC-MS/MS analysis, data preprocessing, and annotation are elaborated in the Supplementary Materials. The data were analyzed for positive and negative ions, respectively.

Metaboanalyst (https://www.metaboanalyst.ca) was implemented for the data cleaning, statistical analysis, and pathway enrichment analysis. The peak intensity matrix with a zero value in more than 50% of samples was filtered by removing peaks. The remaining missing values were replaced by one-fifth of the minimum positive value of each variable. Deviating values are filtered if their relative standard deviation is >25% and normalized using the mean value. Orthogonal projections to latent structures discriminant analysis (OPLS-DA) algorithm, fold change (fc), and *t*-tests were adopted to identify the metabolites with significant differences between groups. The permutation test (100 permutations) was performed to validate the OPLS-DA model. Differential expressed metabolites (DEMs) were identified by strict criteria, namely, variable importance in the projection (VIP) value > 1, log_2_ (fc) > |2|, *p* < 0.05. The Kyoto Encyclopedia of Genes and Genomes (KEGG) pathways of DEMs were enriched by Metaboanalyst and presented as potential targets with a threshold of *p* < 0.05.

### Statistics Analysis

All data with normal distributions are presented as mean ± SD of the mean, and statistical tests were performed using the GraphPad Prism software (V 9.1.2). Continuous normally distributed data among three groups were analyzed by one-way ANOVA. Kruskal-Wallis *H*-test was employed to analyze continuous data with non-normal distribution among three groups. The unpaired Student's *t*-tests were used to compare two groups and Mann-Whitney *U*-test was used for nonparametric data. Differential OTUs were identified using the DEseq2 package with a threshold of *p* < 0.05, log_2_(fc) > |1|. Spearman's correlation analysis was conducted to reveal the potential relationships between key DEMs and OTUs or parameters associated with CKD. Statistics notes: ns, not significant; ^*^p < 0.05; ^**^p < 0.01; ^***^p < 0.001. Figures were visualized using the R software.

## Results

### LKD Alleviated Renal Failure in 5/6Nx Mice

To verify the therapeutic effect of LPD and LKD, we first evaluate the bodyweight of mice after 8 weeks of different feeding. We found that NPD represented an obvious loss of weight compared with sham (22.6 ± 1.9 vs. 33.2 ± 1.1, *p* < 0.0001) (Figure 1B). However, LPD and LKD seem to not attenuate the loss of weight. Next, we evaluated the effects of different diets on the renal function of CKD mice. As shown in Figures 1C–E, the level of SCR (117.9 ± 13.9), BUN (11.2 ± 1.6), and 24 h urine protein (76.9 ± 6.6) in NPD was significantly higher than results in sham (46.7 ± 6.7; 5.0 ± 0.5; 13.2 ± 0.9), confirming our CKD model succeeded (all *p* < 0.0001). Compared with NPD, SCR level in LKD (91.2 ± 10.4, *p* = 0.007) was significantly decreased but not altered in LPD (100.6 ± 9.4, *p* = 0.093). Compared with NPD, BUN levels were both significantly decreased in LPD (8.0 ± 1.0, *p* = 0.002) and LKD (7.3 ± 0.9, *p* = 0.0002).

Moreover, the results of renal pathological changes were consistent with that of renal function changes. The structures of glomeruli and renal tubules in the kidney tissue of the sham mice were normal. In the CKD groups, there were different degrees of renal interstitial fibrosis, a large number of lymphocyte infiltration, renal tubular swelling, and necrosis. Compared with NPD, the degree of renal tissue damage was significantly improved, lymphocyte infiltration and renal tubular lesions were significantly reduced in the LPD (*p* = 0.015) and LKD (*p* = 0.0002) groups, especially in the LKD group (Figure 1F). Mice feeding LKD and LPD were protected against the effects of 5/6Nx, exhibiting significantly lower deterioration in both renal function and mathematical damage than mice feeding NPD.

### LKD Improved Renal Fibrosis and Inflammation in 5/6Nx Mice

**Figure 2:**
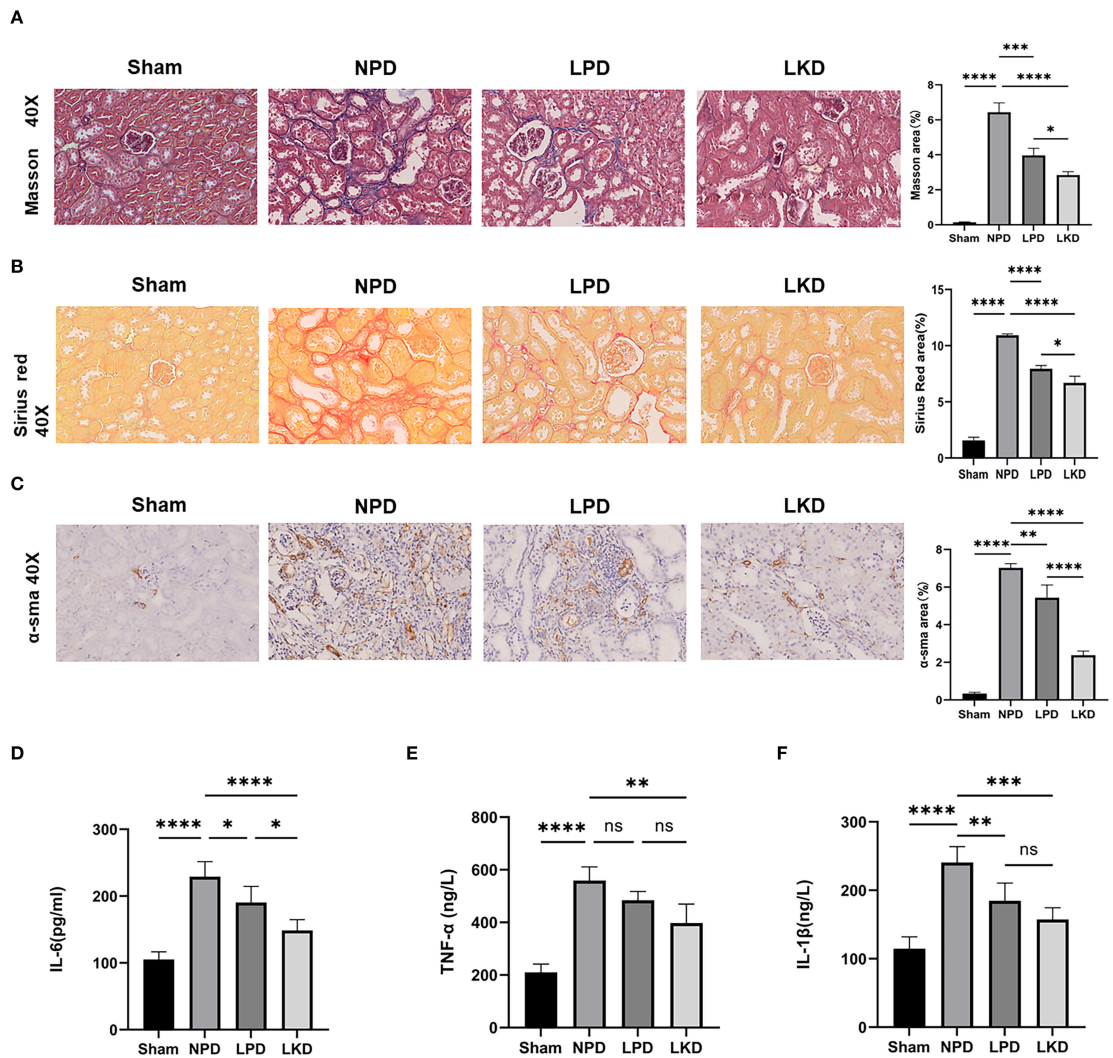
LKD attenuate renal fibrosis and decrease inflammatory factors. **(A)** Representative images (40×) for Masson staining of kidney tissues and fibrosis area score (ANOVA test). **(B)** Representative images (40×) for Sirius red staining of kidney tissues and fibrosis area score (ANOVA test). **(C)** Representative images (40×) for immunohistochemical α-SMA of kidney tissues and fibrosis area score (ANOVA test). **(D–F)** Comparison of inflammatory factors by ELISA. **(D)** IL-6, **(E)** TNF-α, **(F)** IL-1β (ANOVA test). Ns, not significant; *p < 0.05; **p < 0.01; ***p < 0.001; ****p < 0.0001.

To further verify whether LKD and LPD could delay the progression of renal failure induced by 5/6Nx, we performed Masson, Sirius red staining, and immunohistochemistry of α-SMA antibody to observe the degree of renal fibrosis in CKD mice. According to the Masson and Sirius red staining, no significant change in the glomerulus and metabolite deposition was observed in the renal tubule in the sham group. Only a small amount of collagen fibers is deposited in the renal interstitium-like cords in the sham group. In contrast, dilated glomerular balloon, thickened basement membrane, large collagen fibers, and deposition in renal tubule interstitium and epithelium were observed obviously in NPD compared with sham (Masson: *p* < 0.0001; Sirius red: *p* < 0.0001). Compared with NPD, LPD (Masson: *p* = 0.0001; Sirius red: *p* < 0.0001) and LKD (Masson: *p* < 0.0001; Sirius red: *p* < 0.0001) significantly improved the degree of fibrosis (Figures 2A,B). It is noteworthy that LKD showed better antifibrosis effect compared with LPD (Masson: *p* < 0.0001; Sirius red: *p* < 0.0001). Based on the results of immunohistochemistry, the expression of fibrosis markers α-SMA was significantly lower in LPD (*p* = 0.004) and LKD (*p* < 0.0001) compared with NPD (Figure 2C). The expression of α-SMA was significantly lower in LKD compared with LPD (*p* < 0.0001). In brief, the fibrosis degree of the LPD and LKD group was significantly reduced, especially the LKD group.

As CKD is often accompanied by a micro-inflammatory state, we detect three inflammatory factors (IL-6, TNF-α, and IL-1β) in serum to evaluate the level of inflammation (Figures 2D–F). NPD significantly increased IL-6 (229.2 ± 22.5), TNF-α (559.0 ± 51.8), and IL-1β (240.5 ± 23.4) compared with sham (105.0 ± 11.7; 210.4 ± 31.5; 210.4 ± 31.5; all *p* < 0.0001), indicating a micro-inflammatory state. LKD could significantly reduce the levels of IL-6 (148.5 ± 16.4, *p* < 0.0001), TNF-α (397.4 ± 72.3, *p* = 0.001), and IL-1β (157.3 ± 17.4, *p* = 0.0002) compared with NPD. LPD could significantly reduce the level of IL-1β (184.6 ± 26.1, *p*=0.007) and IL-6 (190.4 ± 24.2, *p*= 0.038) compared with NPD. Furthermore, the level of IL-6 in LKD was lower than that in LPD (*p* = 0.016), indicating a better anti-inflammatory ability of LKD.

### Effect of 5/6Nx and LKD on Gut Microbiome

**Figure 3:**
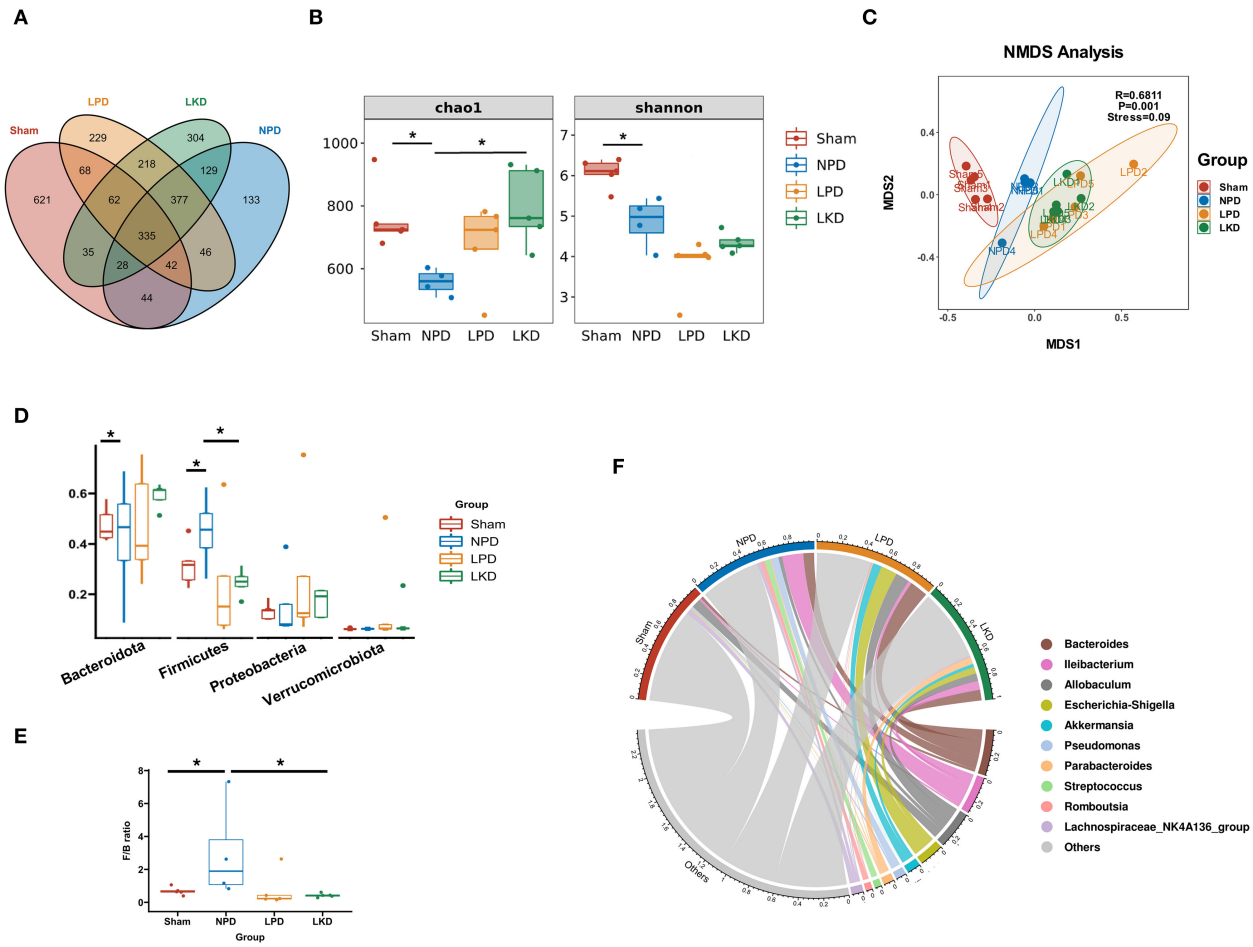
Gut microbiome taxonomic features of four groups. **(A)** Venn plot indicating the number of OTUs of four groups. **(B)** α-Diversity: Chao1 and Shannon indexes (Mann-Whitney *U*-test). **(C)** β-Diversity analyzed by NMDS based on Bray-Curtis distances. **(D)** Comparison of the relative abundance of gut microbiota at the phylum level (Mann-Whitney *U*-test). **(E)** Comparison of the F/B ratio (Mann-Whitney *U*-test). **(F)** Chord plot showing the top 10 genera and their contribution to each group. *P < 0.05.

In view of the influence of 5/6Nx and different diets on the gut microbiome, we explored the structure and differences in the gut microbiota of mice. A total of 2,671 OTUs were obtained after assigned and annotated. Venn plot (Figure 3A) showed that 335 OTUs coexisted in four groups and 621 OTUs unique to the sham group. While 377 were shared by three groups with 5/6Nx and 304 OTUs were unique in LKD. The α-diversity of the microbiome was estimated by two indexes, namely, the Chao1index reflected the community richness and the Shannon index reflected the community diversity. As shown in Figure 3B, Chao1 of NPD (558.1 ± 41.4) was significantly lower than that of sham (763.3 ± 105.4, *p* = 0.016) and LKD (796.5 ± 122.7, *p* = 0.016). Shannon of NPD was significantly lower than that of sham (4.9 ± 0.6 vs. 6.1 ± 0.4, *p* = 0.016). To further analyze the β-diversity of microbial composition, we performed NMDS based on Bray-Curtis distance (*R* = 0.6811, *p* = 0.001, stress = 0.09). The NMDS analysis (Figure 3C) visually revealed that sham was significantly separated from NPD, LPD, and LKD, which were farther apart. The clustering circles for LPD and LKD are closer and farther from sham and NPD. The NMDS results suggest that changing dietary patterns has a greater impact on the overall structure of the gut microbiota than 5/6Nx.

*Bacteroidetes, Firmicutes, Verrucomicrobia*, and *Proteobacteria* were the most dominant phylum in each group. Compared with the sham, *Firmicutes* population in NPD was increased (0.32 ± 0.09 vs.56 ± 0.12, *p* = 0.032) (Figure 3D), and the ratio of *Firmicutes* to *Bacteroidetes* (F/B) of NPD was significantly higher than that of sham (3.0 ± 3.0 vs. 0.68 ± 0.24, *p* = 0.032) (Figure 3E). In contrast, *Firmicutes* population in LKD was decreased compared with NPD (0.25 ± 0.05 vs. 0.56 ± 0.12, *p* = 0.016). *Bacteroidetes* population declined in the LKD group compared with NPD (0.59 ± 0.05 vs. 0.32 ± 0.19, *p* = 0.032) (Figure 3C). F/B ratio of LKD was lower than that of NPD (0.43 ± 0.12 vs. 3.0 ± 3.0, *p* = 0.016). For the genus level, *Bacteroides, Allobaculum, Ileibacterium, Escherichia-Shigella, Akkermansia, Pseudomonas, Parabacteroides, Streptococcus, Romboutsia*, and *Lachnospiraceae_NK4A136_group* were top 10 abundant genera. The chord diagram showed the distribution of these genera in each group (Figure 3F). Therefore, the four groups of mice differed in both the diversity and structure of the gut microbiome. The results suggested that LKD may improve the dysbiosis induced by 5/6Nx *via* increasing microbial richness and declining the F/B ratio.

### Effect of 5/6Nx on Fecal Metabolome

**Figure 4:**
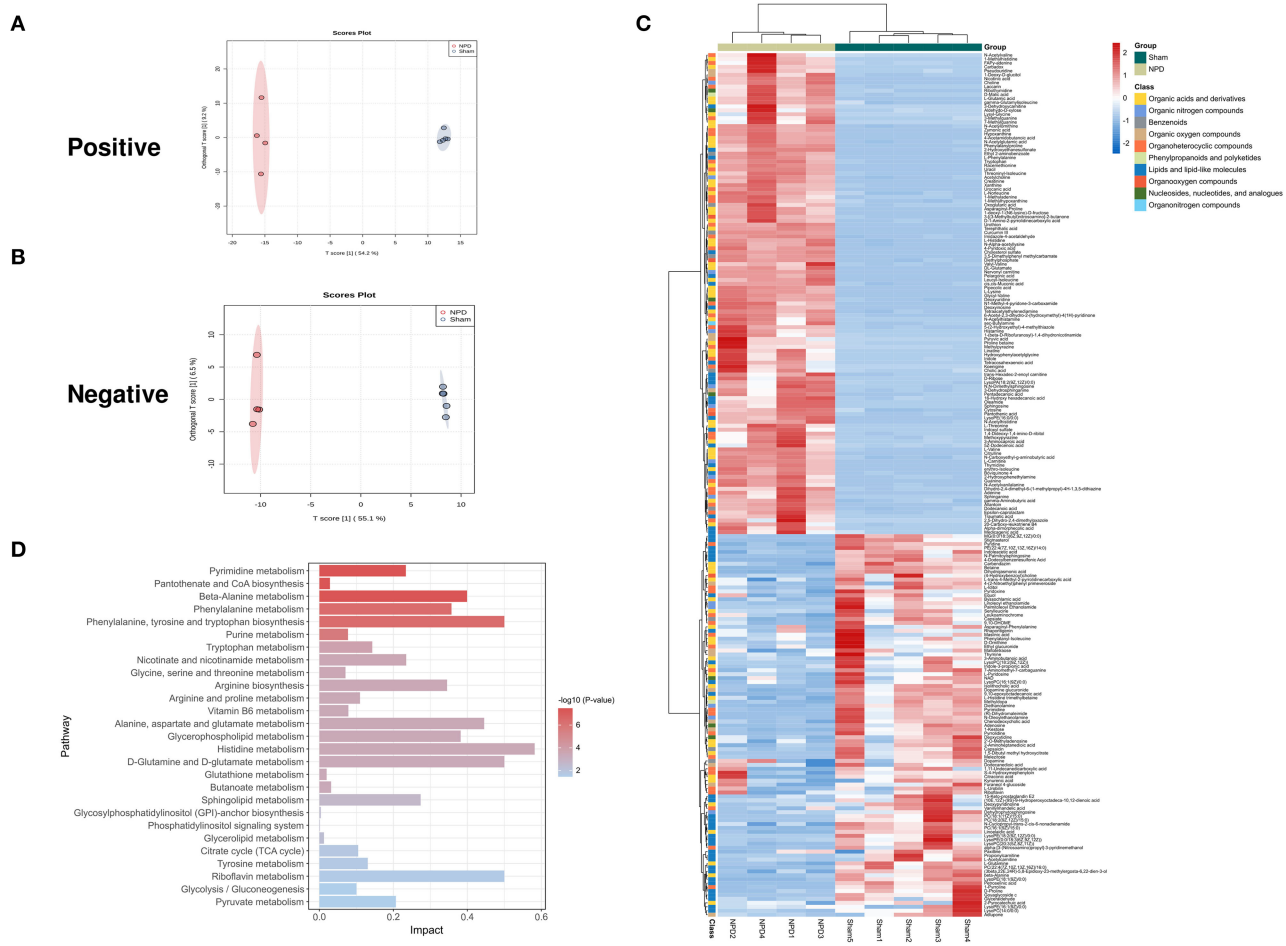
Different expressed metabolites between NPD and sham. **(A)** OPLS-DA score chart of positive mode between sham and NPD. **(B)** OPLS-DA score chart of negative mode between sham and NPD. **(C)** Heatmap of 219 DEMs between sham and NPD after combing positive and negative modes. The classification of these DEMs is indicated by different colors. **(D)** Enriched KEGG pathways of DEMs between sham and NPD with *p* < 0.05.

We used the UHPLC-QE-MS analytical technique to detect the changes in metabolites in the feces. Fecal metabolic profiling (OPLS-DA) showed that the sham and NPD groups can be clearly separated both in positive (Figure 4A) and negative modes (Figure 4B). Permutation test of OPLS-DA manifests that the model was reliable both in positive (Q2 = 0.979, *p* < 0.01; R2Y = 0.999, *p* < 0.01) and negative (Q2 = 0.972, *p* = 0.01; R2Y = 1, *p* = 0.01) modes. Under the strict criteria (VIP > 1, log_2_FC > |2|, *p* < 0.05) to identify the DEMs, 149 DEMs in positive mode and 80 DEMs in negative mode were eventually found between sham and NPD groups. The heatmap of 219 DEMs after merging the two modes suggested that 5/6Nx significantly reshaped the metabolic patterns of fecal metabolites (Figure 4C, Supplementary Table S1). DEMs between sham and NPD had obvious clustering in each group. In addition, these DEMs were mainly downregulated in NPD. These metabolic markers were mainly composed of organic acids and derivatives (30.1%), lipids and lipid-like molecules (24.2%), organoheterocyclic compounds (22.3%), organic nitrogen compounds (6.3%), organic oxygen compounds (6.3%), and others.

To explore the functional significance of these DEMs, metabolic pathway enrichment analysis was performed on the KEGG database. Metabolic pathways of 219 DEMs between sham and NPD were figured out, including metabolism of histidine; phenylalanine, tyrosine and tryptophan biosynthesis; d-glutamine and d-glutamate; riboflavin; alanine, aspartate and glutamate; beta-alanine; glycerophospholipid; phenylalanine; arginine biosynthesis; sphingolipid; nicotinate and nicotinamide; pyrimidine; pyruvate; tryptophan; tyrosine; arginine and proline; citrate cycle (TCA cycle); glycolysis/gluconeogenesis; vitamin B6; purine; glycine; serine and threonine; butanoate; pantothenate and CoA biosynthesis; glutathione; glycerolipid; glycosylphosphatidylinositol (GPI)-anchor biosynthesis; phosphatidylinositol signaling system (Figure 4D).

### Effect of LKD on Fecal Metabolome in 5/6Nx Mice

**Figure 5:**
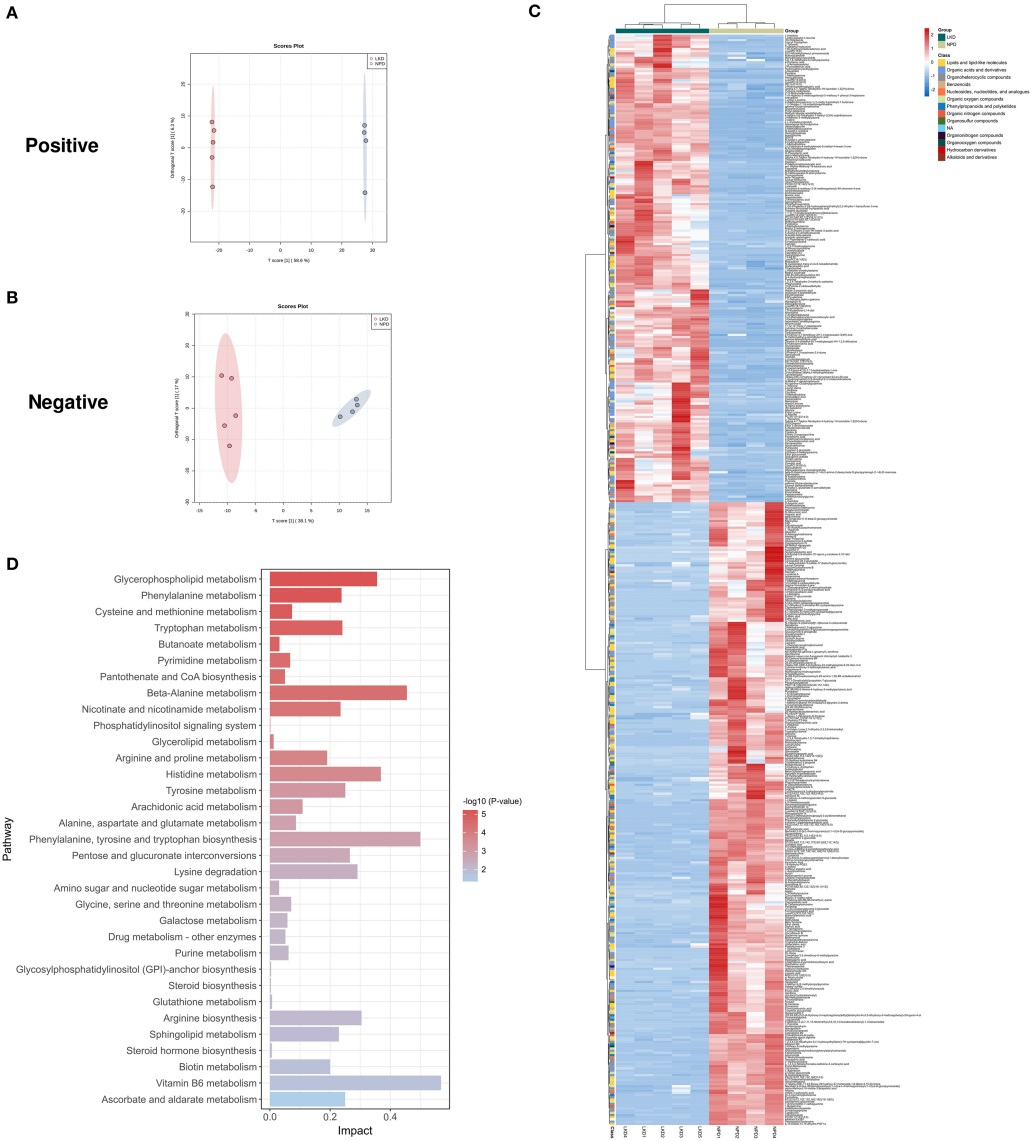
Different expressed metabolites between LKD and NPD. **(A)** OPLS-DA score chart of positive mode between LKD and NPD. **(B)** OPLS-DA score chart of negative mode between LKD and NPD. **(C)** Heatmap of 492 DEMs between LKD and NPD after combing positive and negative modes. The classification of these DEMs is indicated by different colors. **(D)** Enriched KEGG pathways of DEMs between LKD and NPD with *p* < 0.05.

As LKD had a better therapeutic effect on 5/6Nx than LPD, we further analyzed the differences in fecal metabolites between LKD, NPD, and LPD to explore its mechanism. The fecal metabolic profiles of LKD were different from those of NPD both in positive and negative modes (Figures 5A,B). The permutations test of OPLS-DA manifests that the model was reliable both in positive (Q2 = 0.99, *p* < 0.01; R2Y = 1, *p* < 0.01) and negative (Q2 = 0.925, *p* = 0.03; R2Y = 0.991, *p* = 0.04) modes. A total of 356 DEMs in positive mode and 67 DEMs in negative mode were eventually found between LKD and NPD. The heatmap of 492 DEMs after merging the two modes suggested that LKD pattern significantly reshaped the fecal metabolites compared with NPD (Figure 5C, Supplementary Table S2). DEMs between LKD and NPD had obvious clustering in each group. In addition, these DEMs were mainly downregulated in LKD. These DEMs were mainly composed of lipids and lipid-like molecules (21%), organic acids and derivatives (20%), organoheterocyclic compounds (20%), organic nitrogen compounds (8.7%), and others.

**Figure 6:**
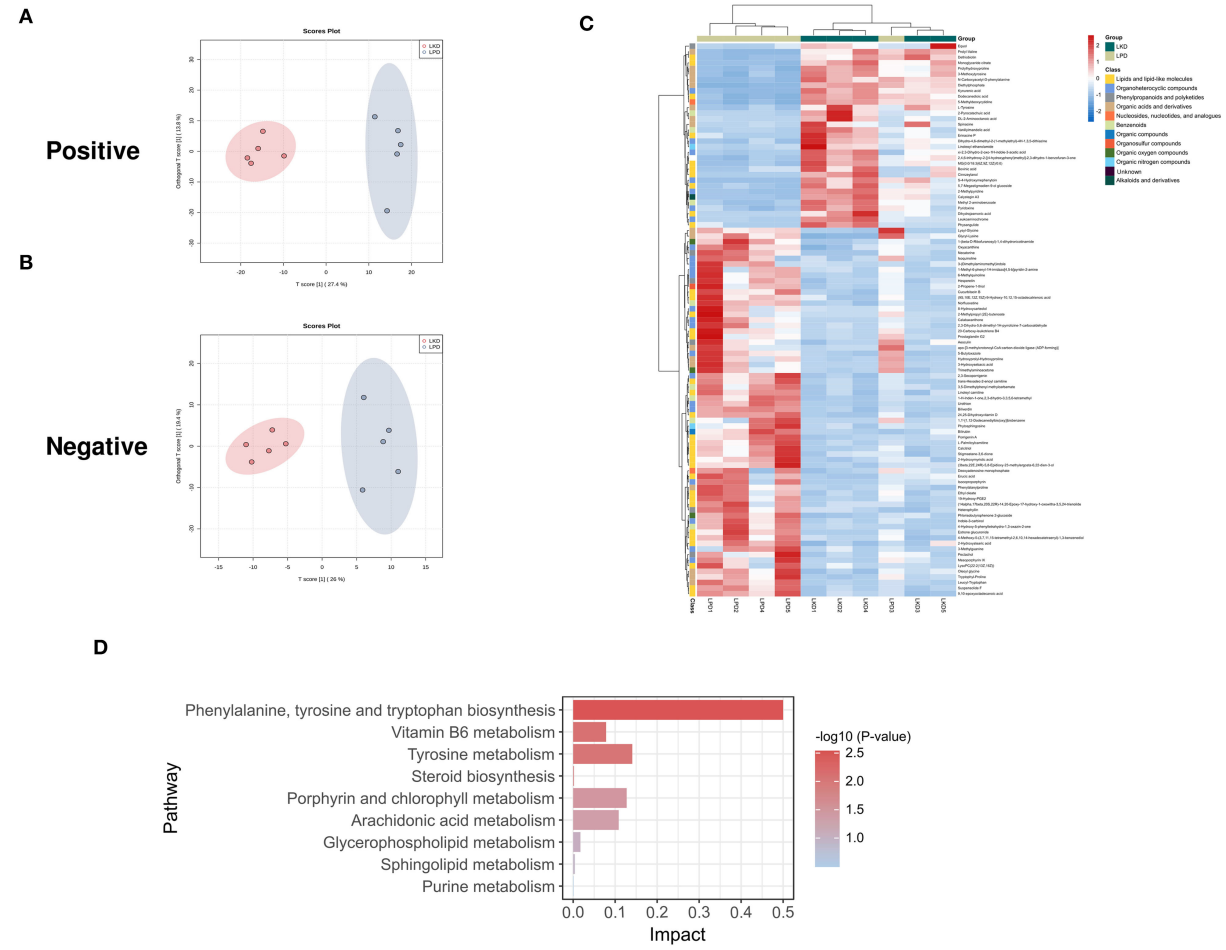
Different expressed metabolites between LKD and LPD. **(A)** OPLS-DA score chart of positive mode between sham and LPD. **(B)** OPLS-DA score chart of negative mode between LKD and LPD. **(C)** Heatmap of 99 DEMs between LKD and LPD after combing positive and negative modes. The classification of these DEMs is indicated by different colors. **(D)** Enriched KEGG pathways of DEMs between LKD and LPD with *p* < 0.05.

In terms of LKD vs. LPD, results of the permutation test of OPLS-DA (Figures 6A,B) model suggest that the difference between LKD and LPD was not as large as that between NPD both in positive (Q2 = 0.525, *p* = 0.07; R2Y = 0.971, *p* = 0.06) and negative (Q2 = 0.972, *p* = 0.01; R2Y = 1, *p* = 0.01) modes. Such results may be due to the relatively similar low protein dietary patterns of LKD and LPD. A total of 72 DEMs were identified in positive mode and 25 DEMs in negative mode were eventually found between LKD and NPD. The 99 DEMs between LKD and LPD after merging the two modes are shown in Figure 6C and Supplementary Table S3. DEMs between LKD and LPD showed not absolute clustering in each group, indicating the similar component of LKD and LPD. These DEMs were mainly composed of lipids and lipid-like molecules (34.3%), organoheterocyclic compounds (23.2%), organic acids and derivatives (17.1%), organic nitrogen compounds (8%), and others.

The KEGG pathway enrichment results (Figure 5D) showed metabolic pathways of 492 DEMs between LKD and NPD included metabolism pathways of phenylalanine, tyrosine and tryptophan biosynthesis; d-glutamine and d-glutamate; riboflavin; alanine, aspartate and glutamate; beta-alanine; glycerophospholipid; phenylalanine; arginine biosynthesis; sphingolipid; nicotinate and nicotinamide; pyrimidine; pyruvate; tryptophan; tyrosine; arginine and proline; citrate cycle (TCA cycle); glycolysis/gluconeogenesis; vitamin B6; purine; glycine, serine and threonine; butanoate; pantothenate and CoA biosynthesis; glutathione; glycerolipid; GPI-anchor biosynthesis; phosphatidylinositol signaling system. In addition, DEMs (LKD vs. LPD) were mainly enriched metabolism pathways of vitamin B6, glycerophospholipid, steroid hormone biosynthesis, etc. It can be seen that the differential metabolites between the LPD and NPD groups were less rather than other comparisons because the LKD can more significantly alter fecal metabolism in 5/6Nx mice.

The KEGG pathway enrichment results (Figure 6D) showed metabolic pathways of 99 DEMs between LKD and LPD included metabolism pathways of phenylalanine; tyrosine and tryptophan biosynthesis; tyrosine; porphyrin and chlorophyll; arachidonic acid; vitamin B6; glycerophospholipid; sphingolipid; steroid biosynthesis; and purine.

P-Cresyl sulfate (PCS), indoxyl sulfate (IS), and trimethylamine N-oxide (TMAO) were the products of the uremic toxins derived from gut microbiota. We analyzed the level of these toxins in feces. Compared with sham (7.6 ± 2.4), NPD (51.0 ± 16.5) significantly increased IS level in feces (*p* < 0.0001). While LKD (1.7 ± 1.6, *p* < 0.0001) and LPD (7.6 ± 4.5, *p* < 0.0001) could significantly decrease IS level. Although there was no statistical significance, PCS and TMAO in the NPD group showed an increasing trend compared with the sham group (Supplementary Figure S1).

### Identification of Key OTUs and Metabolites Associated With LKD

**Figure 7:**
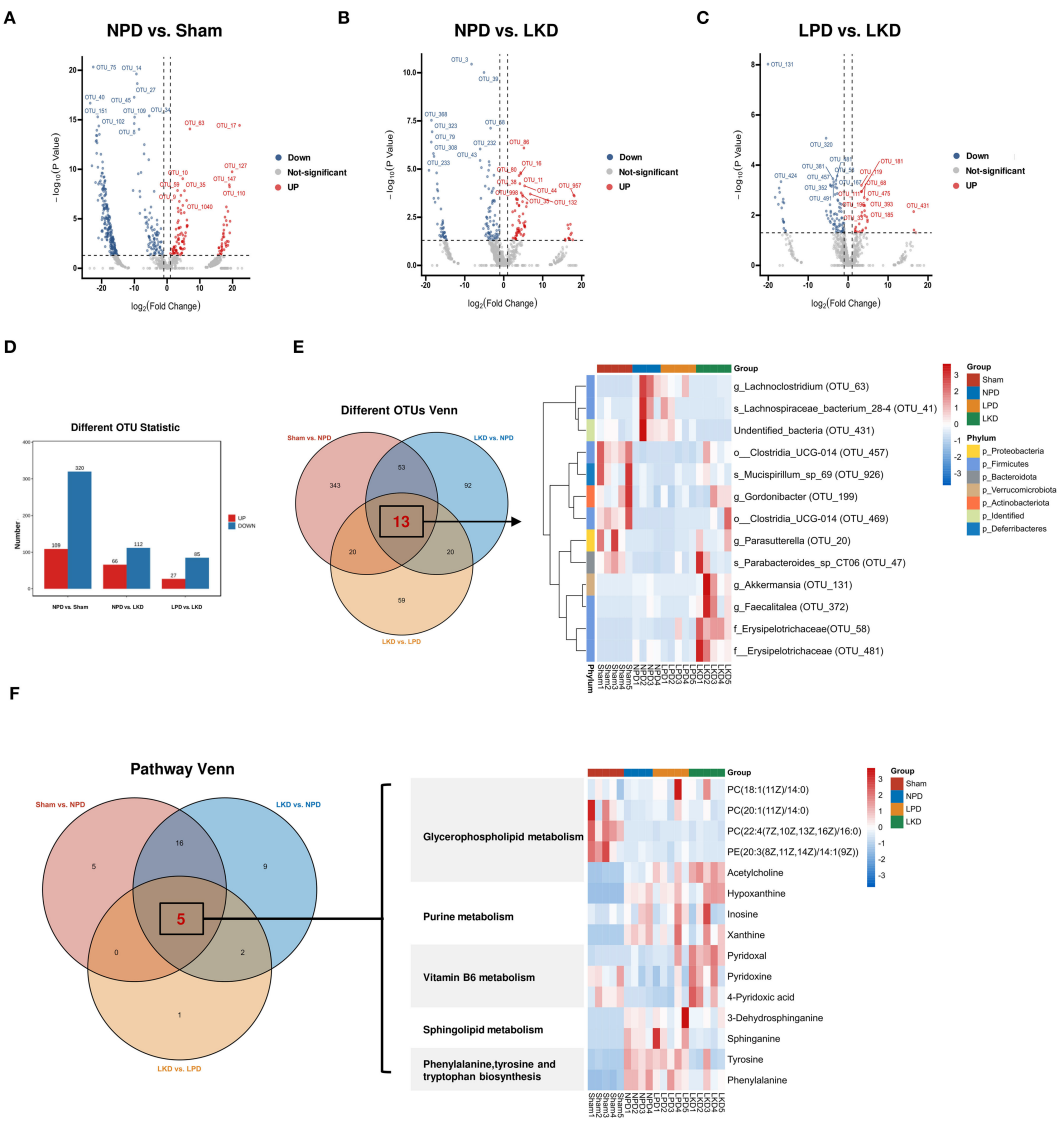
Identification of key DEMs and OTUs associated with LKD. **(A–C)** Volcano plots showing differential OTUs by DEseq2. **(A)** NPD vs. sham. **(B)** NPD vs. LKD. **(C)** LPD vs. LKD. **(D)** Histogram showing different numbers of OTUs among three comparisons. **(E)** Venn plot showing the overlapped differential OTUs among three comparisons; heatmap showing the abundance of overlapped differential OTUs. **(F)** Venn plot showing the overlapped differential KEGG metabolic pathways among three comparisons; heatmap showing the DEMs (NPD vs. LKD) involved in these pathways.

More than 50% of OTUs with zero abundance in at least one group were removed without further analysis (Supplementary Table S4). To further elucidate the alterations of the gut microbiome in LKD, DEseq2 was used and visualized in volcano plots with a threshold of *p* < 0.05, log_2_(fc) > |1| (Figures 7A–C). Figure 7D shows the numbers of differential abundant OTUs. A total of 429 OTUs showed significant differences between sham and NPD (Supplementary Table S5). A total of 320 OTUs depleted in NPD: OTU_75, OTU_14, OTU_27, OTU_45, OTU_40, etc., which mainly belong to *Lachnospiraceae_NK4A136_group* (9 OTUs), *Ruminococcus* (8 OTUs), *Bacteroides* (5 OTUs), *Corynebacterium* (5 OTUs), *Roseburia* (5 OTUs), *Enterorhabdus* (5 OTUs) at the genus level; 109 OTUs enriched in NPD: OTU_63, OTU_17, OTU_127, OTU_10, OTU_147, etc., which mainly belong to *Bacteroides* (4 OTUs)*, Lachnospiraceae_NK4A136_group* (4 OTUs), *Alistipes* (3 OTUs), *Clostridium_sensu_stricto_1* (2 OTUs), *Ileibacterium* (2 OTUs), *Parabacteroides* (2 OTUs), and *Faecalitalea* (2 OTUs) at the genus level.

A total of 178 OTUs showed significant differences between LKD and NPD (Supplementary Table S6). Of these, 112 OTUs enriched in LKD: OTU_3, OTU_39, OTU_368, OUT _68, OTU_323, etc., which mainly belong to *Corynebacterium* (6 OTUs), *Anaerococcus* (4 OTUs), *Monoglobus* (4 OTUs), *Peptoniphilus* (3 OTUs), *Escherichia-Shigella* (2 OTUs), and *Acinetobacter* (2 OTUs) at the genus level, and 66 OTUs depleted in LKD: OTU_86, OTU_16, OTU_80), OTU_38, OTU_11, etc., which mainly belong to *Lachnospiraceae_NK4A136_group* (6 OTUs), *Roseburia* (4 OTUs), and *Lachnoclostridium* (2 OTUs) at the genus level.

A total of 112 OTUs showed significant differences between LKD and LPD (Supplementary Table S7). Of these, 85 OTUs enriched in LKD: OTU_131, OTU_320, OTU_58, OTU_381, OTU_481, etc., which mainly belong to *Corynebacterium* (6 OTUs), *Anaerococcus* (4 OTUs), *Monoglobus* (4 OTUs), and *Peptoniphilus* (3 OTUs) at the genus level, and 27 OTUs depleted in LKD: OTU_119, OTU_181, OTU_111, OTU_68, OTU_475, etc., which mainly belong to *Blautia* (2 OTUs) and *Lachnospiraceae_NK4A136_group* (2 OTUs) at the genus level.

Venn diagram (Figure 7E) displaying the overlaps between comparisons showed that 13 OTUs were shared among the three comparisons (sham vs. NPD, LKD vs. NPD, LKD vs. LPD). These 13 OTUs can be regarded as LKD-specific differential OTUs that delayed 5/6Nx-induced renal failure and displaying in heatmap (Figure 7E). Compared with NPD, *g_Parasutterella* (OTU_20), *s_Parabacteroides*_sp_CT06 (OTU_47), *f_Erysipelotrichaceae* (OTU_58), *g_Akkermansia* (OTU_131), *g_Gordonibacter* (OTU_199), *g_Faecalitalea* (OTU_372), *o__Clostridia_UCG-014* (OTU_457), *o__Clostridia_UCG-014* (OTU_469), *f__Erysipelotrichaceae* (OTU_481), and *s_Mucispirillum_sp_69* (OTU_926) were enriched in LKD; *s_Lachnospiraceae_bacterium_28-4* (OTU_41), *g_Lachnoclostridium* (OTU_63), and *undentified_bacteria* (OTU_431) were depleted in LKD among the 13 LKD-specific OTUs. The OTUs annotated below the family level are identified as key OTUs.

According to the criteria of *p* < 0.05 and impact > 0, we screened out differential metabolic pathways. The Venn diagrams demonstrate the overlaps between comparisons showed that six pathways were shared among the three comparisons (sham vs. NPD, LKD vs. NPD, LKD vs. LPD). These common five pathways were identified as key pathways associated with LKD to delay 5/6Nx-induced renal failure, including phenylalanine, tyrosine and tryptophan biosynthesis; glycerophospholipid metabolism; sphingolipid metabolism; vitamin B6 metabolism; and purine metabolism. DEMs (LKD vs. NPD) involved in these key pathways were screened as key DEMs associated with LKD and displayed in the heatmap (Figure 7F).

### Multi-Omics Analysis Revealed the Association Between LKD, Gut Microbiome, and Fecal Metabolome

**Figure 8:**
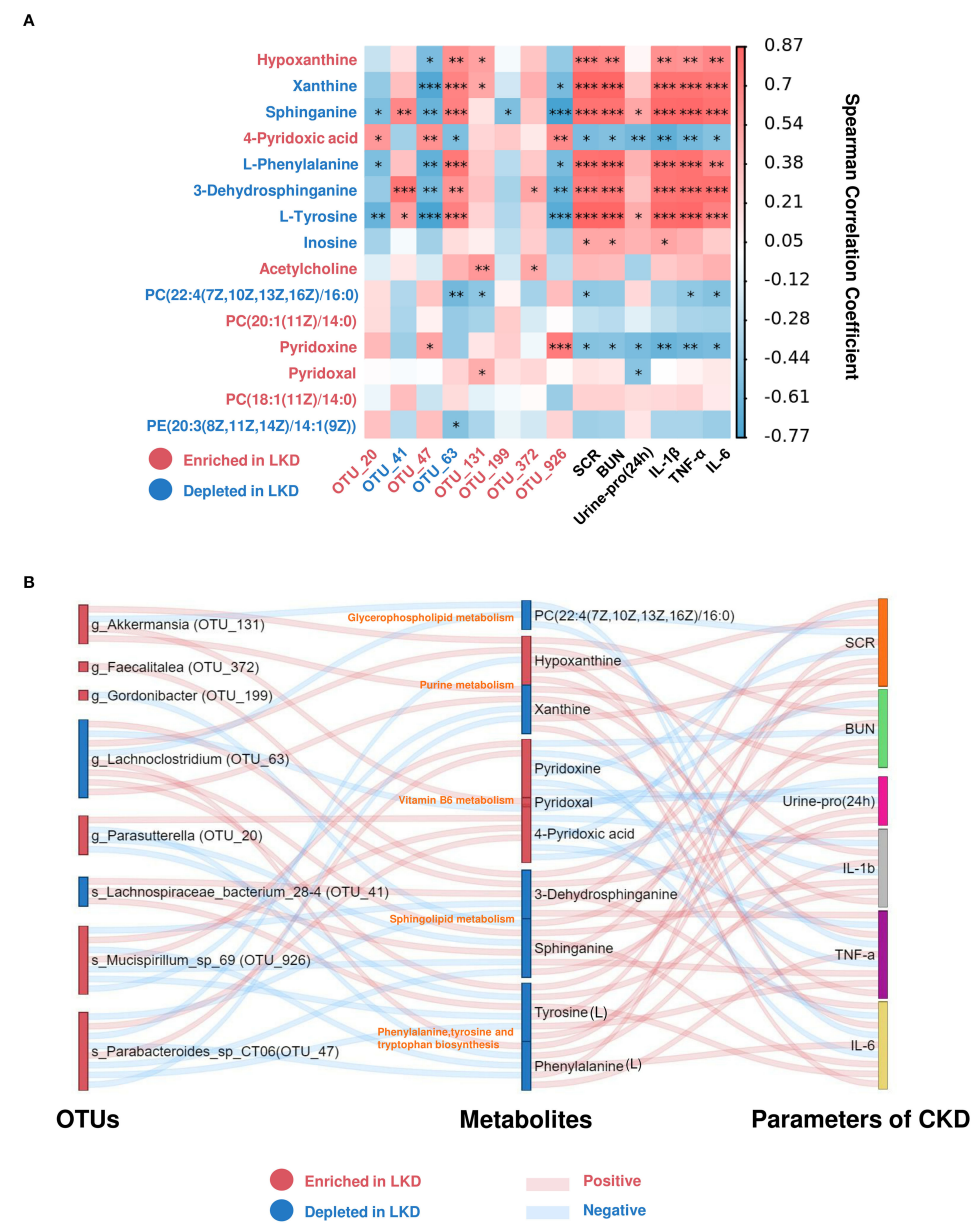
Spearman's correlations between differential metabolites and OTUs associated with LKD or parameters for CKD. **(A)** Heatmap showing Spearman's correlation between differential metabolites and OTUs associated with LKD or parameters for CKD. The IDs of metabolites or OTUs are highlighted in red (enriched in LKD) and blue (depleted in LKD). *p < 0.05, **p < 0.01, ***p < 0.001. **(B)** Sankey plot was used to visualize the relationship among the differential OTUs and serum metabolites associated with LKD, and major parameters of CKD. Red connections indicate significant positive correlations, and blue connections indicate significant negative correlations (Spearman's correlation analysis, *p* < 0.05). In the left and middle column, blue boxes indicate OTUs or DEMs that are significantly depleted in LKD compared with NPD, and red boxes indicate OTUs or DEMs that are significantly elevated in LKD compared with NPD.

To further explore the mechanism of LKD, we analyzed the association between key OTUs and fecal metabolites by using Spearman's correlation (Figure 8A). Several key OTUs were significantly associated with fecal metabolites, which suggests that the alterations in the gut microbiome induced by LKD may affect the fecal metabolites features. Furthermore, we correlated the key metabolites with parameters associated with CKD (renal function and inflammatory factors). Similarly, several significant Spearman's correlations were found between renal function parameters, inflammatory factors, and key metabolites. Key metabolites that were significantly correlated (*p* < 0.05) with both key OTUS and parameters were screened and displayed in Sankey diagram (Figure 8B). The r-value and *p*-value of Spearman's correlation are organized in Supplementary Table S8. We noted that *s_Parabacteroides_sp_CT06* (OTU_47, enriched in LKD), *s_Mucispirillum_sp_69* (OTU_926, enriched in LKD), *g_Akkermansia* (OTU_131, enriched in LKD), and *g_Lachnoclostridium* (OTU_63, depleted in LKD) were the four main biomarkers that impacted the fecal metabolome. PC(22:4(7Z,10Z,13Z,16Z)/16:0) (depleted in LKD and involved in glycerophospholipid metabolism) was negatively correlated with parameters associated with CKD. Xanthine (depleted in LKD) and hypoxanthine (enriched in LKD) involved in purine metabolism were positively correlated with CKD. Pyridoxine, pyridoxal, and 4-pyridoxic acid (enriched in LKD and involved in vitamin B6 metabolism) were negatively correlated with CKD. 3-Dehydrosphinganine and sphinganine (depleted in LKD and involved in sphingolipid metabolism) were positively correlated with CKD. l-Phenylalanine and l-tyrosine (depleted in LKD and involved in phenylalanine, tyrosine and tryptophan biosynthesis) were positively correlated with CKD.

## Discussion

In recent years, through an in-depth study of gut microbiota and metabolomics and the proposal of “kidney-gut axis” theory, more studies have proved that gut microbiome and its metabolites contribute to the progression of CKD. Different from traditional drugs and renal replacement therapy, targeting the regulation of gut microbiota and metabolism has gradually become an emerging hotspot in the treatment of CKD (18). It is well known that the gut microbiota is a key bridge between diet and host physiological metabolism; changes in diet can drive the composition and function of the gut microbiota to impact host metabolism (19). Most of the research on CKD focuses on the gut microbiome and the blood metabolism of the host. However, fecal metabolism can directly observe the metabolism of the gut microbiota. In this study, we constructed 5/6Nx mice model fed with different protein components of diet to investigate the mechanism of low protein diet with α-ketoacid in the treatment of CKD through gut microbiota and fecal metabolism. Our results showed that both LKD and NPD could present a protective effect against CKD by reducing urinary protein, SCR, BUN, and slowing the progression of renal failure and fibrosis in 5/6Nx-induced mice, especially LKD, which was consistent with those of previous studies (20).

The gut microbiota has a crucial effect on maintaining intestinal homeostasis both in health and the disease state (21). Previous studies reported that gut dysbiosis has been observed in CKD, representing lower richness and diversity, and changes in the abundance of phylum or other levels, compared with healthy controls (22). *Firmicutes* and *Bacteroidetes* accounted for more than 90% of the human gut microbiome. F/B ratio was also an important indicator reflecting the disorder of gut microbiota (23, 24). In this study, a lower richness and diversity were observed in 5/6Nx mice compared with sham group. LKD could improve community richness (Chao1index), but not improved the reduction of community diversity (Shannon index) induced by 5/6Nx. In addition, *Firmicutes* significantly increased in NPD, and the F/B ratio increased in NPD compared with sham. According to the richness recovered of and the decrease of the F/B ratio in LKD, we believe that LKD effectively alleviated the disorder of gut microbiota in 5/6Nx mice. Observing β-diversity *via* NMDS analysis, we found that the composition of gut microbiota in sham and NPD was quite different, and in LKD and NPD was either different. While the composition in LKD and LPD was more similar. Interestingly, there were more differential metabolites between sham and NPD, more differential metabolites between LKD and NPD, but fewer differential metabolites between LKD and LPD. The differential metabolites between LKD and NPD were more than between sham and NPD, indicating that changing dietary patterns may have more effects on fecal metabolism in mice than performing 5/6Nx.

Several studies have shown that gut dysbiosis promotes renal fibrosis and failure mainly by affecting host metabolism (10). Patients with end-stage renal disease (ESRD) tend to have an increase in the microbiota producing a variety of harmful metabolites, such as IS, PCS, and TMAO, and a decrease in the microbiota-producing beneficial metabolites, such as short-chain fatty acids (SCFAs) (10). These harmful bacteria affect the metabolism of the host, disrupt the intestinal barrier, promote the circulation of harmful metabolites through the damaged intestinal barrier, and then contribute to the progression of renal fibrosis and CKD through various mechanisms, such as promoting inflammation and oxidative stress in CKD (25). Applying DEseq2 algorithm to further explore the differences in gut microbiome, we found that OTUs between NPD and sham were significantly different. Depleted OTUs in NPD partly belong to genera that produce SCFAs, including *Bacteroides, Lachnospiraceae_NK4A136_group*, and *Roseburia* (26). It is widely believed that mainly SCFAs, such as propionate, acetate, and butyrate, are beneficial to human health by maintaining gastrointestinal health due to their ability to enhance epithelial barrier integrity and inhibit inflammation (27). What's more, metabolic pathway analysis manifested that butanoate metabolism was altered between NPD and sham. Therefore, we speculate that the changes in butanoate metabolism in NPD may be due to the changes in these bacteria. Unfortunately, LKD did not seem to increase SCFAs. No SCFA pathway was found in the differential metabolic pathway between LKD and NPD. The abundances of *g_Lachnospiraceae_NK4A136_group* and *g_Roseburia* in LKD were even lower than those in NPD. Therefore, we believe that LKD ameliorates CKD through other metabolic pathways rather than SCFAs.

We also focused on gut microbiota-derived metabolic toxicants associated with CKD, including IS, PCS, and TMAO (28). Although we did not find changes in PCS and TMAO, LKD reduced IS, which is consistent with some previous studies (29). LKD can reduce the precursors of PCS, namely, tyrosine and phenylalanine. However, Mo (30) reported that α-ketoacid alone increased tyrosine and phenylalanine in CKD rats. This indicates that the combined LPD diet will change the sole effect of α-ketoacid.

As LKD produced a better renal protective effect than LPD, we were more concerned about the effect of LPD combined with α-ketoacid, that is, LKD rather than LPD along. We performed further multi-omics association analysis by comparing the shared differential OTUs and metabolic pathways obtained between sham and NPD, between LKD and NPD, and between LKD and LPD as key OTUs and metabolic pathways associated with LKD. Compared with NPD, *s_Mucispirillum_sp_69* (OTU_926), *g_Parasutterella* (OTU_20), *s_Parabacteroides*_sp_CT06 (OTU_47), *g_Akkermansia* (OTU_131), *g_Gordonibacter* (OTU_199), *g_Faecalitalea* (OTU_372) were enriched in LKD; *g_Lachnoclostridium* (OTU_63), and *s_Lachnospiraceae_bacterium_28-4* (OTU_41) were depleted in LKD among the LKD-specific key OTUs, which identified at least genus level. Using the same approach, we screened out key metabolic pathways and key metabolites involved in them. Results suggested that LKD may ameliorate the progression of CKD by improving the metabolic disorder of phenylalanine, tyrosine and tryptophan biosynthesis; glycerophospholipid metabolism; sphingolipid metabolism; vitamin B6 metabolism; and purine metabolism.

*Parasutterella* and *Akkermansia* were reported to be enriched in healthy controls compared with patients with CKD (31). In addition, another research confirmed that LPD was able to significantly increase *Akkermansiaceae* and in line with our results (32). *Parasutterella* is a healthy core genus of the human and mouse gut microbiota. It occupies specific gut niches and affects the microbiota and host metabolism, which was defined recently (33). *Akkermansia muciniphila* is the only probiotics in human intestinal tract that can store and decompose mucin to generate carbon and nitrogen factors (34). Gaining effects of *A. muciniphila* have been reported in many diseases, such as diabetes, obesity, fatty liver, and tumors (35–37). There were no studies on the effects of *A. muciniphila* supplementation on CKD, and this needs more research. *Lachnoclostridium*, a novel fecal marker for the non-invasive diagnosis of colorectal adenoma and cancer (38), also enriched in many diseases such as obesity and polycystic ovary syndrome (39). In comparison to healthy controls, patients with CKD5 exhibited a significantly higher relative abundance of *Lachnoclostridium* and correlated with IS and PCS (40). The results of multi-omics association analysis were similar to those reported in literature; thus, we speculate that depleted *g_Lachnoclostridium* (OTU_63) in LKD may ameliorate CKD by decreasing l-phenylalanine and l-tyrosine, which were the precursors of PCS.

Using the same approach, we screened out key metabolic pathways associated with LKD and key metabolites involved in them. Results suggested that LKD may ameliorate the progression of CKD by improving the metabolic disorder of phenylalanine, tyrosine and tryptophan biosynthesis; glycerophospholipid metabolism; sphingolipid metabolism; vitamin B6 metabolism; and purine metabolism. Increasing studies indicated that serum lipid profile and lipid metabolism altered markedly during CKD pathogenesis. CKD leads to deep changes in lipid metabolism and obvious dyslipidemia. The dysregulation of lipid metabolism in turn results in CKD progression (41). The imbalance of serum sphingolipid metabolism (increasing deoxysphingolipids) was observed in both patients with CKD and 5/6Nx rat, and correlated with oxidative stress marker malondialdehyde (MDA) (42). However, the relationship of gut microbiota and sphingolipid in CKD is still unclear. Our multi-omics correlation analysis raised the hypothesis that LKD may improve CKD by reducing fecal sphinganine and 3-dehydrosphinganine through gut microbiota.

As is known to all, the disturbance in purine metabolism pathway and a higher level of serum uric acid (SUA), called hyperuricemia, has been linked to increased prevalence and progression of CKD (43). LPD combined with oral inulin can significantly reduce SUA in patients with CKD, with an increasing *Akkermansiaceae* and *Pasteurellaceae* mentioned above. In addition, the enhanced host purine metabolism in the germ-free mice promotes the conversion of the administered adenine into 2,8-DHA, resulting in exacerbated kidney damage (44). This suggests that the microbiota may play an important role in regulating purine metabolism. We found that LKD altered the purine metabolism pathway by decreasing xanthine and increasing hypoxanthine. Furthermore, xanthine produces uric acid by xanthine oxidase plasma, and xanthine oxidase activity is predictive of cardiovascular disease in CKD, independently of uric acid (45). Therefore, according to the multi-omics correlation analysis, we speculate that LKD may ameliorate CKD by altering gut microbiota, such as *g_Akkermansia* (OTU_131), thereby affecting purine metabolism.

Keto-analog administration can protect against ischemia-reperfusion induced renal injury and fibrosis by attenuating inflammatory infiltration and apoptosis (46), which is in line with our results. Oxidative stress and inflammation are involved in the occurrence and development of CKD. They interact with each other and play an important role in the pathogenesis of cardiovascular, neurological disease, and other complications in CKD (47). Vitamin B6, an acknowledged crucial antioxidant, is mainly obtained from diet and gut bacterial synthesis *via* intestinal absorption in the body, which cannot be synthesized by the human body itself (48). Our results found that LKD could significantly increase vitamin B6 metabolism and its metabolites in feces: pyridoxine, pyridoxal, and 4-pyridoxate. In addition, the multi-omics correlation showed that vitamin B6 metabolites in turn showed negative correlation with SCR, BUN, urine protein (24 h), and inflammatory factors, and correlated with several OTUs. Therefore, we speculate that LKD may function *via* gut microbiome to promote vitamin B6, ameliorating CKD and renal fibrosis.

It is noteworthy that pyridoxamine (PM) as an analog of vitamin B6 is in a phase 3 clinical efficacy trial to delay CKD progression in patients with diabetic kidney disease by interfering with oxidative macromolecular damage (49, 50). PM can also reduce postinjury fibrosis and renal oxidative damage, improving functional recovery after acute kidney injury (51). Oxidative stress and inflammation are quite common in CKD, and targeting them to treat CKD has become a research hotspot in recent years (52). Vitamin B6 (pyridoxine) deficiency often occurs in CKD, particularly those on dialysis (53). However, whether vitamin B6 supplementation can delay the progression of CKD through antioxidative stress and anti-inflammation is still unclear. There are few studies on LKD and vitamin B6. Mo's study (30) could only show that 4-pyridoxate was a differential serum metabolite between CKD rats treated with α-ketoacid and on a regular diet, and had a positive correlation with *Parasutterella*, which was also enriched in LKD in our study. Our results also showed that LKD could increase the fecal metabolite of vitamin B6, which is not observed in the LPD group, suggesting that this effect is dependent on α-ketoacid. The transamination of ketoacids is an important biological phenomenon, and studies have shown that vitamin B6 and transaminase play an important role in the process of transamination of α-ketoacids in biological systems to form optically active α-amino acids (54). A recent study on vitamin B6 and autism reported the relationship between the gut microbiota and vitamin B6. EPHB6-knockout mice have disordered vitamin B6 metabolic pathways in the pre-frontal cortex, showing decreased PM and pyridoxal 5'-phosphate (PLP, the main metabolically active form of vitamin B6), and a decreased abundance of *Mucispirillum* in gut (48). *Mucispiriinteracts* interacts closely with the intestinal epithelium in the terminal ileum (55), which is a core member of the murine gut microbiota. This study found that LKD diet can increase the abundance of *s_Mucispirillum_sp_69*, and it was significantly positively correlated with upregulated vitamin B6 in feces. Vitamin B6 is one of the important coenzymes that convert ketoacids into amino acids, thus LKD may delay CKD by promoting gut microbiota to synthesize vitamin B6. Therefore, it may be speculated that LKD promotes intestinal vitamin B6 metabolism by increasing *s_Mucispirillum_sp_69* to play anti-inflammatory and antioxidant stress, and then ameliorating kidney fibrosis and kidney failure. More research is needed on the relationship between LKD, vitamin B6 metabolism, and CKD.

Thus, we came up with a new insight of ‘low protein diet supplemented with α-ketoacid through gut microbiome and metabolome’ in CKD. We speculated that LKD may modulate the gut microbiome and fecal metabolites, which may play an important role in the amelioration of CKD. However, the interactions of microbiome and host metabolism tend to be intricate. Therefore, exploration studies and functional validation are needed to verify the mechanisms in the future.

There are several limitations to the study. First, although the features of 5/6Nx mice were consistent with patients with CKD, the sample size was relatively small, which may impact the stability of results. Second, 16s rRNA sequencing and untargeted metabolomics had limited extent and accuracy in the annotation of species and metabolites. Future studies need to be done to establish the exact relationship between LKD and alterations in gut microbiome and metabolome.

## Conclusion

Protein restriction is central, although controversial, in dietary intervention for CKD. Our study indicates that an LPD supplemented with α-ketoacid plays a more reno-protective and anti-inflammation role than an LPD alone in the 5/6Nx mice model. The effect may be mediated by modulating the gut microbiota and fecal metabolome. These results provide new insights into targeting diet-gut microbiota-metabolism for the treatment of CKD and also need more studies to determine the exact mechanisms.

## Data Availability Statement

The data presented in the study are deposited in the (NCBI SRA) repository, accession number (PRJNA810785).

## Ethics Statement

The animal study was reviewed and approved by Institutional Ethics Board of Fudan University Minhang Hospital.

## Author Contributions

YZ, HH, and XX were responsible for the design and implementation of the project. YZ, HH, and YT were in charge of the experimental part. YP and PH were responsible for data statistics and analysis. YZ and YT were responsible for the drawing and writing of the article. WS, PL, and MJ were responsible for collecting data and revising and organizing the article. All authors contributed to the article and approved the submitted version.

## Funding

This study was supported by grants from the National Natural Science Foundation of China (no. 81774060), the Shanghai Health and Planning Commission Scientific Research Foundation (no. 20184Y0040), the Shanghai Minhang District Characteristic Specialty Construction Project (no. 2020MWTZB07), and the Shanghai Minhang District High-level Specialty Backbone Physician Training Program Funding Project (no. 2020MZYS19).

## Conflict of Interest

The authors declare that the research was conducted in the absence of any commercial or financial relationships that could be construed as a potential conflict of interest.

## Publisher's Note

All claims expressed in this article are solely those of the authors and do not necessarily represent those of their affiliated organizations, or those of the publisher, the editors and the reviewers. Any product that may be evaluated in this article, or claim that may be made by its manufacturer, is not guaranteed or endorsed by the publisher.

## References

[1] Bikbov B Purcell CA Levey AS Smith M Abdoli A Abebe M. Global, regional, and national burden of chronic kidney disease, 1990-2017: a systematic analysis for the Global Burden of Disease Study 2017. Lancet. (2020) 395:709–33. 32061315 10.1016/S0140-6736(20)30045-3 PMC7049905

[2] Weiner ID Mitch WE Sands JM. Urea and ammonia metabolism and the control of renal nitrogen excretion. Clin J Am Soc Nephrol. (2015) 10:1444–58. 10.2215/CJN.10311013 25078422 PMC4527031

[3] Hostetter TH Meyer TW Rennke HG Brenner BM. Chronic effects of dietary protein in the rat with intact and reduced renal mass. Kidney Int. (1986) 30:509–17. 10.1038/ki.1986.215 3784291

[4] Brunori G Viola BF Parrinello G De Biase V Como G Franco V. Efficacy and safety of a very-low-protein diet when postponing dialysis in the elderly: A prospective randomized multicenter controlled study. Am J Kidney Dis. (2007) 49:569–80. 10.1053/j.ajkd.2007.02.278 17472838

[5] Ikizler TA Burrowes JD Byham-Gray LD Campbell KL Carrero JJ Chan W. KDOQI Clinical Practice Guideline for Nutrition in CKD: 2020 Update. Am J Kidney Dis. (2020) 76:S1–107. 10.1053/j.ajkd.2020.05.006 32829751

[6] Gao X Wu J Dong Z Hua C Hu H Mei C. low-protein diet supplemented with ketoacids plays a more protective role against oxidative stress of rat kidney tissue with 5/6 nephrectomy than a low-protein diet alone. Br J Nutr. (2010) 103:608–16. 10.1017/S0007114509992108 19878616

[7] Bellizzi V Di Iorio BR De Nicola L Minutolo R Zamboli P Trucillo P. Very low protein diet supplemented with ketoanalogs improves blood pressure control in chronic kidney disease. Kidney Int. (2007) 71:245–51. 10.1038/sj.ki.5001955 17035939

[8] Tang WH Wang Z Kennedy DJ Wu Y Buffa JA Agatisa-Boyle B. Gut microbiota-dependent trimethylamine N-oxide (TMAO) pathway contributes to both development of renal insufficiency and mortality risk in chronic kidney disease. Circ Res. (2015) 116:448–55. 10.1161/CIRCRESAHA.116.305360 25599331 PMC4312512

[9] Wu IW Gao SS Chou HC Yang HY Chang LC Kuo YL. Integrative metagenomic and metabolomic analyses reveal severity-specific signatures of gut microbiota in chronic kidney disease. Theranostics. (2020) 10:5398–411. 10.7150/thno.41725 32373220 PMC7196299

[10] Wang X Yang S Li S Zhao L Hao Y Qin J. Aberrant gut microbiota alters host metabolome and impacts renal failure in humans and rodents. Gut. (2020) 69:2131–42. 10.1136/gutjnl-2019-319766 32241904 PMC7677483

[11] Conlon MA Bird AR. The impact of diet and lifestyle on gut microbiota and human health. Nutrients. (2014) 7:17–44. 10.3390/nu7010017 25545101 PMC4303825

[12] Hasan AA von Websky K Reichetzeder C Tsuprykov O Gaballa M Guo J. Mechanisms of GLP-1 receptor-independent renoprotective effects of the dipeptidyl peptidase type 4 inhibitor linagliptin in GLP-1 receptor knockout mice with 5/6 nephrectomy. Kidney Int. (2019) 95:1373–88. 10.1016/j.kint.2019.01.010 30979564

[13] Magoc T Salzberg SL. FLASH Fast length adjustment of short reads to improve genome assemblies. Bioinformatics. (2011) 27:2957–63. 10.1093/bioinformatics/btr507 21903629 PMC3198573

[14] Caporaso JG Kuczynski J Stombaugh J Bittinger K Bushman FD Costello EK. QIIME allows analysis of high-throughput community sequencing data. Nat Methods. (2010) 7:335–6. 10.1038/nmeth.f.303 20383131 PMC3156573

[15] Edgar RC Haas BJ Clemente JC Quince C Knight R. UCHIME improves sensitivity and speed of chimera detection. Bioinformatics. (2011) 27:2194–200. 10.1093/bioinformatics/btr381 21700674 PMC3150044

[16] Haas BJ Gevers D Earl AM Feldgarden M Ward DV Giannoukos G. Chimeric 16S rRNA sequence formation and detection in Sanger and 454-pyrosequenced PCR amplicons. Genome Res. (2011) 21:494–504. 10.1101/gr.112730.110 21212162 PMC3044863

[17] Quast C Pruesse E Yilmaz P Gerken J Schweer T Yarza P. The SILVA ribosomal RNA gene database project: Improved data processing and web-based tools. Nucleic Acids Res. (2013) 41:D590–6. 10.1093/nar/gks1219 23193283 PMC3531112

[18] Lobel L Cao YG Fenn K Glickman JN Garrett WS. Diet posttranslationally modifies the mouse gut microbial proteome to modulate renal function. Science. (2020) 369:1518–24. 10.1126/science.abb3763 32943527 PMC8178816

[19] Sonnenburg JL Backhed F. Diet-microbiota interactions as moderators of human metabolism. Nature. (2016) 535:56–64. 10.1038/nature18846 27383980 PMC5991619

[20] Gao X Huang L Grosjean F Esposito V Wu J Fu L. Low-protein diet supplemented with ketoacids reduces the severity of renal disease in 5/6 nephrectomized rats: A role for KLF15. Kidney Int. (2011) 79:987–96. 10.1038/ki.2010.539 21248717 PMC3332106

[21] Zmora N Suez J Elinav E. You are what you eat: Diet, health and the gut microbiota. Nat Rev Gastroenterol Hepatol. (2019) 16:35–56. 10.1038/s41575-018-0061-2 30262901

[22] Ren Z Fan Y Li A Shen Q Wu J Ren L. Alterations of the human gut microbiome in chronic kidney disease. Adv Sci (Weinh). (2020) 7:2001936. 10.1002/advs.202001936 33101877 PMC7578882

[23] Li J Zhao F Wang Y Chen J Tao J Tian G. Gut microbiota dysbiosis contributes to the development of hypertension. Microbiome. (2017) 5:14. 10.1186/s40168-016-0222-x 28143587 PMC5286796

[24] Spychala MS Venna VR Jandzinski M Doran SJ Durgan DJ Ganesh BP. Age-related changes in the gut microbiota influence systemic inflammation and stroke outcome. Ann Neurol. (2018) 84:23–36. 10.1002/ana.25250 29733457 PMC6119509

[25] Wehedy E Shatat IF Al KS. The human microbiome in chronic kidney disease: A Double-Edged sword. Front Med (Lausanne). (2021) 8:790783. 10.3389/fmed.2021.790783 35111779 PMC8801809

[26] Hu X Xie Y Xiao Y Zeng W Gong Z Du J. Longitudinal analysis of fecal microbiome and metabolome during renal fibrotic progression in a unilateral ureteral obstruction animal model. Eur J Pharmacol. (2020) 886:173555. 10.1016/j.ejphar.2020.173555 32937112

[27] Zhang J Guo Z Xue Z Sun Z Zhang M Wang L. A phylo-functional core of gut microbiota in healthy young Chinese cohorts across lifestyles, geography and ethnicities. ISME J. (2015) 9:1979–90. 10.1038/ismej.2015.11 25647347 PMC4542028

[28] Poesen R Viaene L Verbeke K Claes K Bammens B Sprangers B. Renal clearance and intestinal generation of p-cresyl sulfate and indoxyl sulfate in CKD. Clin J Am Soc Nephrol. (2013) 8:1508–14. 10.2215/CJN.00300113 23813557 PMC3805062

[29] Di Iorio BR Marzocco S Bellasi A De Simone E Dal Piaz F Rocchetti MT. Nutritional therapy reduces protein carbamylation through urea lowering in chronic kidney disease. Nephrol Dial Transplant. (2018) 33:804–13. 10.1093/ndt/gfx203 28992314

[30] Mo Y Sun H Zhang L Geng W Wang L Zou C. Microbiome-Metabolomics analysis reveals the protection mechanism of alpha-Ketoacid on Adenine-Induced chronic kidney disease in rats. Front Pharmacol. (2021) 12:657827. 10.3389/fphar.2021.657827 34045965 PMC8144710

[31] Li F Wang M Wang J Li R Zhang Y. Alterations to the gut microbiota and their correlation with inflammatory factors in chronic kidney disease. Front Cell Infect Microbiol. (2019) 9:206. 10.3389/fcimb.2019.00206 31245306 PMC6581668

[32] Lai S Molfino A Testorio M Perrotta AM Currado A Pintus G. Effect of low-protein diet and inulin on microbiota and clinical parameters in patients with chronic kidney disease. Nutrients. (2019) 11:3006. 10.3390/nu11123006 31818021 PMC6950025

[33] Ju T Kong JY Stothard P Willing BP. Defining the role of Parasutterella, a previously uncharacterized member of the core gut microbiota. ISME J. (2019) 13:1520–34. 10.1038/s41396-019-0364-5 30742017 PMC6776049

[34] Zhai Q Feng S Arjan N Chen W. A next generation probiotic, Akkermansia muciniphila. Crit Rev Food Sci Nutr. (2019) 59:3227–36. 10.1080/10408398.2018.1517725 30373382

[35] Depommier C Everard A Druart C Plovier H Van Hul M Vieira-Silva S. Supplementation with Akkermansia muciniphila in overweight and obese human volunteers: A proof-of-concept exploratory study. Nat Med. (2019) 25:1096–103. 10.1038/s41591-019-0495-2 31263284 PMC6699990

[36] Plovier H Everard A Druart C Depommier C Van Hul M Geurts L. A purified membrane protein from Akkermansia muciniphila or the pasteurized bacterium improves metabolism in obese and diabetic mice. Nat Med. (2017) 23:107–13. 10.1038/nm.4236 27892954

[37] Wang L Tang L Feng Y Zhao S Han M Zhang C. A purified membrane protein from Akkermansia muciniphila or the pasteurised bacterium blunts colitis associated tumourigenesis by modulation of CD8(+) T cells in mice. Gut. (2020) 69:1988–97. 10.1136/gutjnl-2019-320105 32169907 PMC7569398

[38] Liang JQ Li T Nakatsu G Chen YX Yau TO Chu E. A novel faecal Lachnoclostridium marker for the non-invasive diagnosis of colorectal adenoma and cancer. Gut. (2020) 69:1248–57. 10.1136/gutjnl-2019-318532 31776231 PMC7306980

[39] Zhou L Ni Z Yu J Cheng W Cai Z Yu C. Correlation between fecal metabolomics and gut microbiota in obesity and polycystic ovary syndrome. Front Endocrinol (Lausanne). (2020) 11:628. 10.3389/fendo.2020.00628 33013704 PMC7505924

[40] Li Y Su X Zhang L Liu Y Shi M Lv C. Dysbiosis of the gut microbiome is associated with CKD5 and correlated with clinical indices of the disease: a case-controlled study. J Transl Med. (2019) 17:228. 10.1186/s12967-019-1969-1 31315634 PMC6637476

[41] Zhang ZH Chen H Vaziri ND Mao JR Zhang L Bai X. Metabolomic signatures of chronic kidney disease of diverse etiologies in the rats and humans. J Proteome Res. (2016) 15:3802–12. 10.1021/acs.jproteome.6b00583 27636000

[42] Gui T Li Y Zhang S Alecu I Chen Q Zhao Y. Oxidative stress increases 1-deoxysphingolipid levels in chronic kidney disease. Free Radic Biol Med. (2021) 164:139–48. 10.1016/j.freeradbiomed.2021.01.011 33450378

[43] Ponticelli C Podesta MA Moroni G. Hyperuricemia as a trigger of immune response in hypertension and chronic kidney disease. Kidney Int. (2020) 98:1149–59. 10.1016/j.kint.2020.05.056 32650020

[44] Mishima E Ichijo M Kawabe T Kikuchi K Akiyama Y Toyohara T. Germ-Free conditions modulate host purine metabolism, exacerbating Adenine-Induced kidney damage. Toxins (Basel). (2020) 12:547. 10.3390/toxins12090547 32859011 PMC7551802

[45] Gondouin B Jourde-Chiche N Sallee M Dou L Cerini C Loundou A. Plasma xanthine oxidase activity is predictive of cardiovascular disease in patients with chronic kidney disease, independently of uric acid levels. Nephron. (2015) 131:167–74. 10.1159/000441091 26426087

[46] Wang M Xu H Chong LSO Li L Gao H Zhao Z. Compound alpha-keto acid tablet supplementation alleviates chronic kidney disease progression via inhibition of the NF-kB and MAPK pathways. J Transl Med. (2019) 17:122. 10.1186/s12967-019-1856-9 30975176 PMC6458753

[47] Rapa SF Di Iorio BR Campiglia P Heidland A Marzocco S. Inflammation and oxidative stress in chronic kidney Disease-Potential therapeutic role of minerals, vitamins and Plant-Derived metabolites. Int J Mol Sci. (2019) 21:263. 10.3390/ijms21010263 31906008 PMC6981831

[48] Li Y Luo ZY Hu YY Bi YW Yang JM Zou WJ. The gut microbiota regulates autism-like behavior by mediating vitamin B6 homeostasis in EphB6-deficient mice. Microbiome. (2020) 8:120. 10.1186/s40168-020-00884-z 32819434 PMC7441571

[49] Williams ME Bolton WK Khalifah RG Degenhardt TP Schotzinger RJ McGill JB. Effects of pyridoxamine in combined phase 2 studies of patients with type 1 and type 2 diabetes and overt nephropathy. Am J Nephrol. (2007) 27:605–14. 10.1159/000108104 17823506

[50] Lewis EJ Greene T Spitalewiz S Blumenthal S Berl T Hunsicker LG. Pyridorin in type 2 diabetic nephropathy. J Am Soc Nephrol. (2012) 23:131–6. 10.1681/ASN.2011030272 22034637 PMC3269925

[51] Skrypnyk NI Voziyan P Yang H de Caestecker CR Theberge MC Drouin M. Pyridoxamine reduces postinjury fibrosis and improves functional recovery after acute kidney injury. Am J Physiol Renal Physiol. (2016) 311:F268–77. 10.1152/ajprenal.00056.2016 27194713 PMC5008672

[52] Ebert T Neytchev O Witasp A Kublickiene K Stenvinkel P Shiels PG. Inflammation and oxidative stress in chronic kidney disease and dialysis patients. Antioxid Redox Signal. (2021) 35:1426–48. 10.1089/ars.2020.8184 34006115

[53] Kopple JD. Abnormal amino acid and protein metabolism in uremia. Kidney Int. (1978) 14:340–8. 10.1038/ki.1978.134 366228

[54] Xie Y Pan H Liu M Xiao X Shi Y. Progress in asymmetric biomimetic transamination of carbonyl compounds. Chem Soc Rev. (2015) 44:1740–8. 10.1039/C4CS00507D 25645264

[55] Robertson BR O'Rourke JL Neilan BA Vandamme P On S Fox JG. Mucispirillum schaedleri gen. Nov, Sp Nov, A spiral-shaped bacterium colonizing the mucus layer of the gastrointestinal tract of laboratory rodents. Int J Syst Evol Microbiol. (2005) 55:1199–204. 10.1099/ijs.0.63472-0 15879255

