# Supplementary material for: Reno-Protective Effect of Low Protein Diet Supplemented With α-Ketoacid Through Gut Microbiota and Fecal Metabolism in 5/6 Nephrectomized Mice
Source: Front Nutr. 2022 Jun 30;9:889131. doi: 10.3389/fnut.2022.889131 (PMC9280408; doi:10.3389/fnut.2022.889131)
Supplement: Supplementary file 2 [file Data_Sheet_2.docx]

Supplementary Material

# Supplementary Data

**Supplementary materials of the untargeted fecal metabolomic analysis**

**Metabolites Extraction**

25 mg of fecal sample was weighted to an EP tube, and 500 μL extract solution (methanol: acetonitrile: water = 2: 2: 1, with isotopically-labeled internal standard mixture) was added. Then the samples were homogenized at 35 Hz for 4 min and sonicated for 5 min in an ice water bath. The homogenization and sonication cycle was repeated for 3 times. Then the samples were incubated for 1 h at -40°C and centrifuged at 12000 rpm for 15 min at 4°C. The resulting supernatant was transferred to a fresh glass vial for analysis. The quality control (QC) sample was prepared by mixing an equal aliquot of the supernatants from all of the samples.

**LC-MS/MS Analysis**

The liquid chromatography-tandem mass spectrometry (LC-MS/MS) analyses were performed using a UHPLC system (Vanquish, Thermo Fisher Scientific) with a Ultra-high performance liquid chromatography (UPLC) BEH Amide column (2.1 mm × 100 mm, 1.7 μm) coupled to Q Exactive HFX mass spectrometer (Orbitrap MS, Thermo). The mobile phase consisted of 25 mmol/L ammonium acetate and 25 ammonia hydroxide in water (pH = 9.75) (A) and acetonitrile (B). The auto-sampler temperature was 4°C, and the injection volume was 3μL. The QE HFX mass spectrometer was used for its ability to acquire MS/MS spectra on information-dependent acquisition (IDA) mode in the control of the acquisition software (Xcalibur, Thermo). In this mode, the acquisition software continuously evaluates the full scan MS spectrum. The ESI source conditions were set as following: sheath gas flow rate as 30 Arb, Aux gas flow rate as 25 Arb, capillary temperature 350°C, full MS resolution as 60000, MS/MS resolution as 7500, collision energy as 10/30/60 in NCE mode, spray Voltage as 3.6 kV (positive) or -3.2 kV (negative), respectively.

**Data preprocessing and annotation**

The raw data were converted to the mzXML format using ProteoWizard and processed with an in-house program, which was developed using R and based on XCMS, for peak detection, extraction, alignment, and integration. Then an in-house MS2 database (BiotreeDB) was applied in metabolite annotation. The cutoff for annotation was set at 0.3. For metabolites with the same annotation in positive and negative mode, we keep those with larger peaks and reject those with smaller peaks.

# Supplementary Figure 1


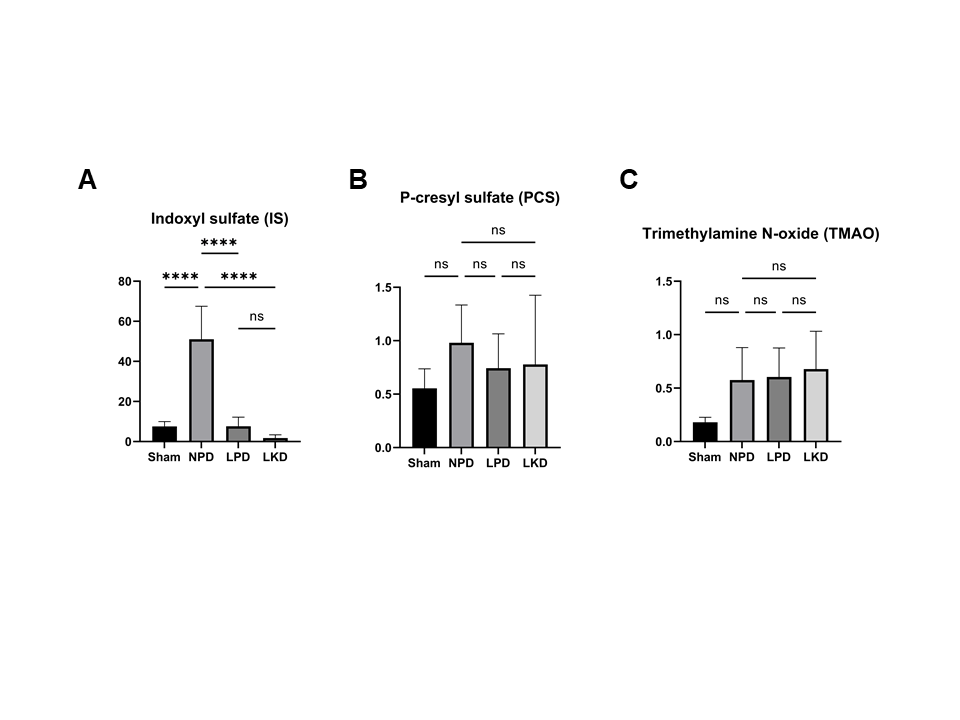


**Supplementary Figure 1.** The comparison of IS, PCS, and TMAO.

**(A)** The comparison of IS (Anova test).

**(B)** The comparison of PCS (Anova test).

**(C)** The comparison of IS (Anova test).

Ns, not significant; *P < 0.05;**P < 0.01;***P < 0.001;****P < 0.0001.
