# Supplementary figures and images for: Reno-Protective Effect of Low Protein Diet Supplemented With α-Ketoacid Through Gut Microbiota and Fecal Metabolism in 5/6 Nephrectomized Mice
Source: Front Nutr. 2022 Jun 30;9:889131. doi: 10.3389/fnut.2022.889131 (PMC9280408; doi:10.3389/fnut.2022.889131)

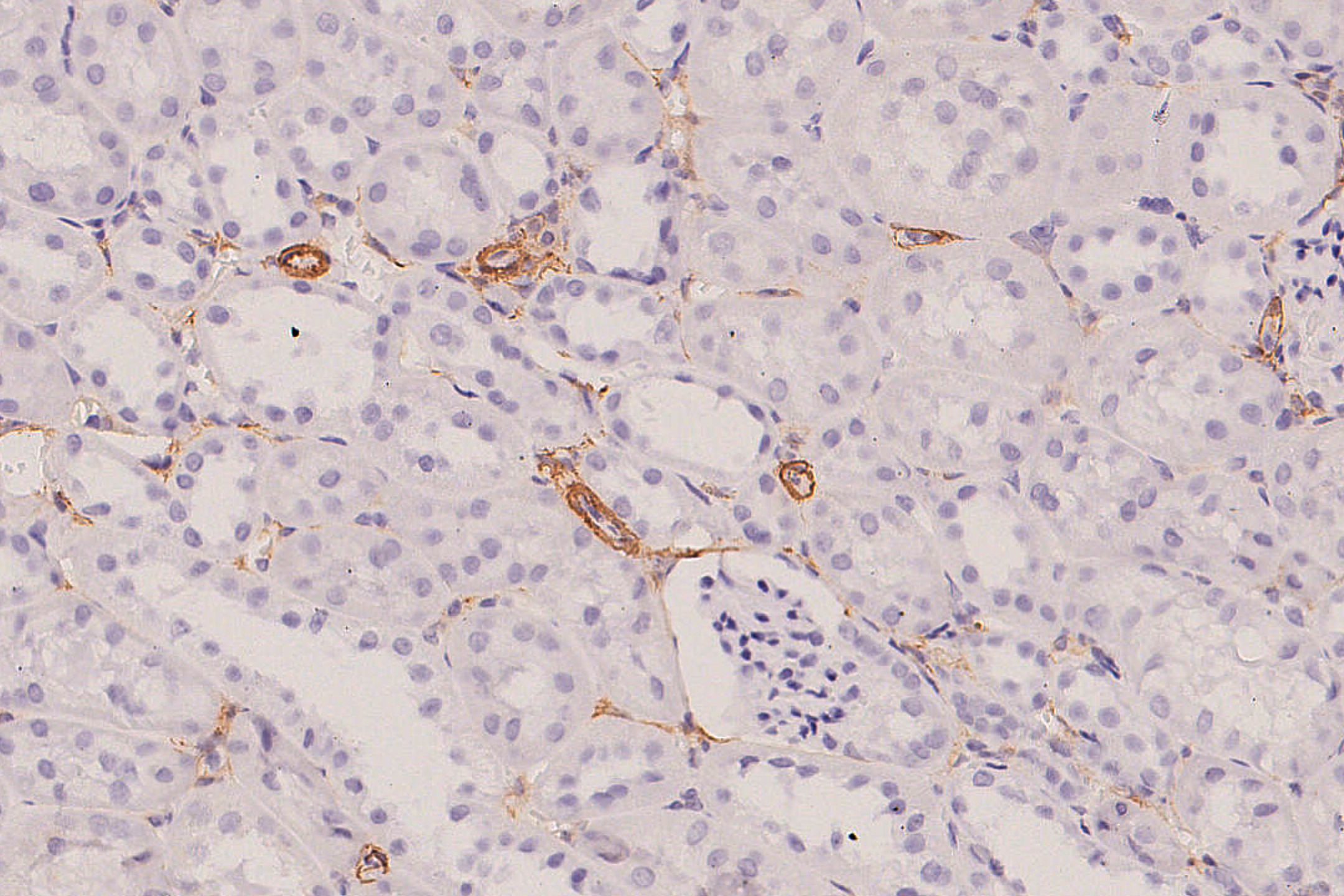

Supplement: Supplementary file 3 [file Data_Sheet_3.zip › LKD/LKD 1.jpg]

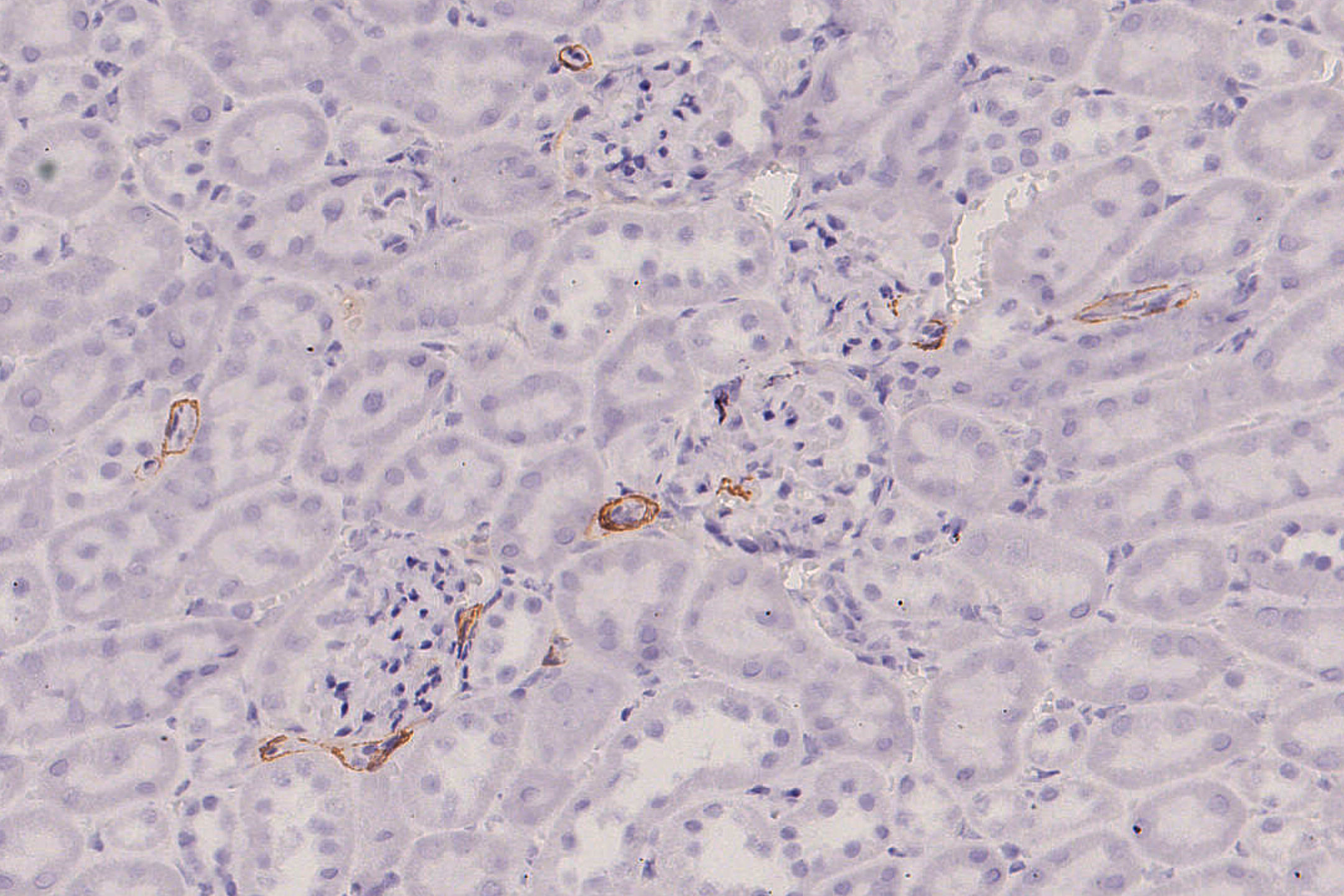

Supplement: Supplementary file 3 [file Data_Sheet_3.zip › LKD/LKD 4.jpg]

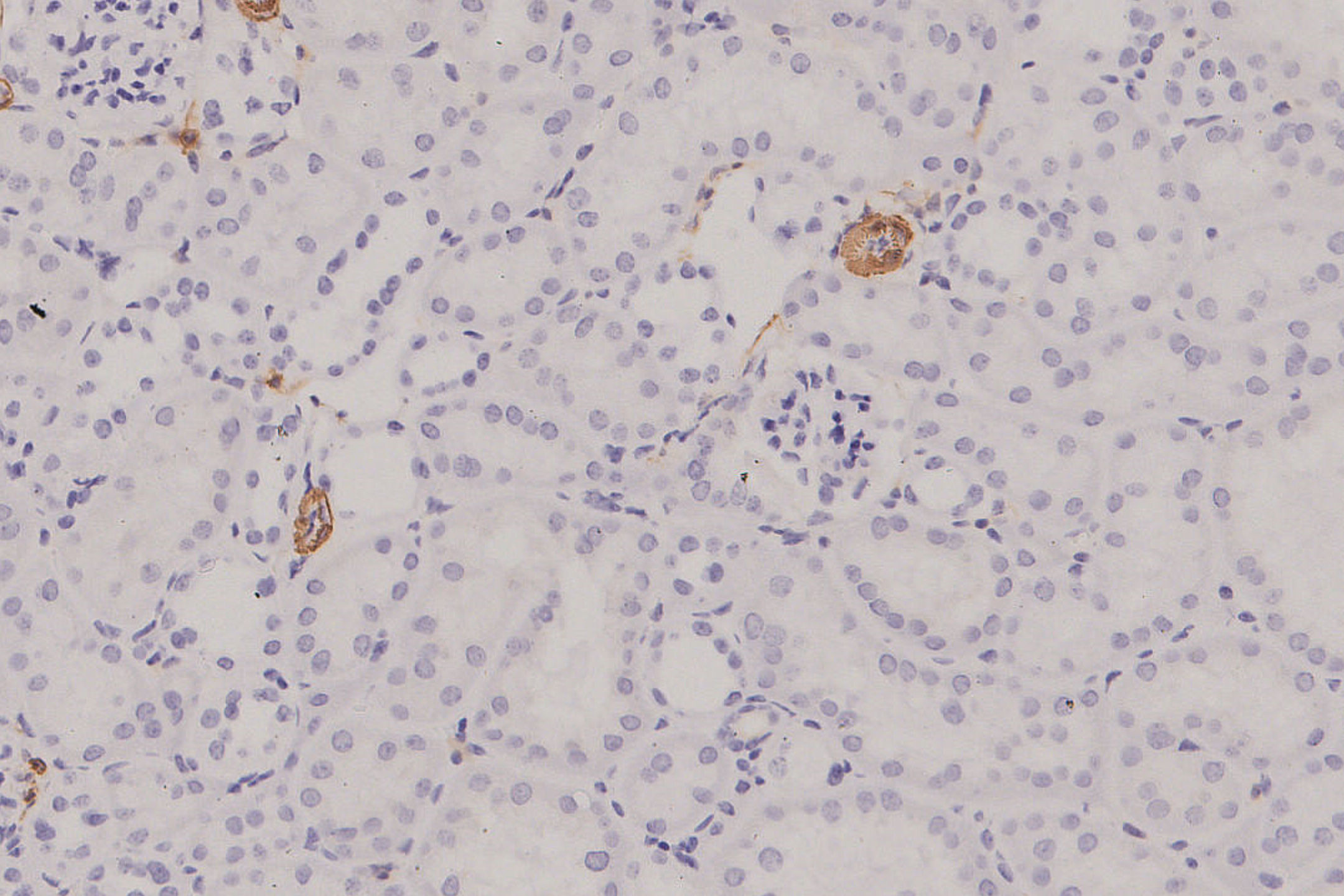

Supplement: Supplementary file 3 [file Data_Sheet_3.zip › LKD/LKD 5.jpg]

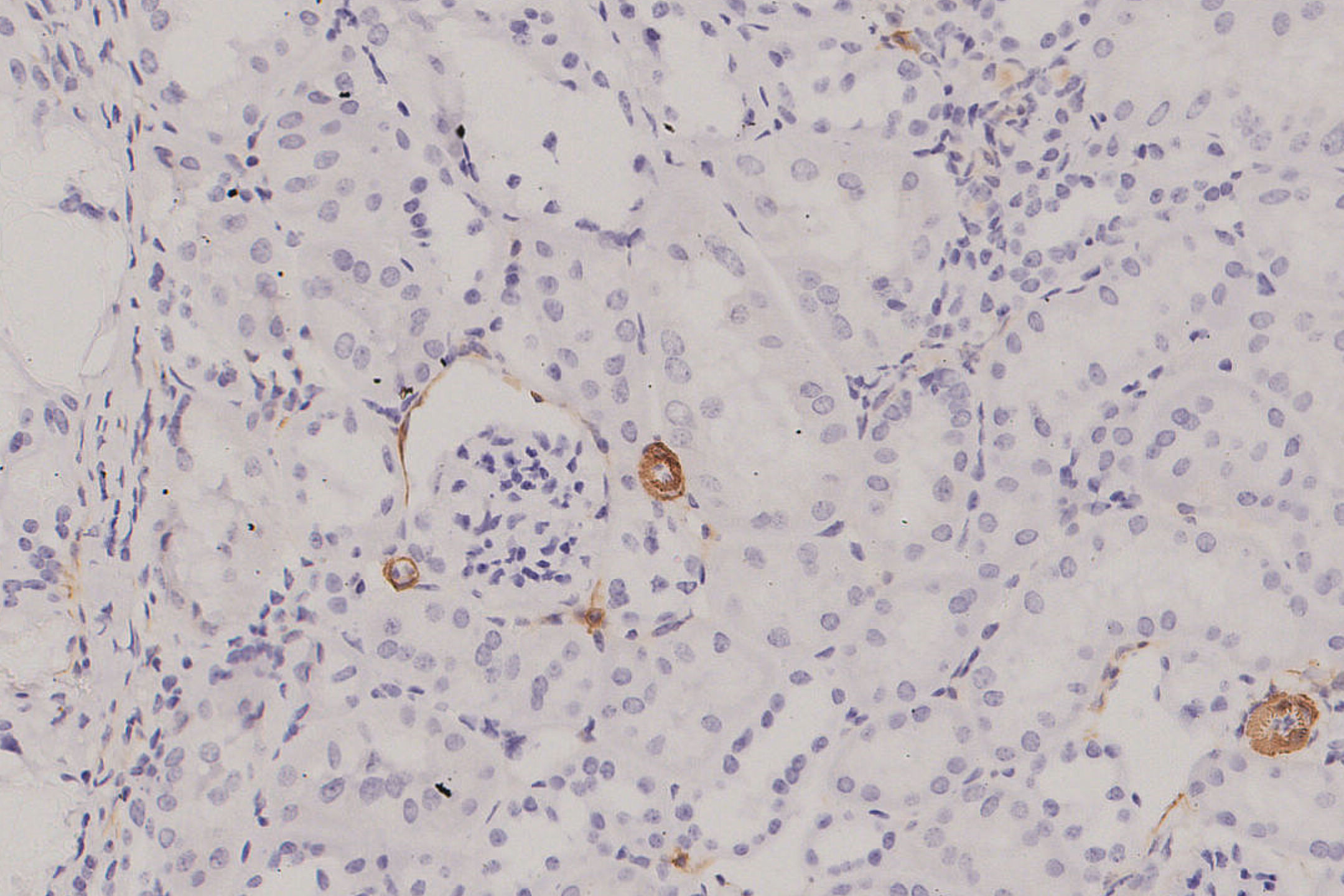

Supplement: Supplementary file 3 [file Data_Sheet_3.zip › LKD/LKD 6.jpg]

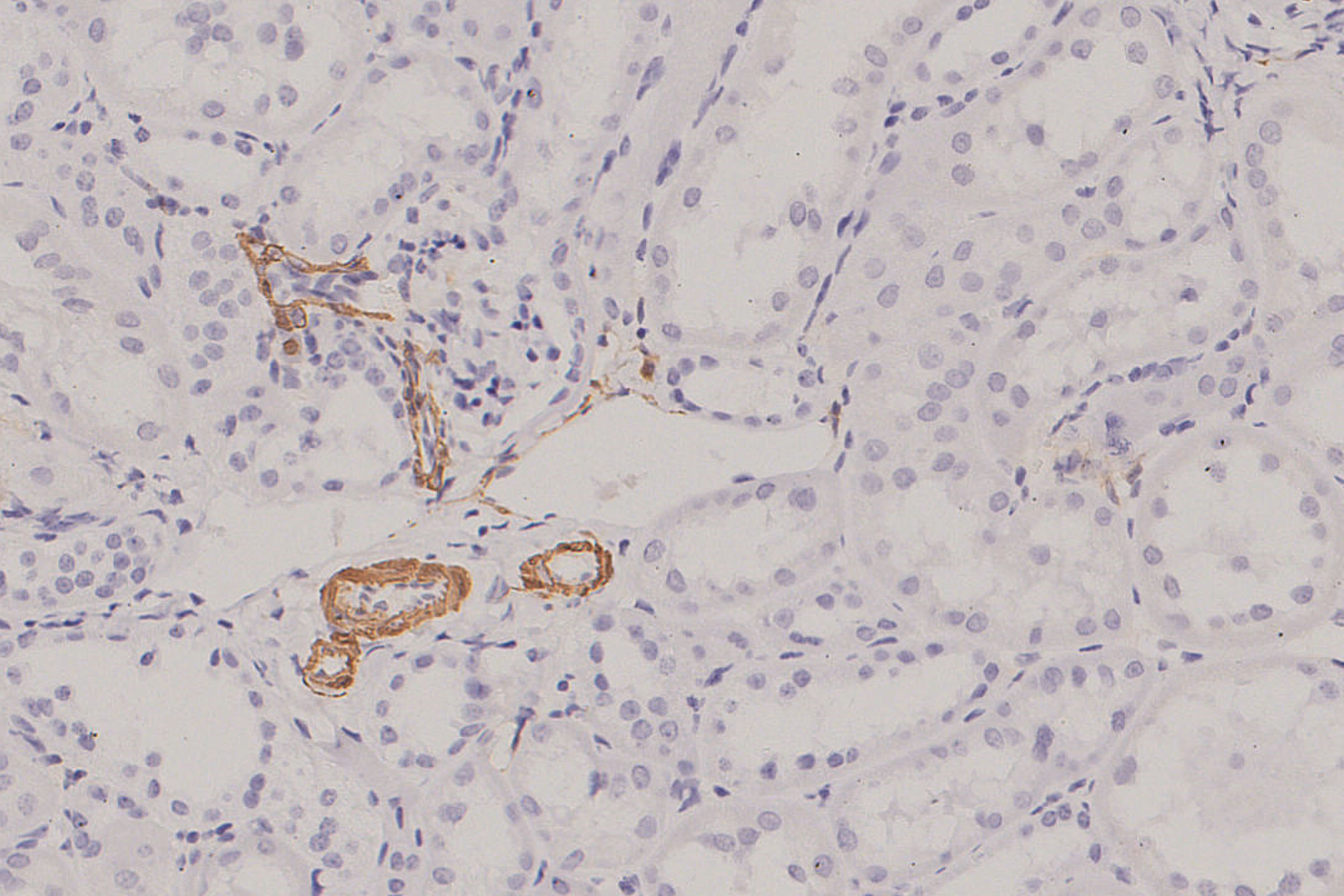

Supplement: Supplementary file 3 [file Data_Sheet_3.zip › LKD/LKD 7.jpg]

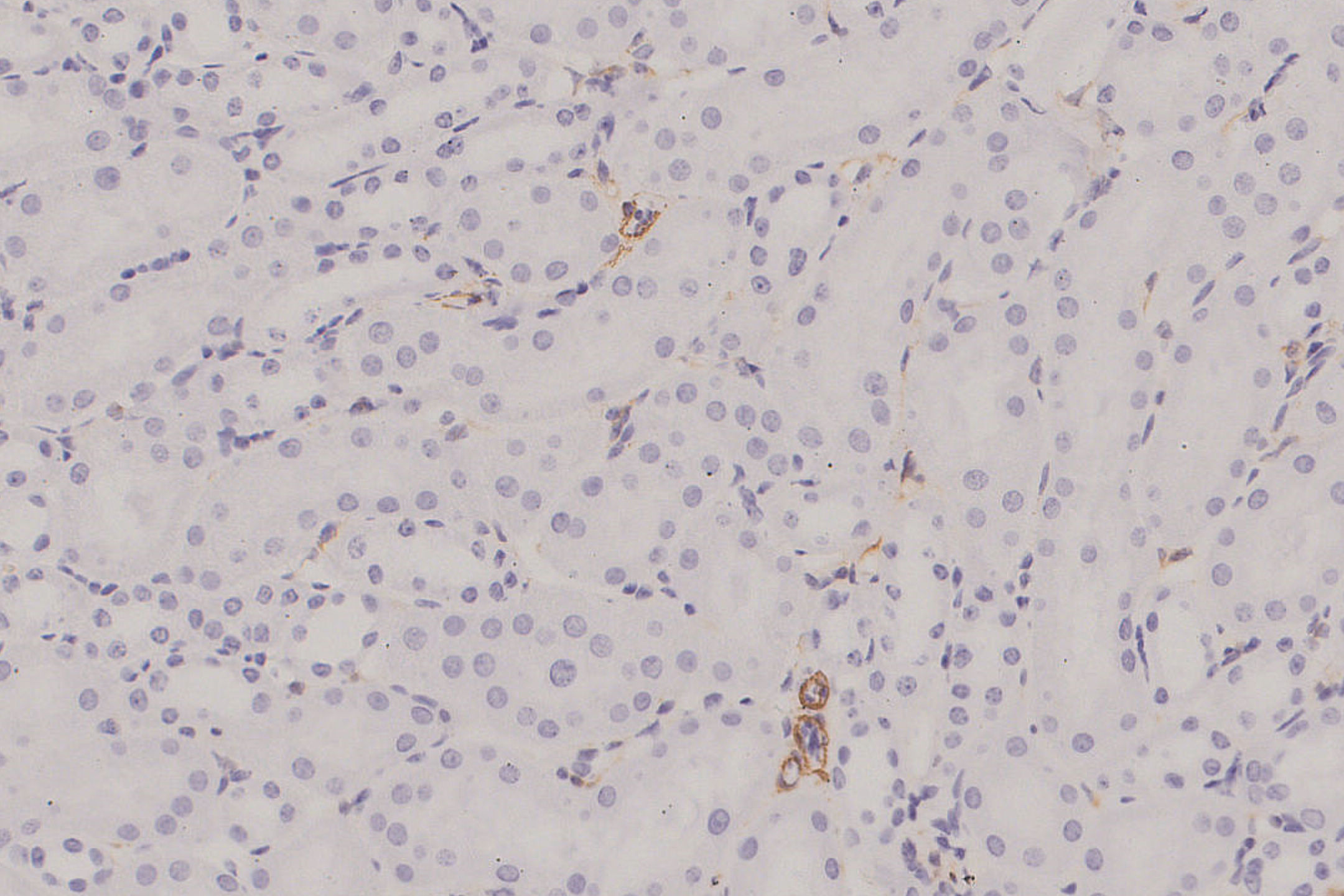

Supplement: Supplementary file 3 [file Data_Sheet_3.zip › LKD/LKD 8.jpg]

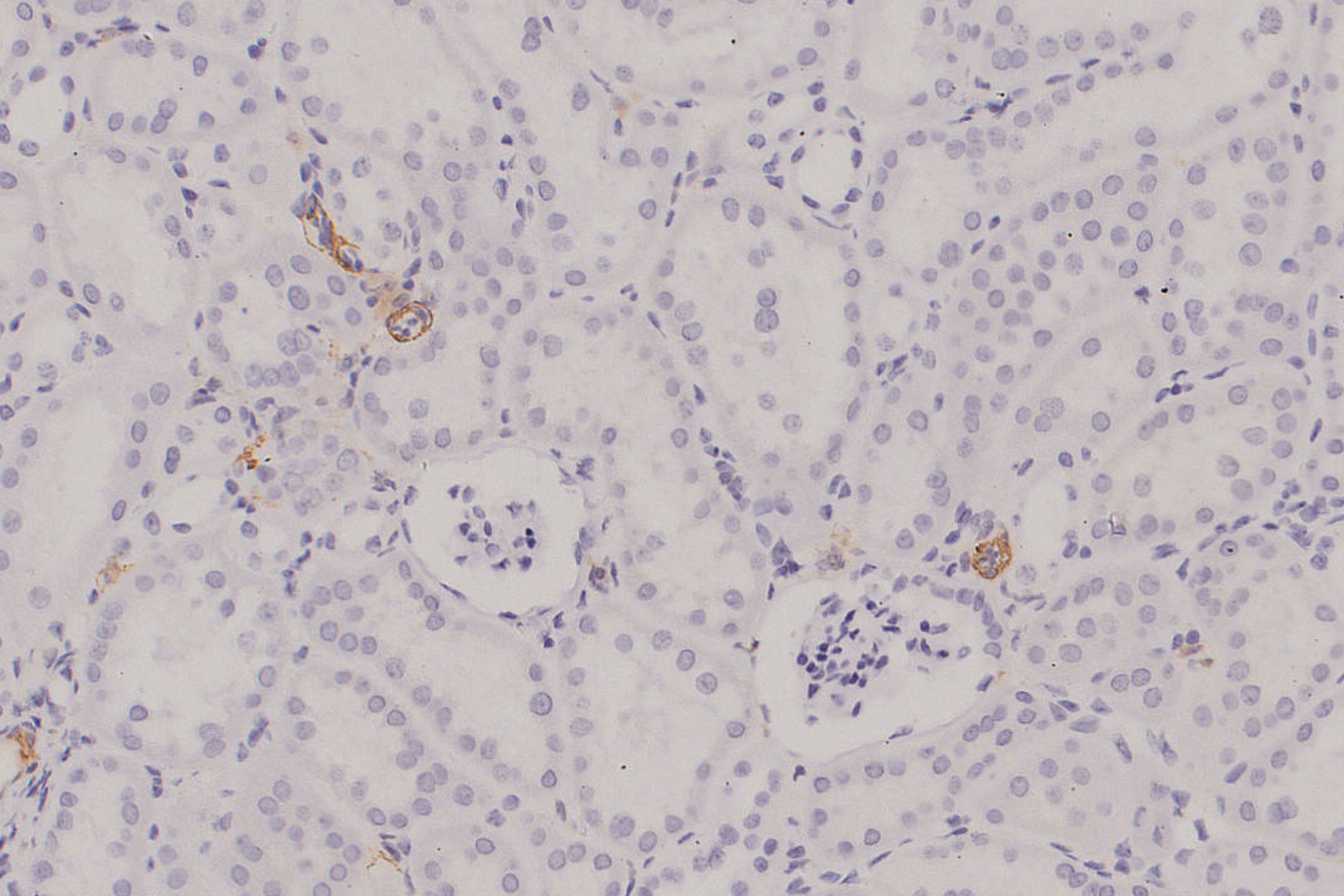

Supplement: Supplementary file 3 [file Data_Sheet_3.zip › LKD/LKD 9.jpg]

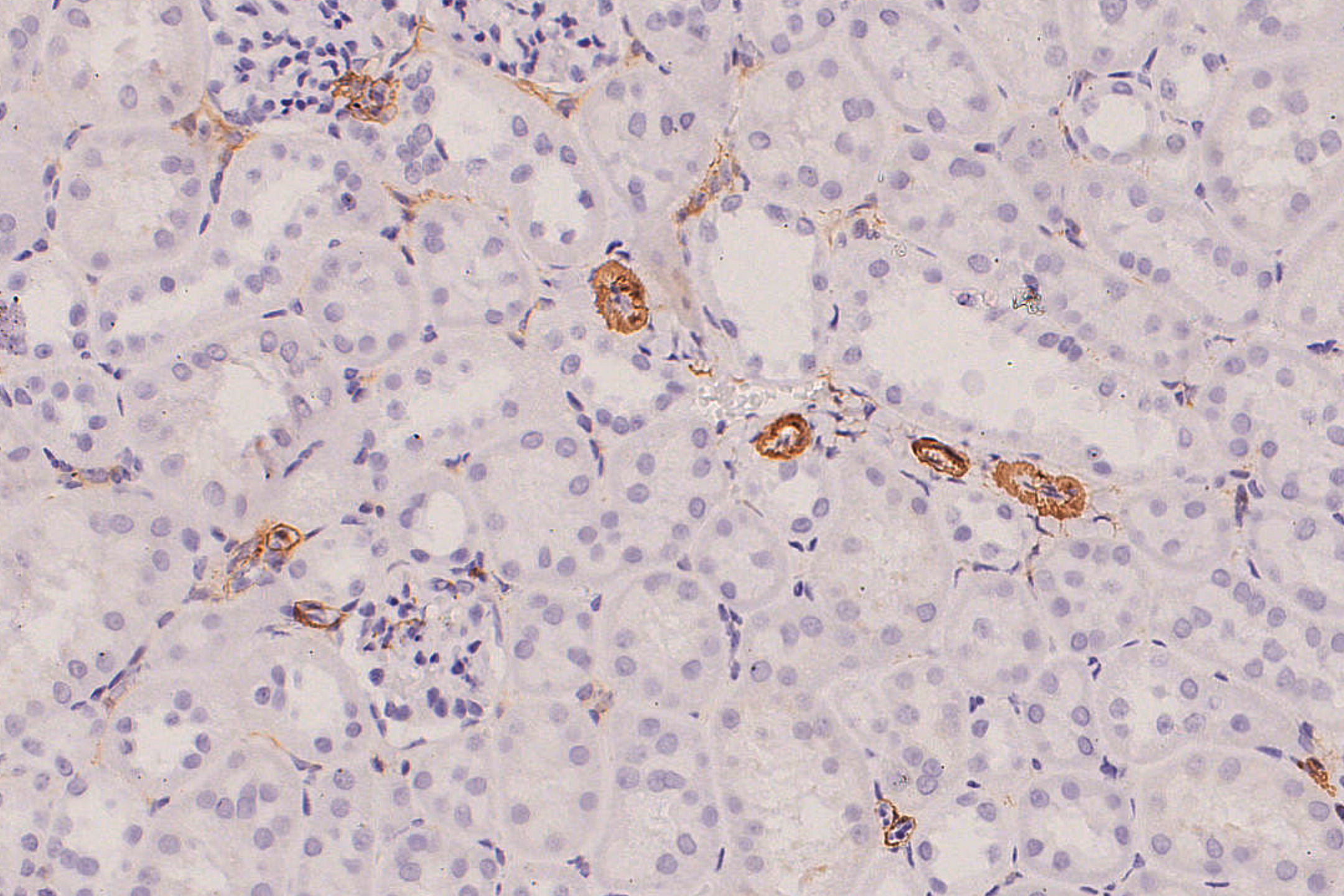

Supplement: Supplementary file 3 [file Data_Sheet_3.zip › LKD/LKD2.jpg]

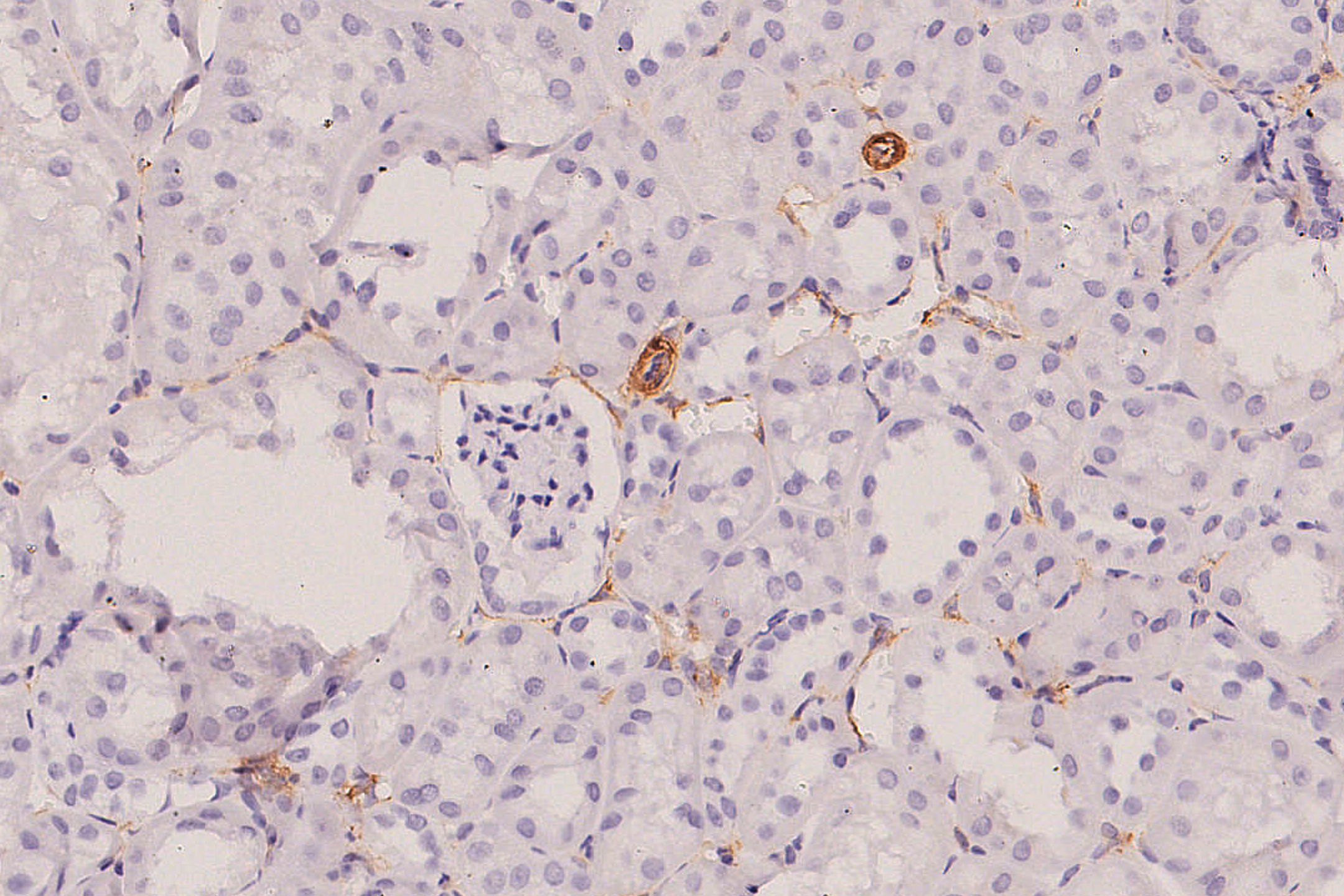

Supplement: Supplementary file 3 [file Data_Sheet_3.zip › LKD/LKD3.jpg]

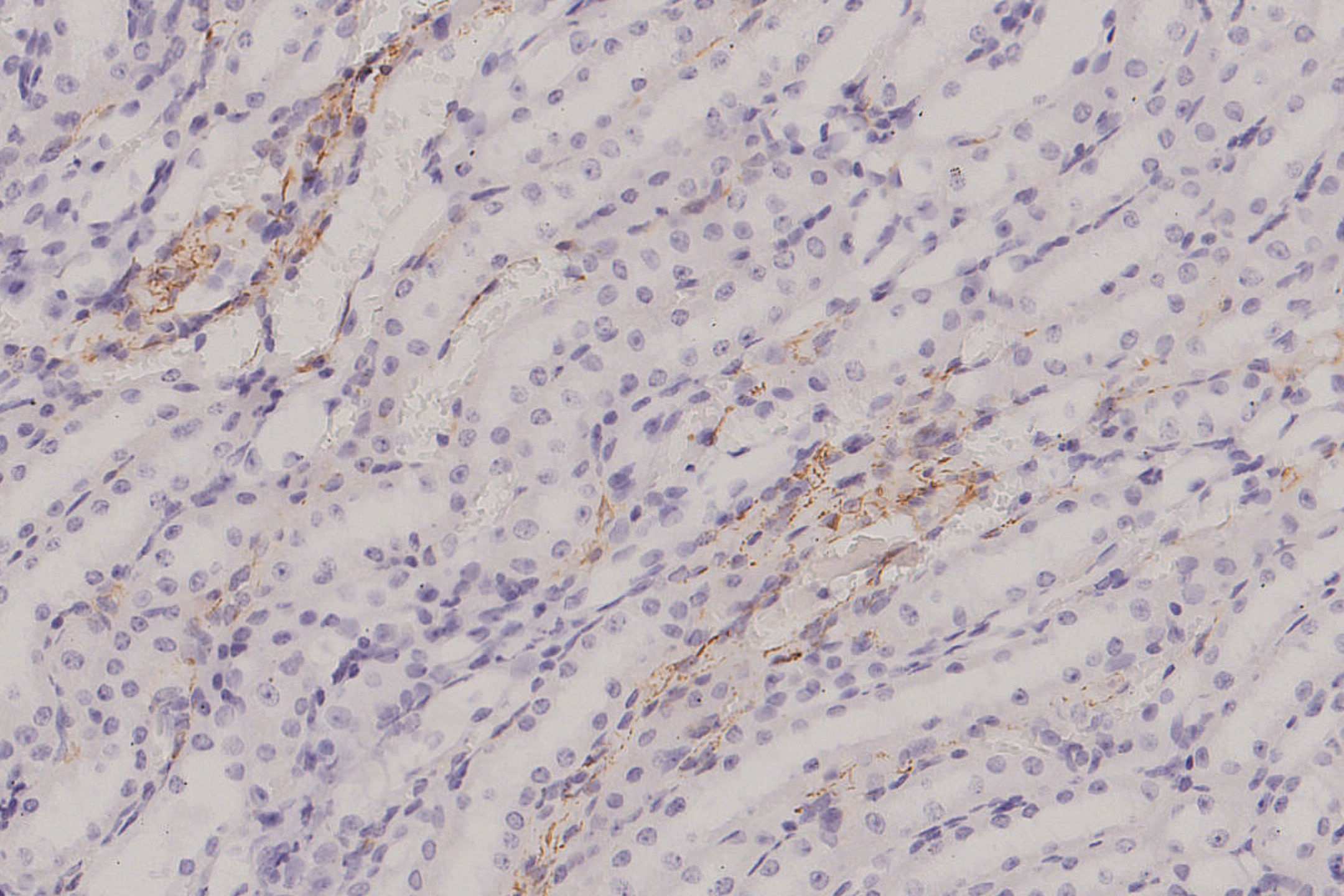

Supplement: Supplementary file 3 [file Data_Sheet_3.zip › LPD/LPD 1.jpg]

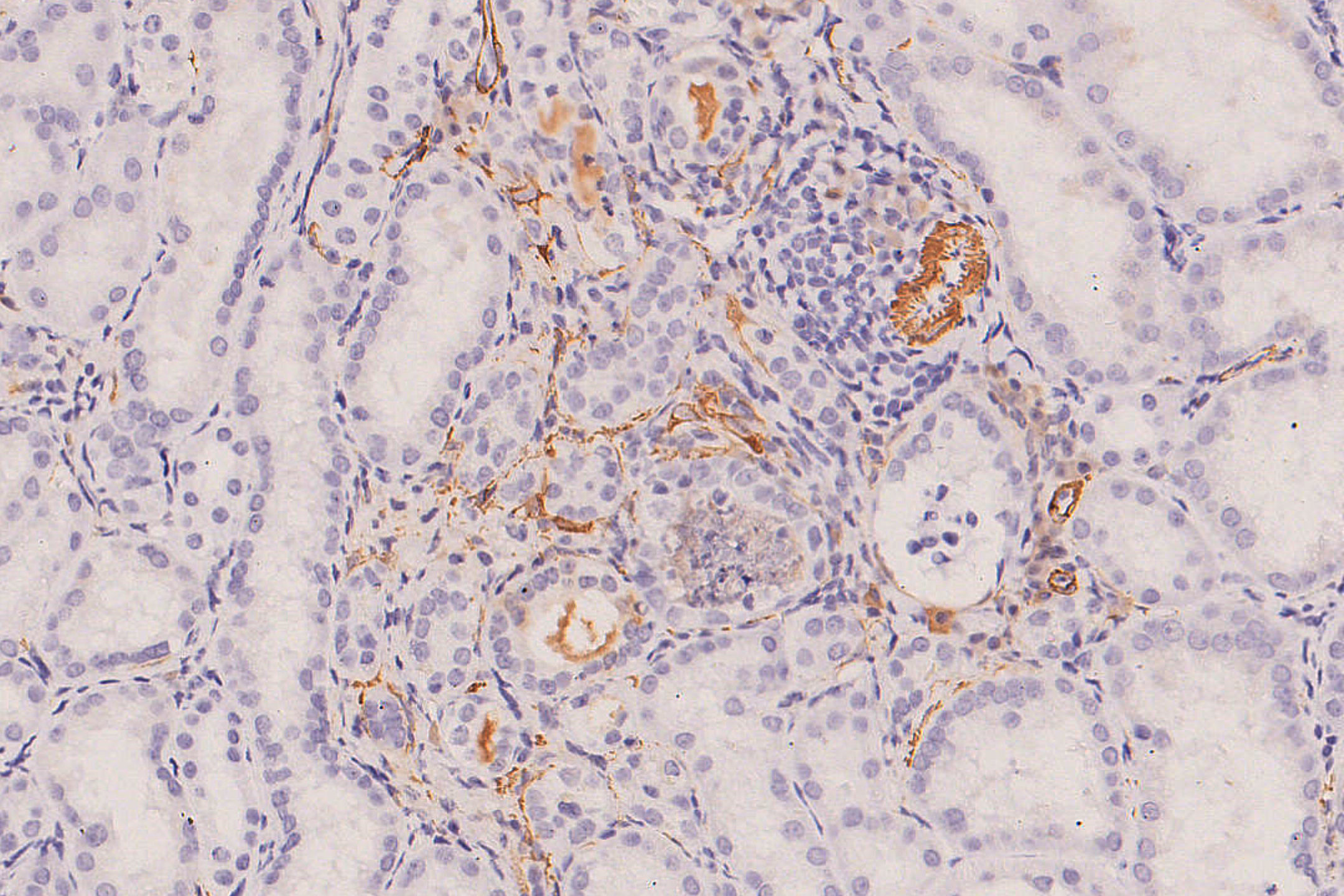

Supplement: Supplementary file 3 [file Data_Sheet_3.zip › LPD/LPD 2.jpg]

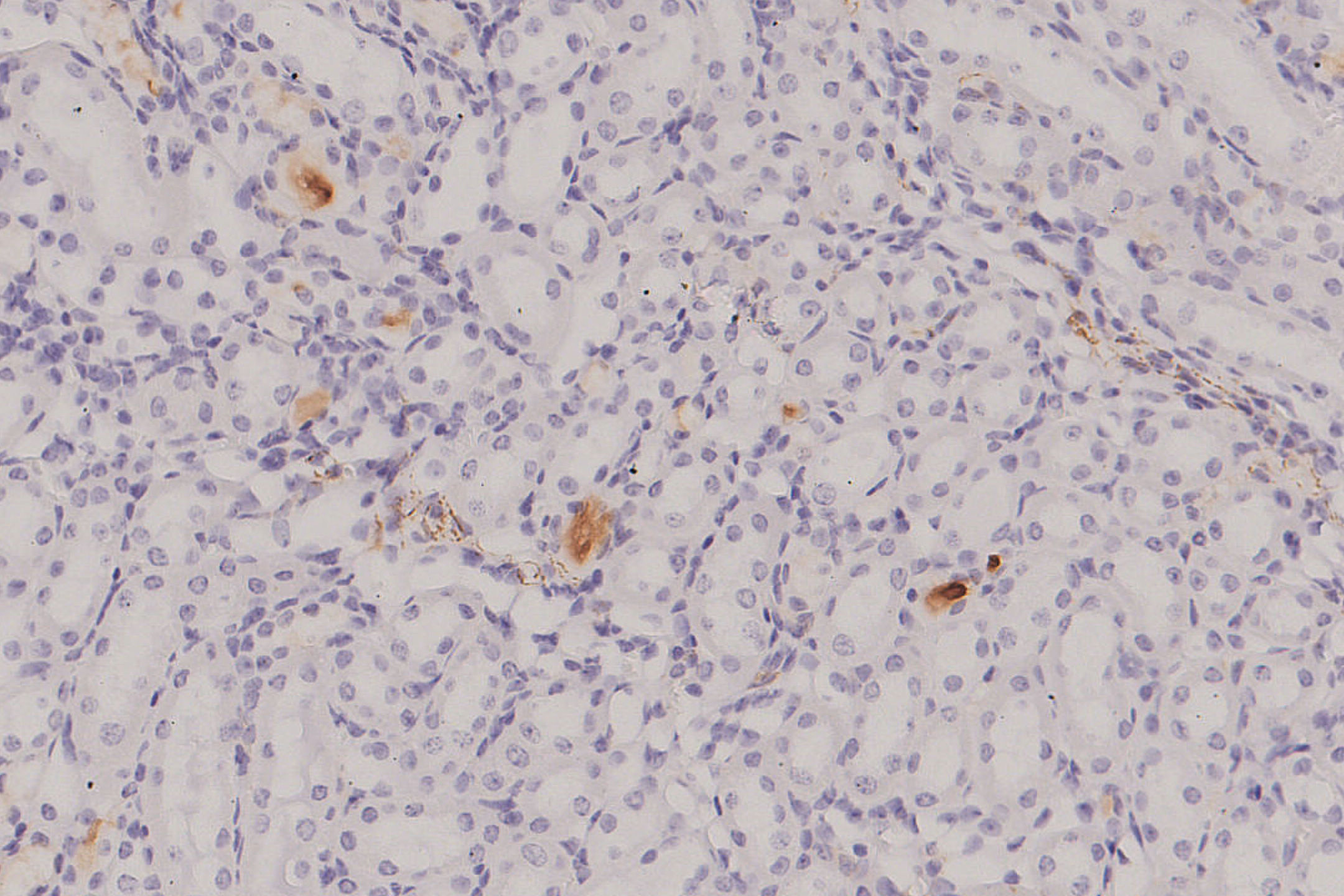

Supplement: Supplementary file 3 [file Data_Sheet_3.zip › LPD/LPD 3.jpg]

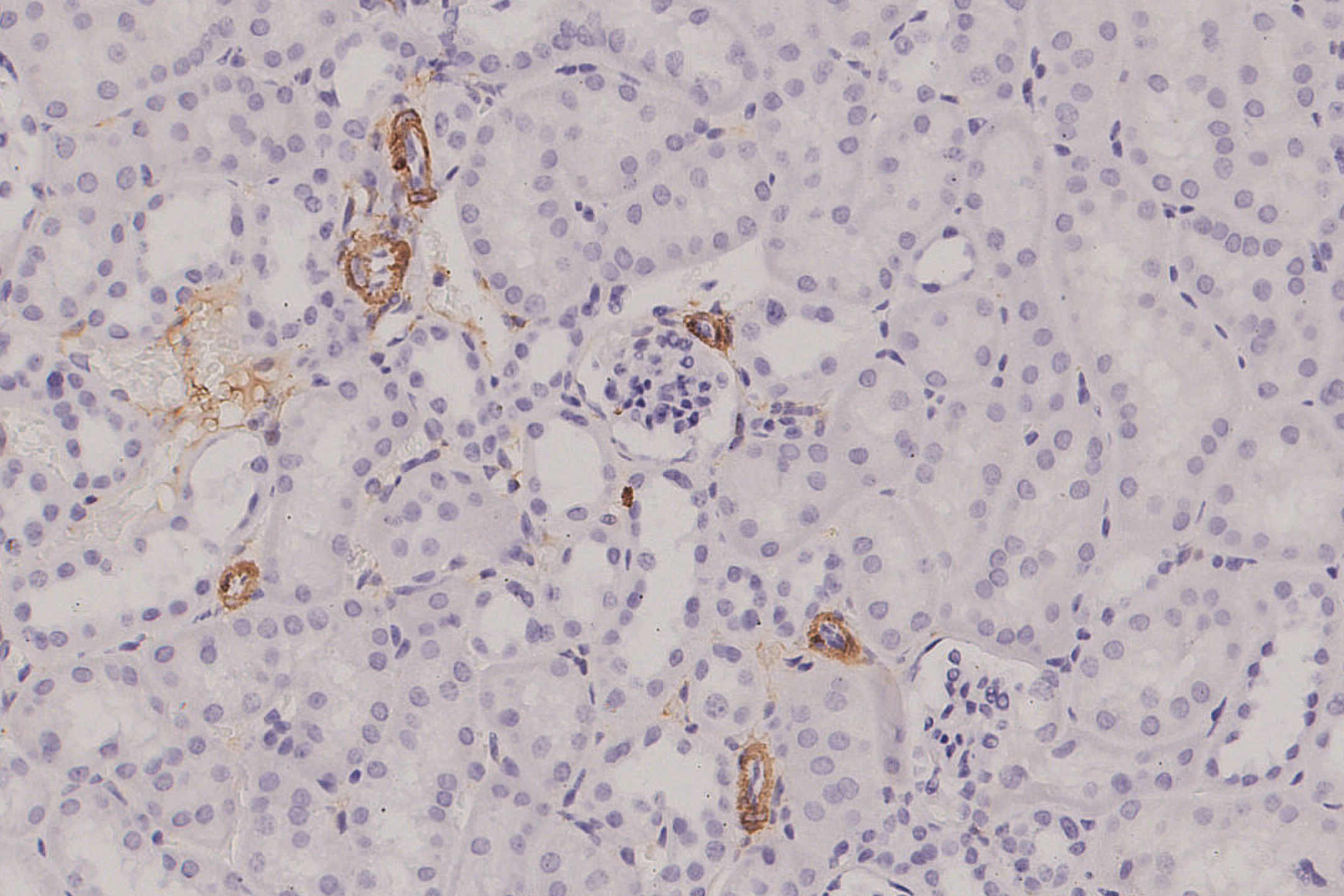

Supplement: Supplementary file 3 [file Data_Sheet_3.zip › LPD/LPD 4.jpg]

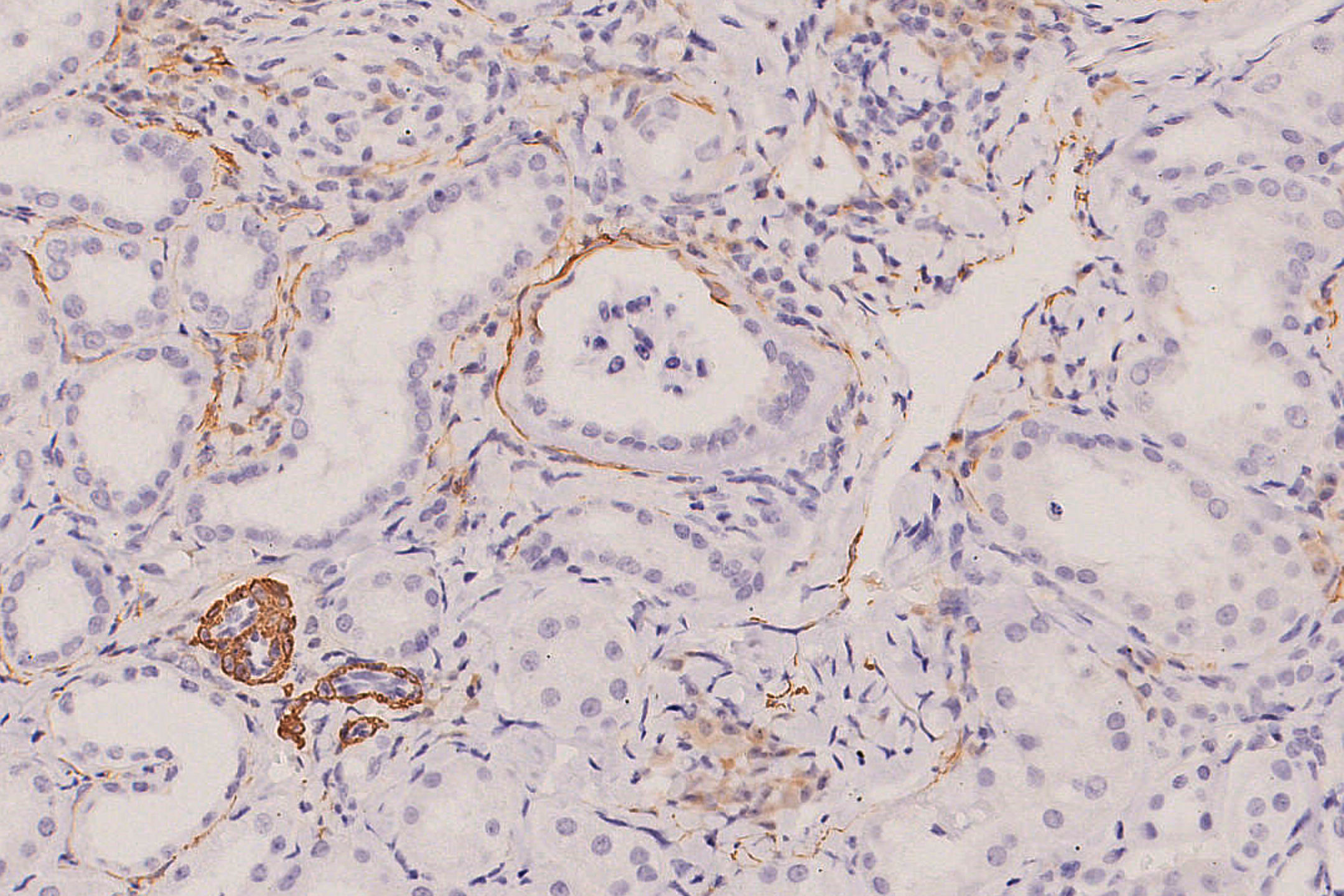

Supplement: Supplementary file 3 [file Data_Sheet_3.zip › LPD/LPD 6.jpg]

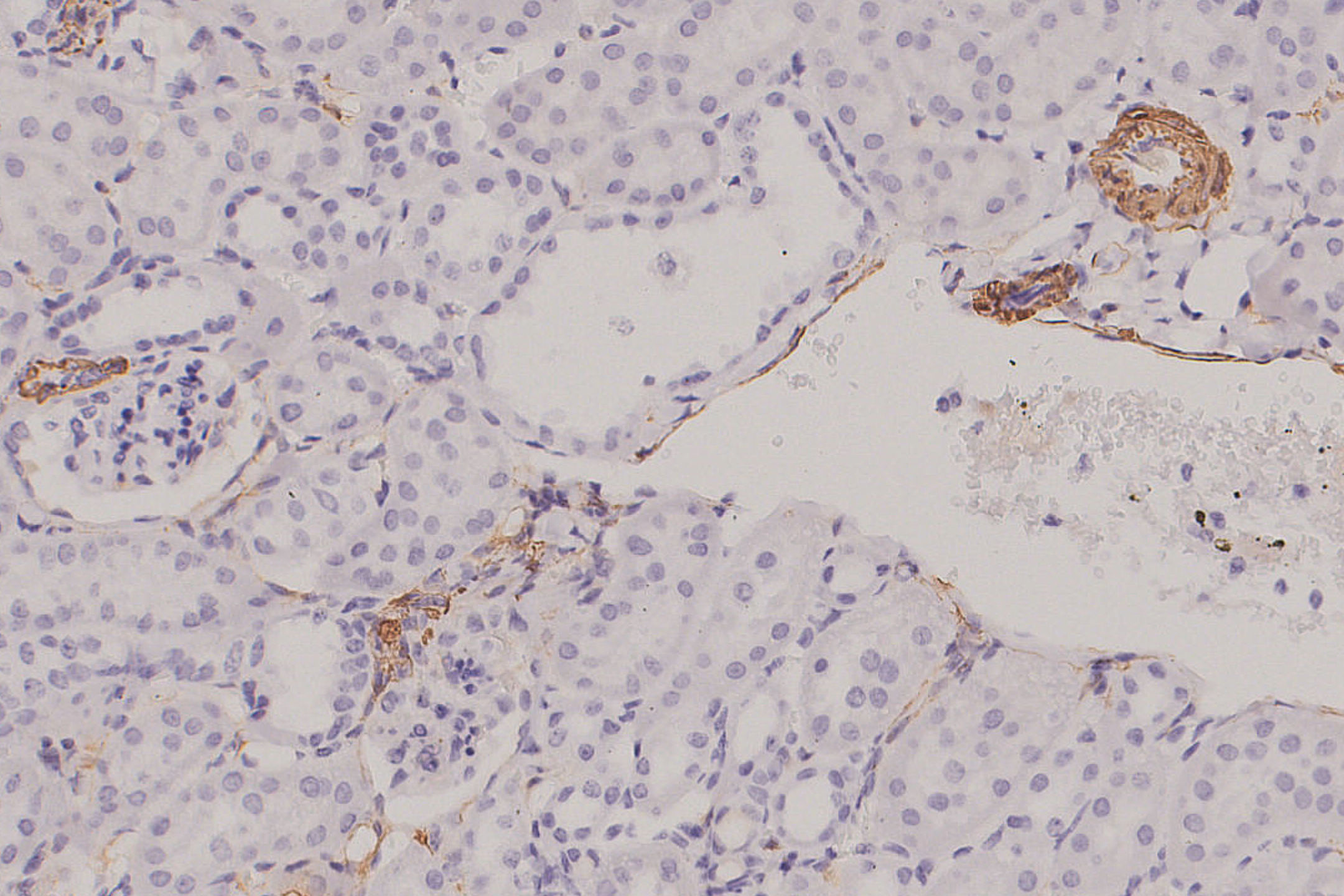

Supplement: Supplementary file 3 [file Data_Sheet_3.zip › LPD/LPD 7.jpg]

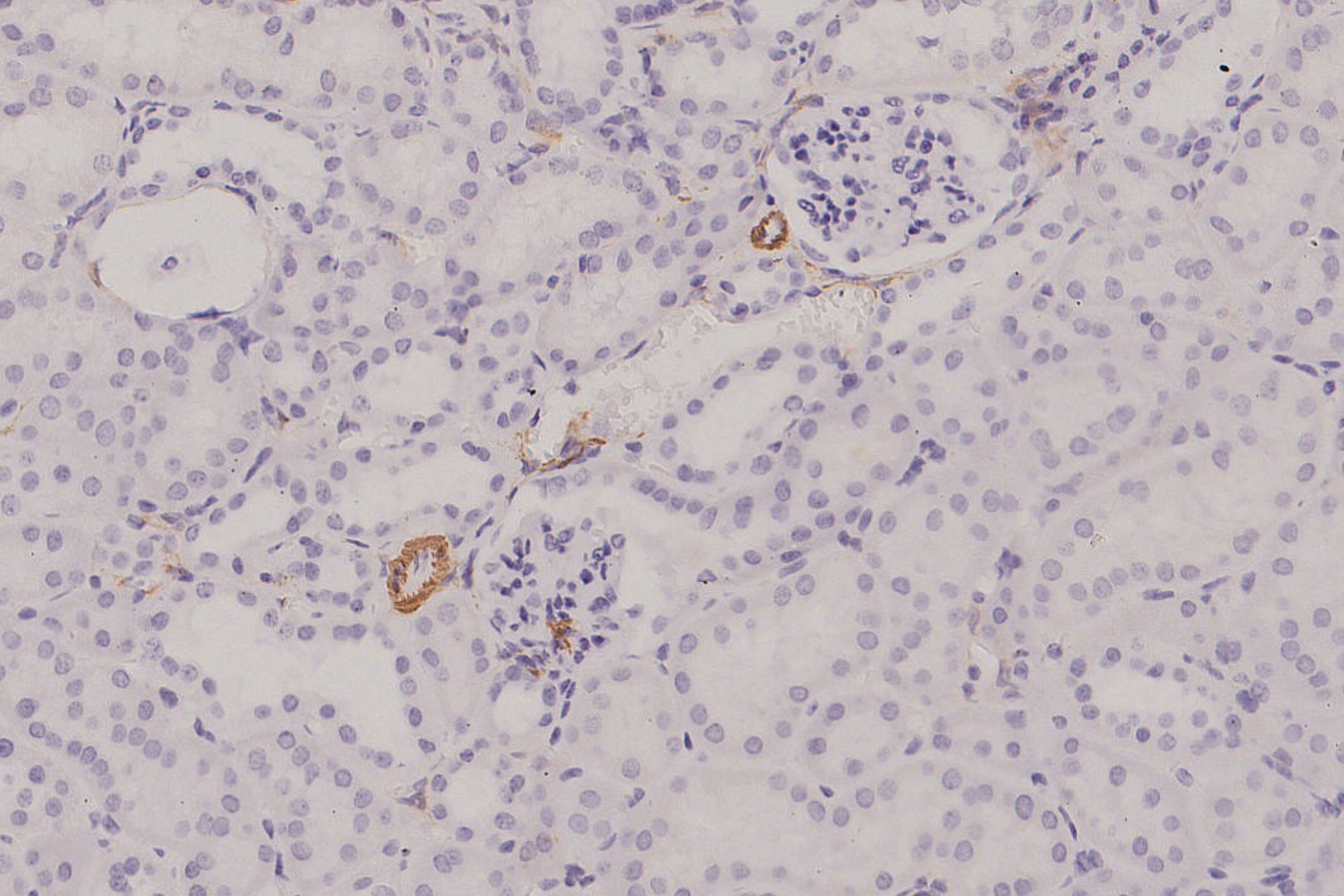

Supplement: Supplementary file 3 [file Data_Sheet_3.zip › LPD/LPD 8.jpg]

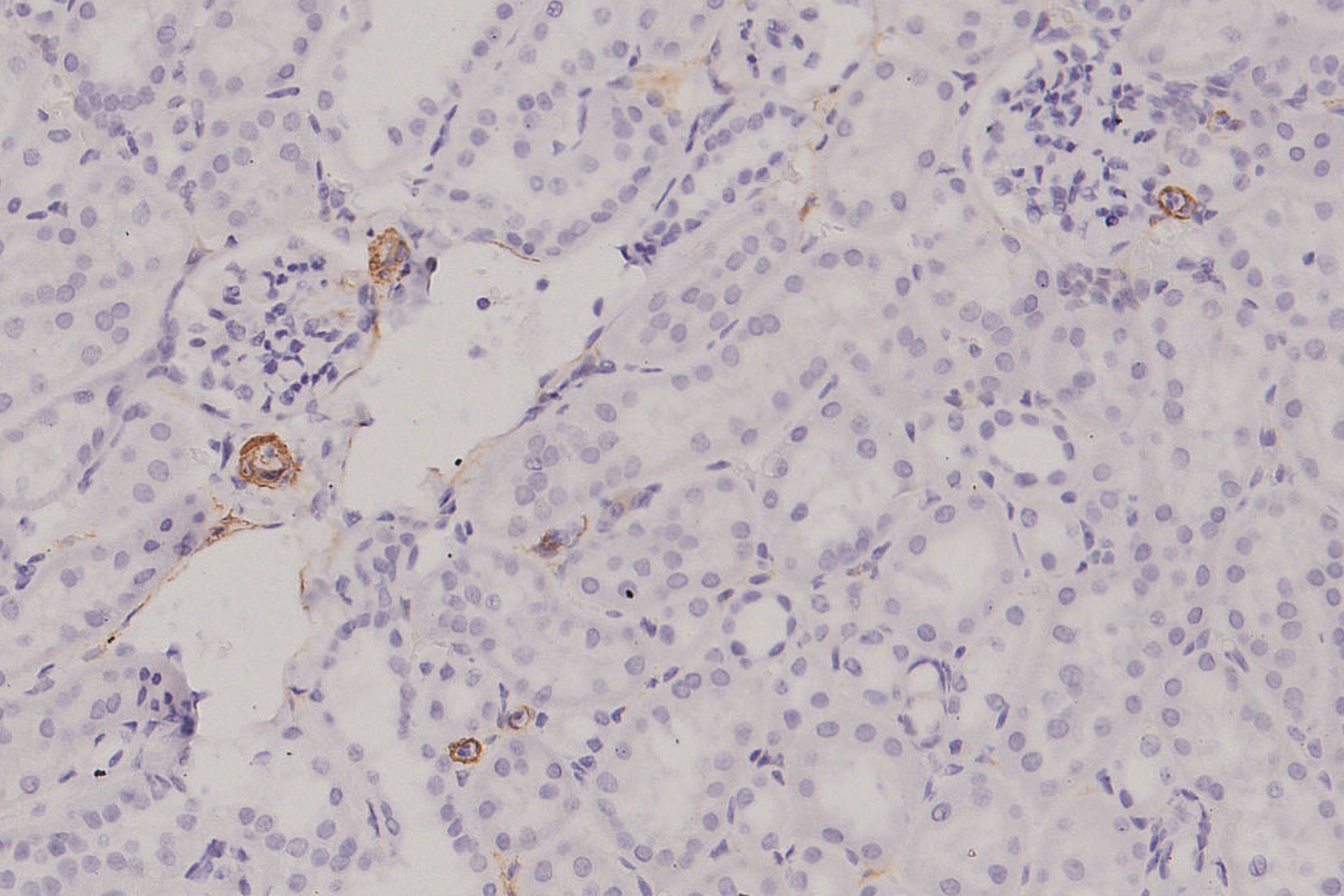

Supplement: Supplementary file 3 [file Data_Sheet_3.zip › LPD/LPD 9.jpg]

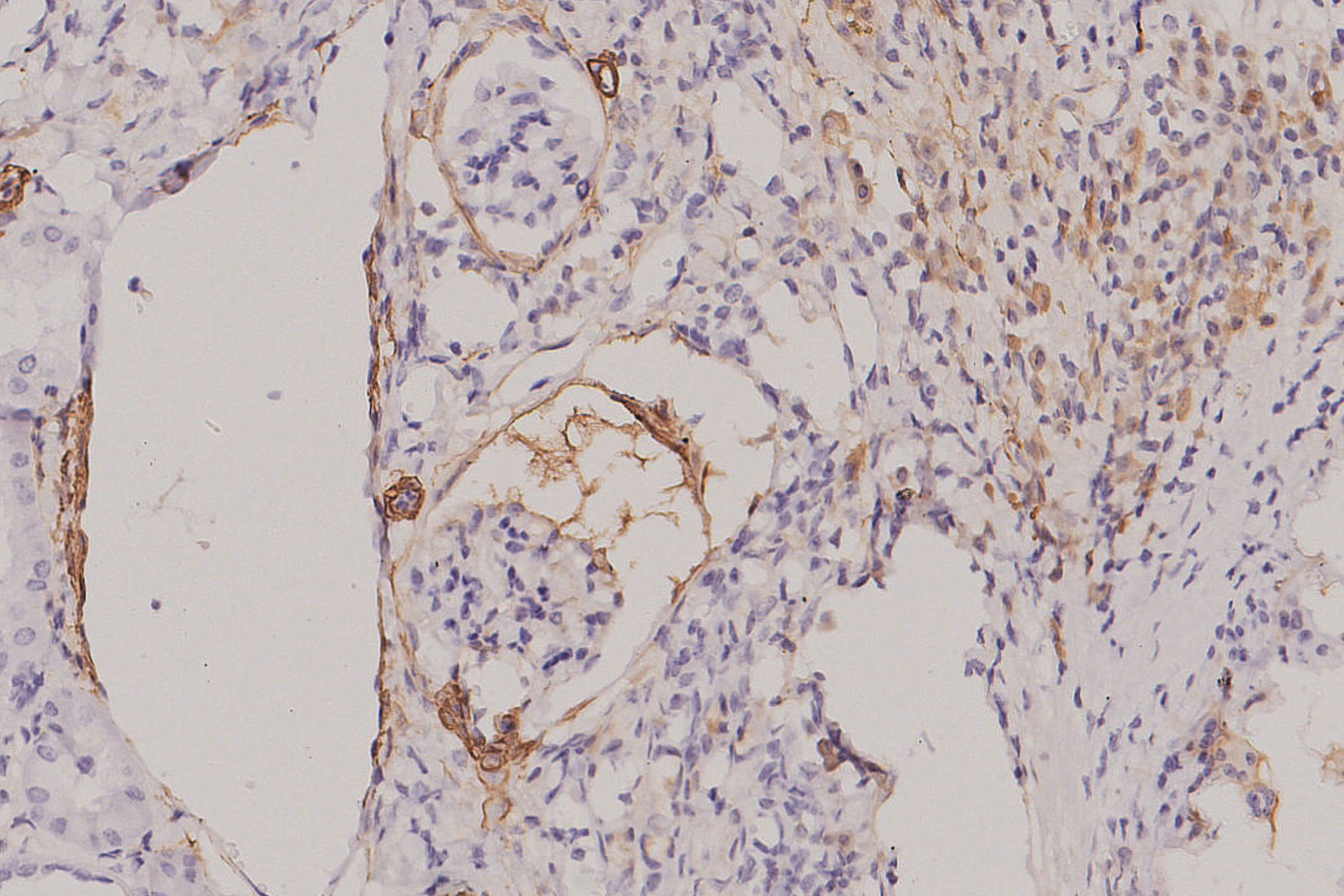

Supplement: Supplementary file 3 [file Data_Sheet_3.zip › NPD/NPD 4.jpg]

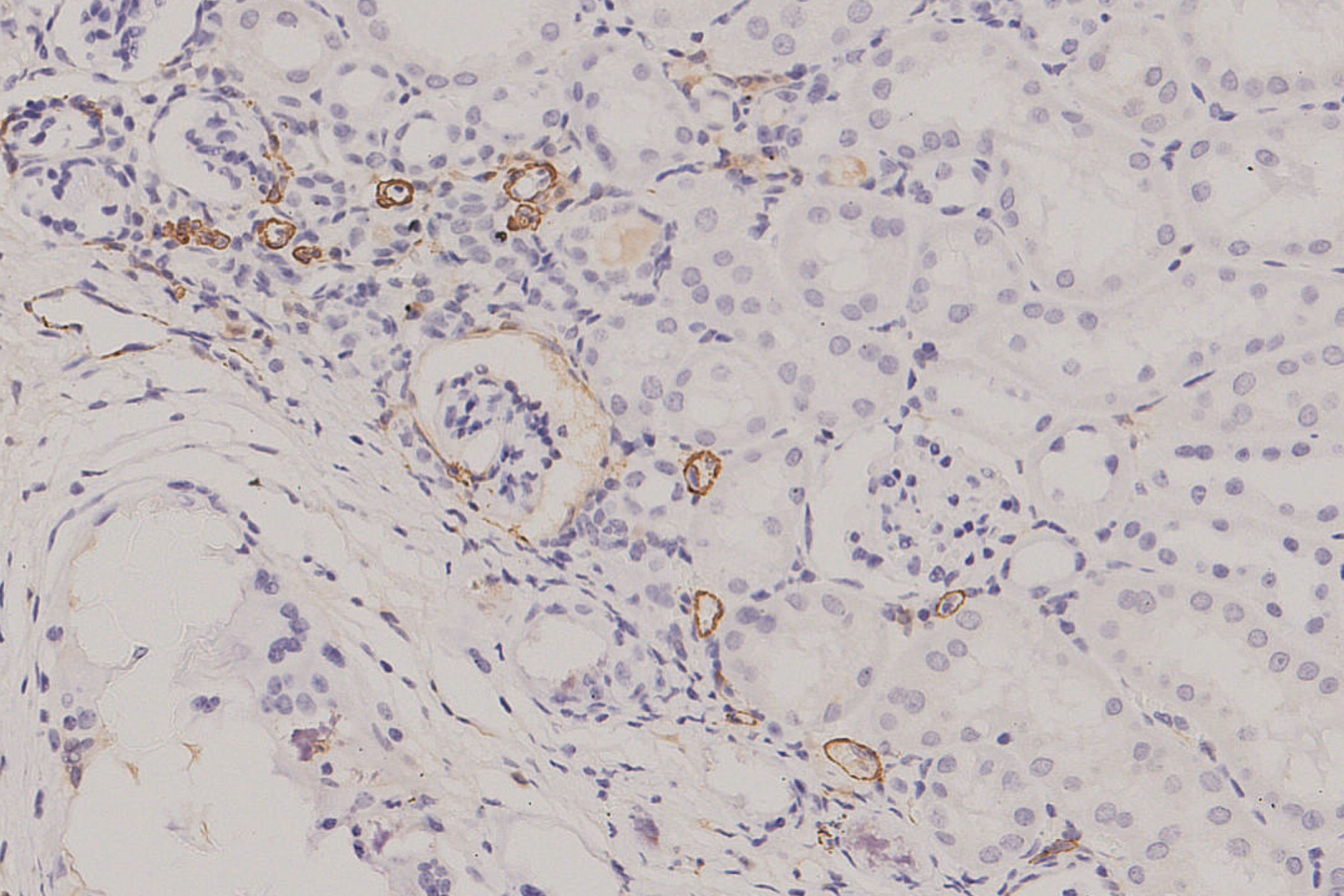

Supplement: Supplementary file 3 [file Data_Sheet_3.zip › NPD/NPD 5.jpg]

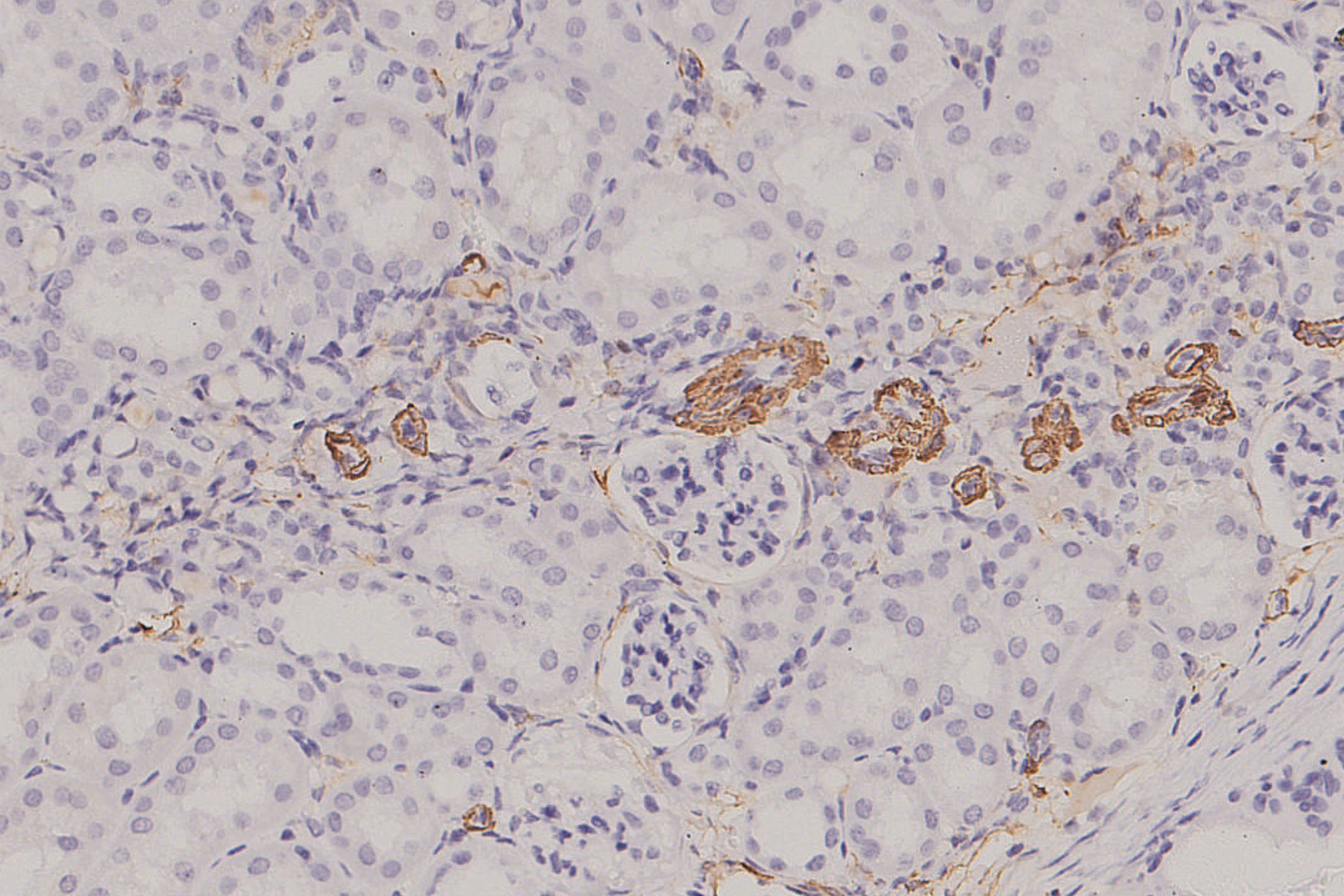

Supplement: Supplementary file 3 [file Data_Sheet_3.zip › NPD/NPD 6.jpg]

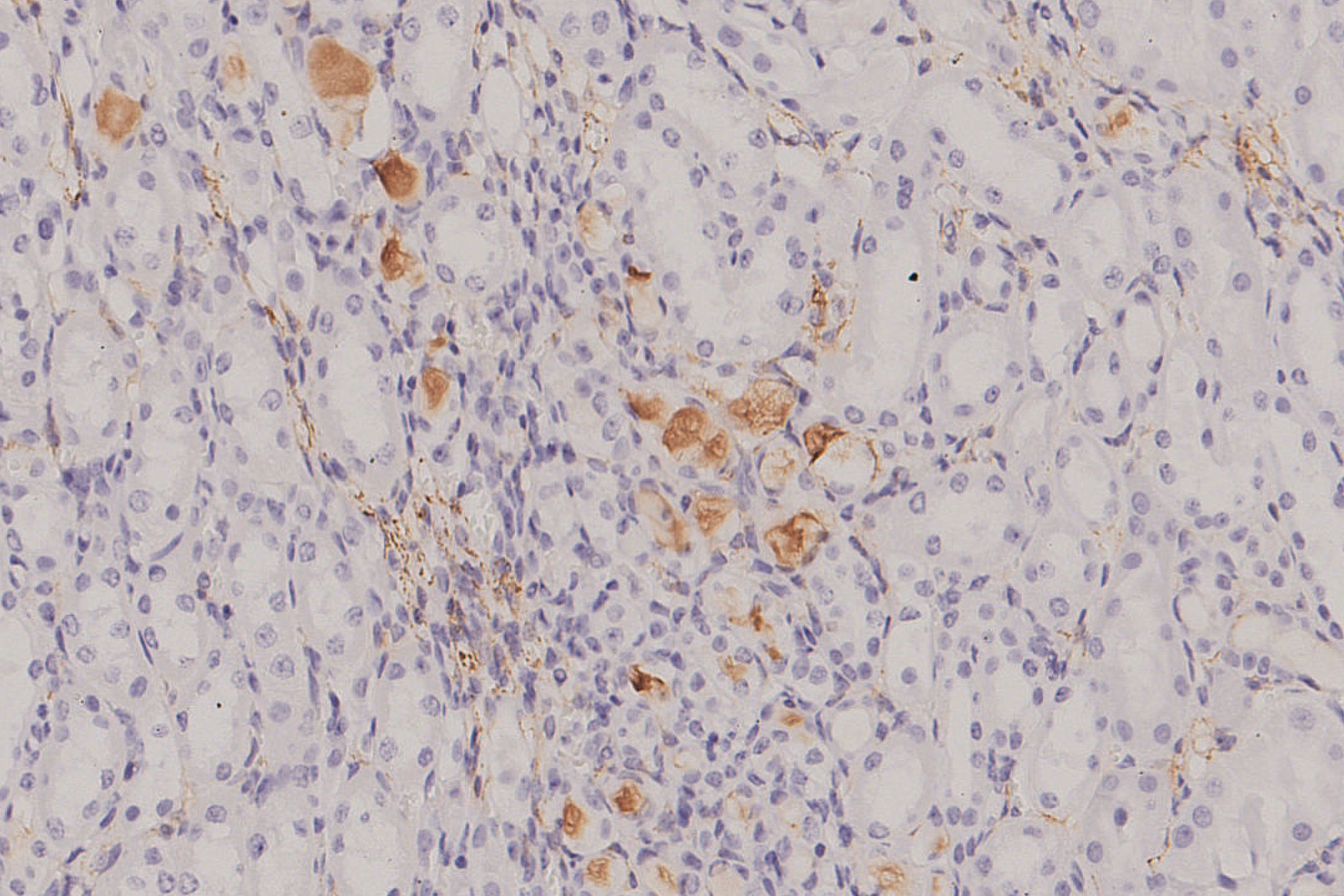

Supplement: Supplementary file 3 [file Data_Sheet_3.zip › NPD/NPD 7.jpg]

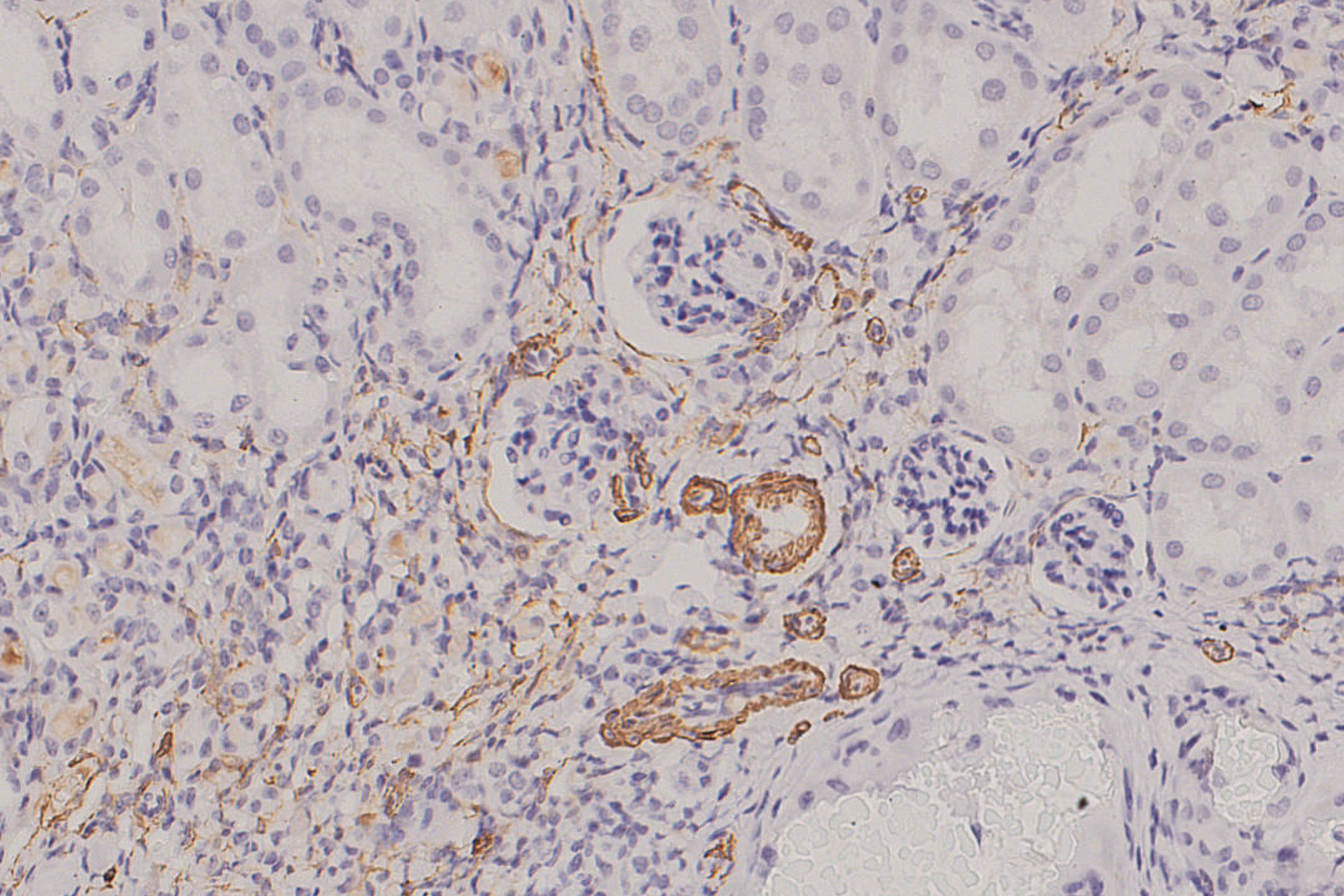

Supplement: Supplementary file 3 [file Data_Sheet_3.zip › NPD/NPD 8.jpg]

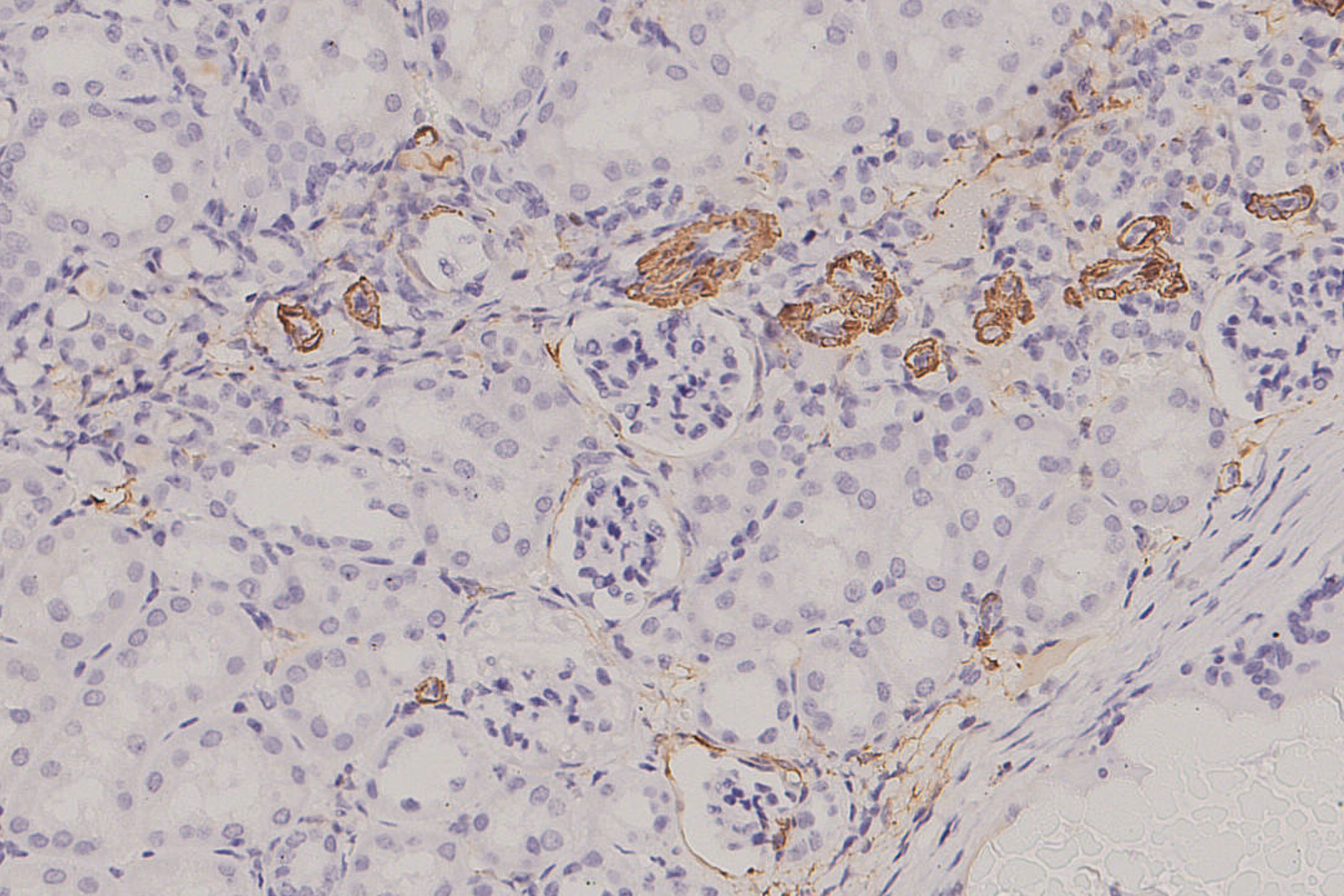

Supplement: Supplementary file 3 [file Data_Sheet_3.zip › NPD/NPD 9.jpg]

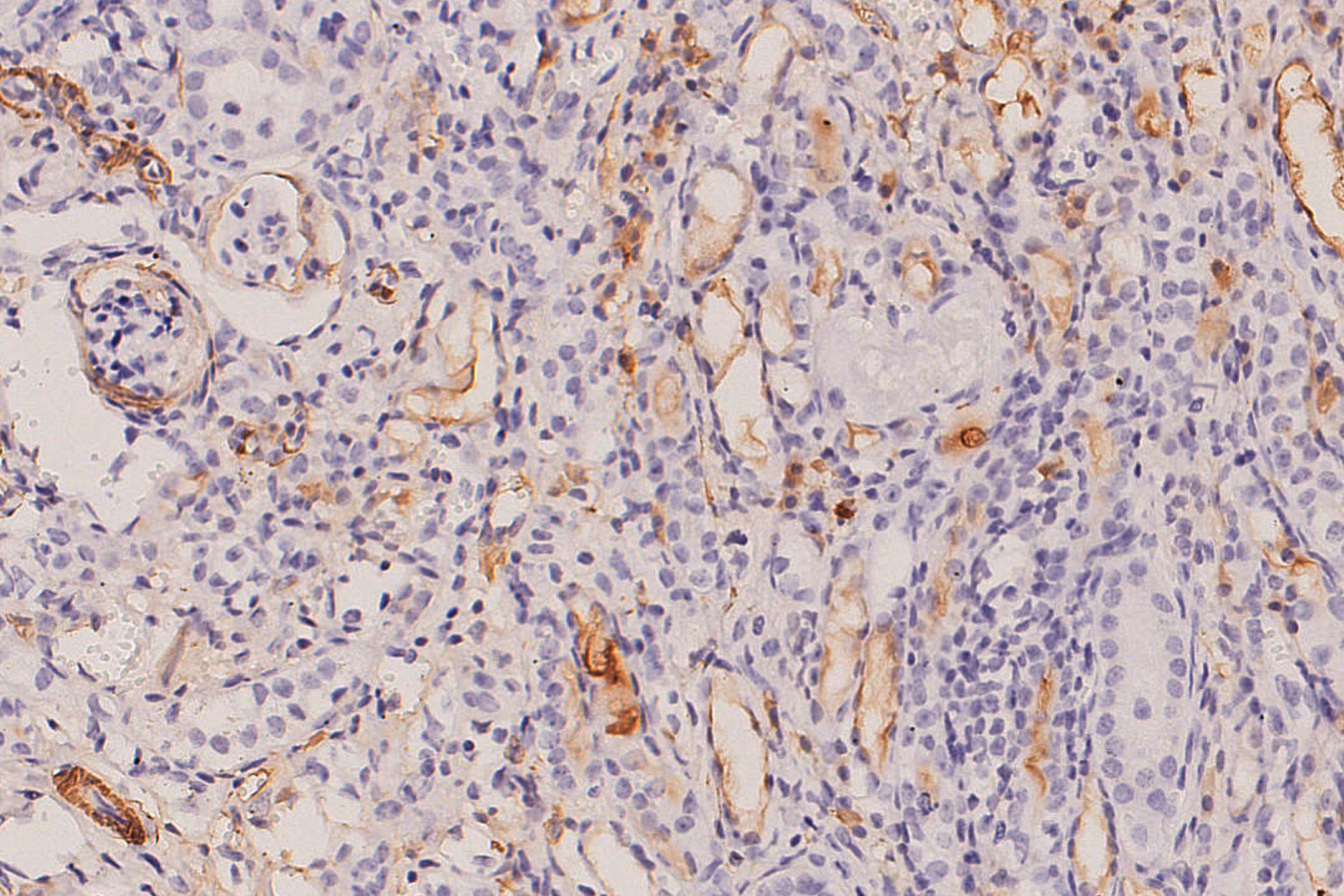

Supplement: Supplementary file 3 [file Data_Sheet_3.zip › NPD/NPD1.jpg]

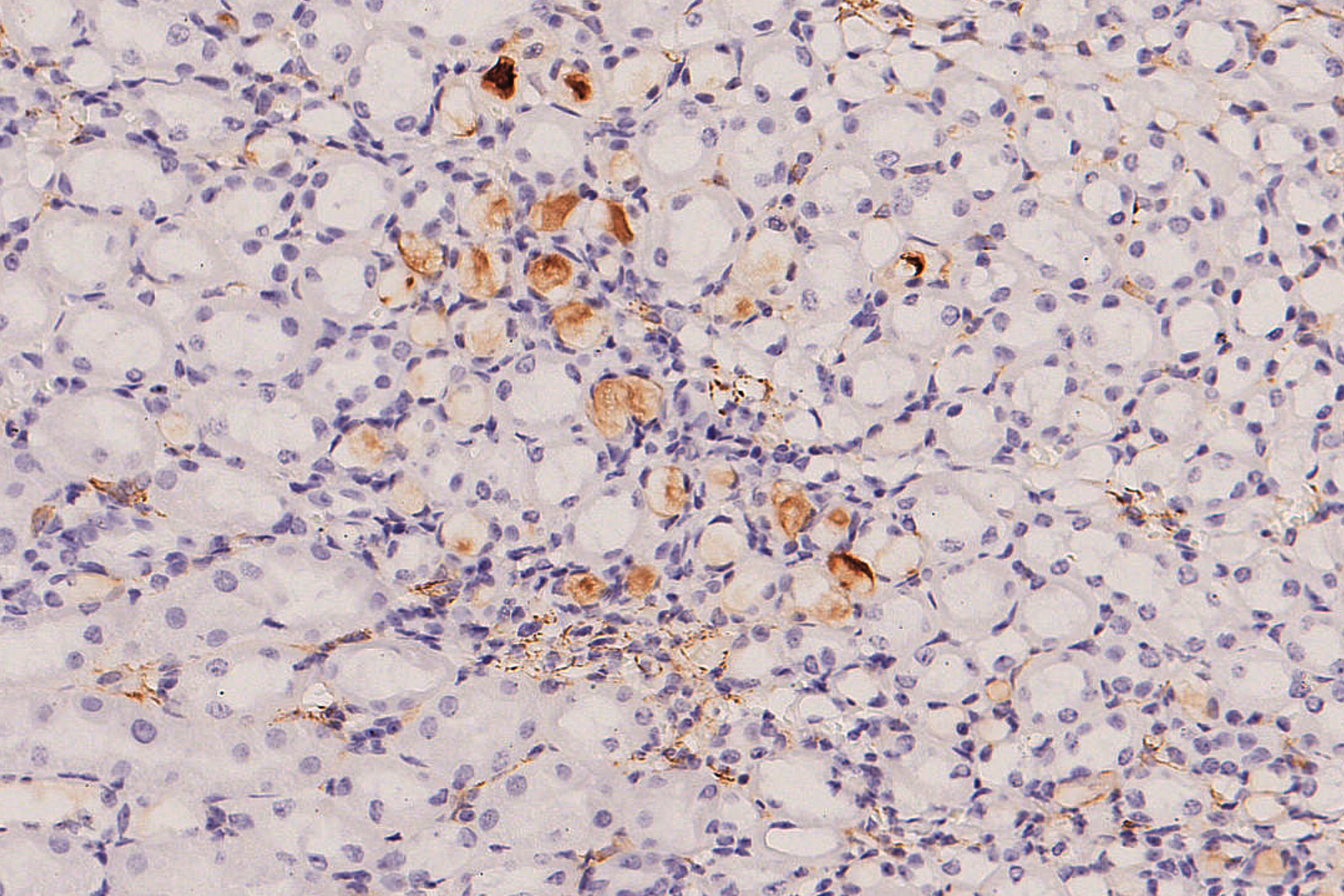

Supplement: Supplementary file 3 [file Data_Sheet_3.zip › NPD/NPD2.jpg]

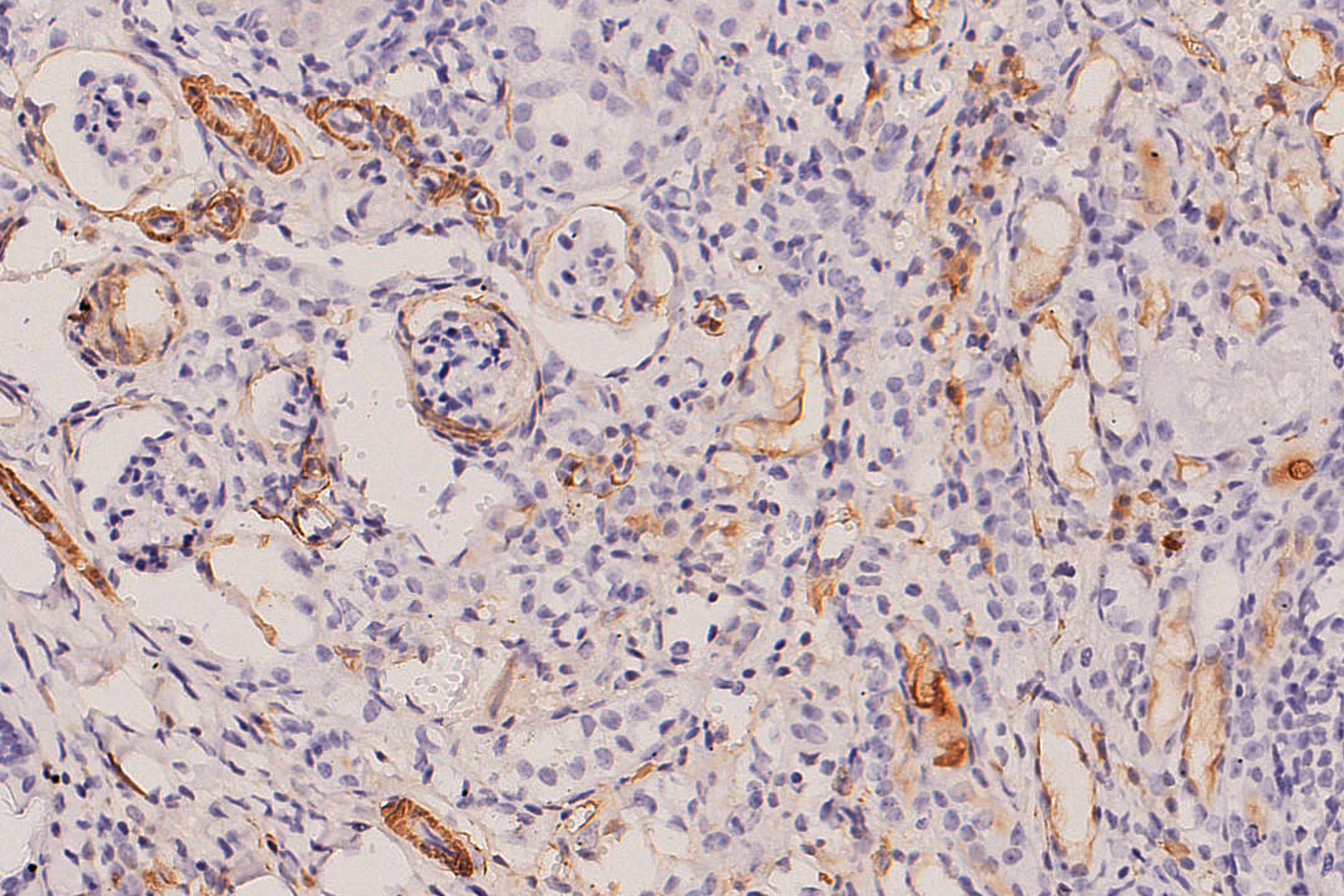

Supplement: Supplementary file 3 [file Data_Sheet_3.zip › NPD/NPD3.jpg]

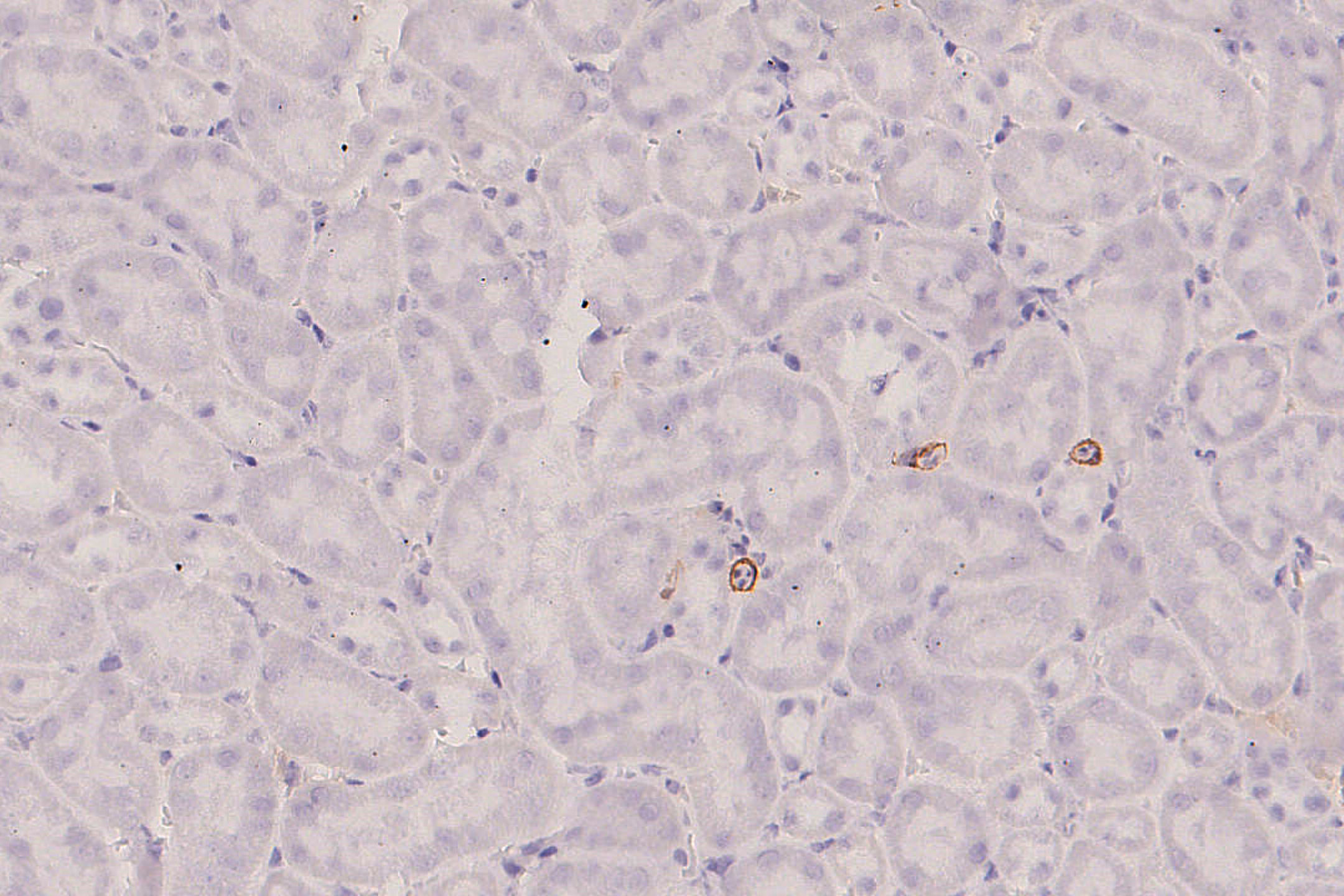

Supplement: Supplementary file 3 [file Data_Sheet_3.zip › Sham/Sham 2.jpg]

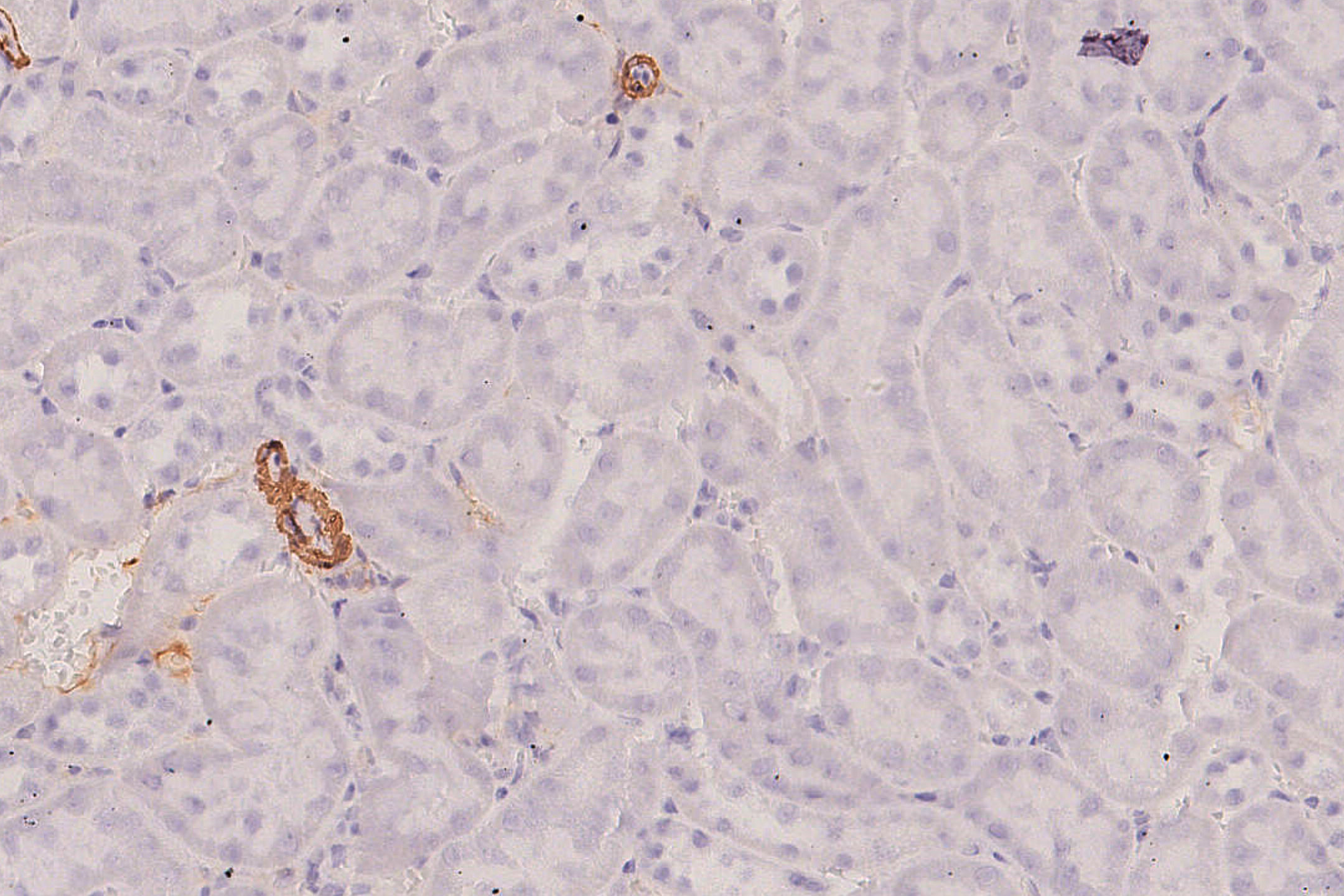

Supplement: Supplementary file 3 [file Data_Sheet_3.zip › Sham/Sham 3.jpg]

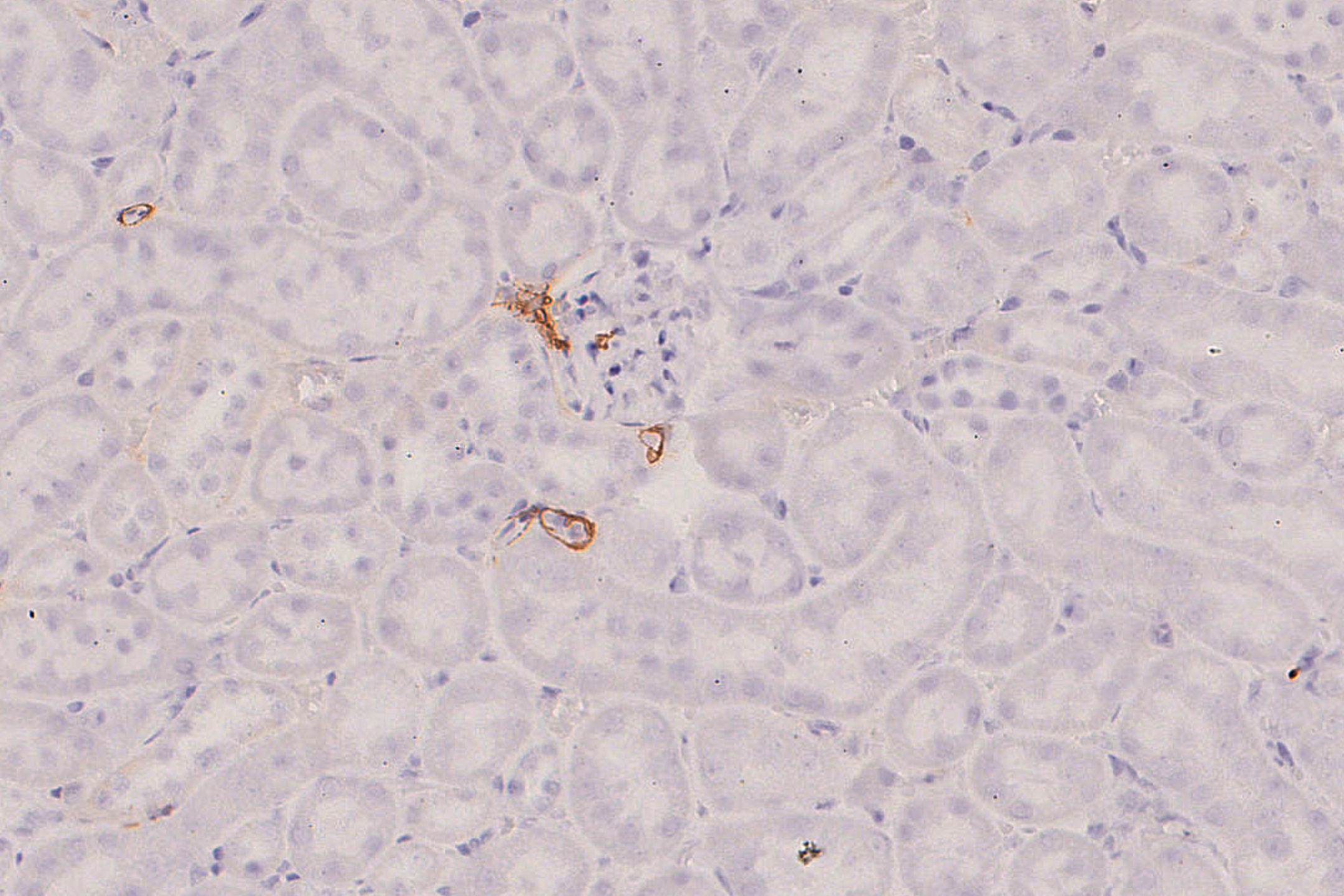

Supplement: Supplementary file 3 [file Data_Sheet_3.zip › Sham/Sham 4.jpg]

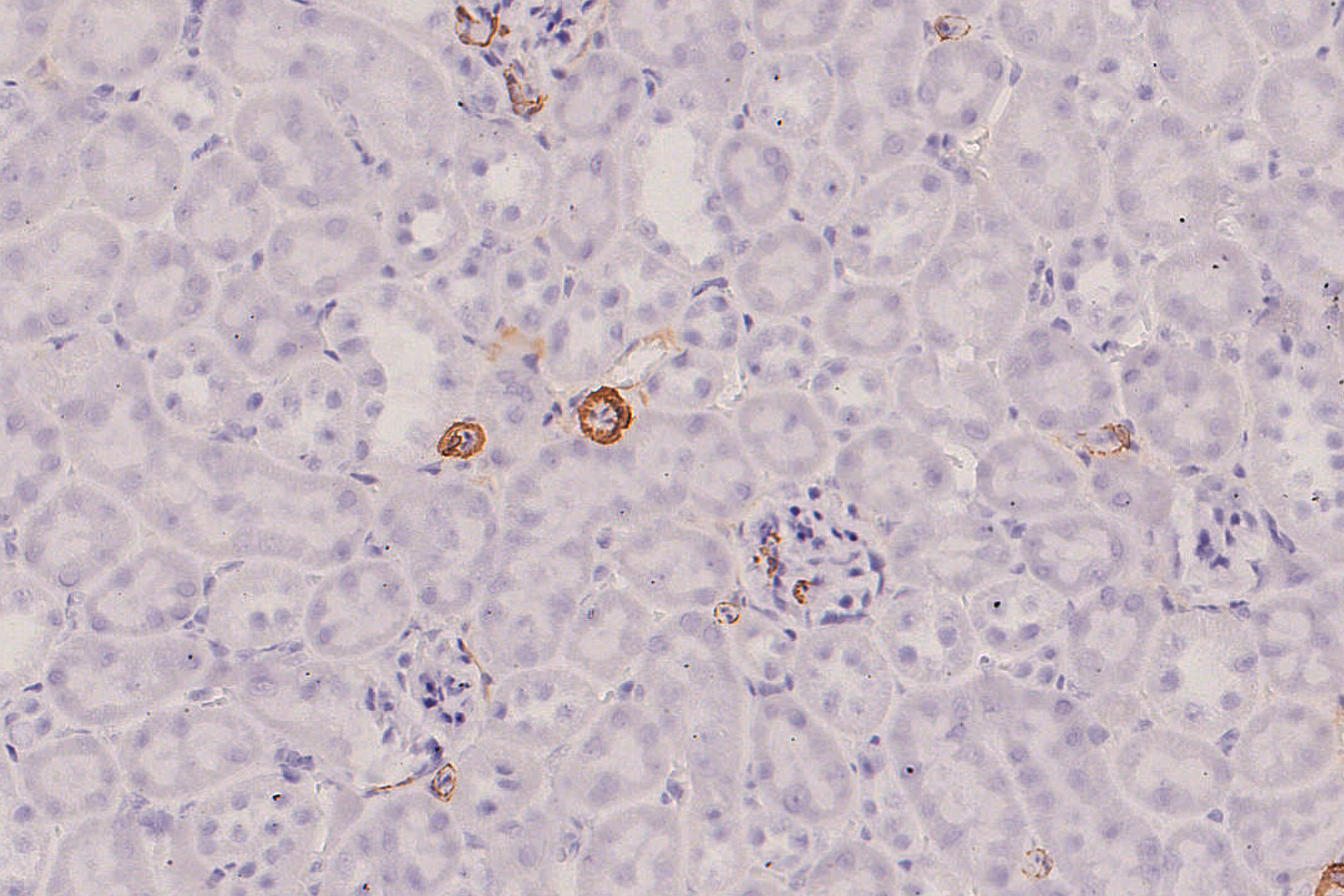

Supplement: Supplementary file 3 [file Data_Sheet_3.zip › Sham/Sham 5.jpg]

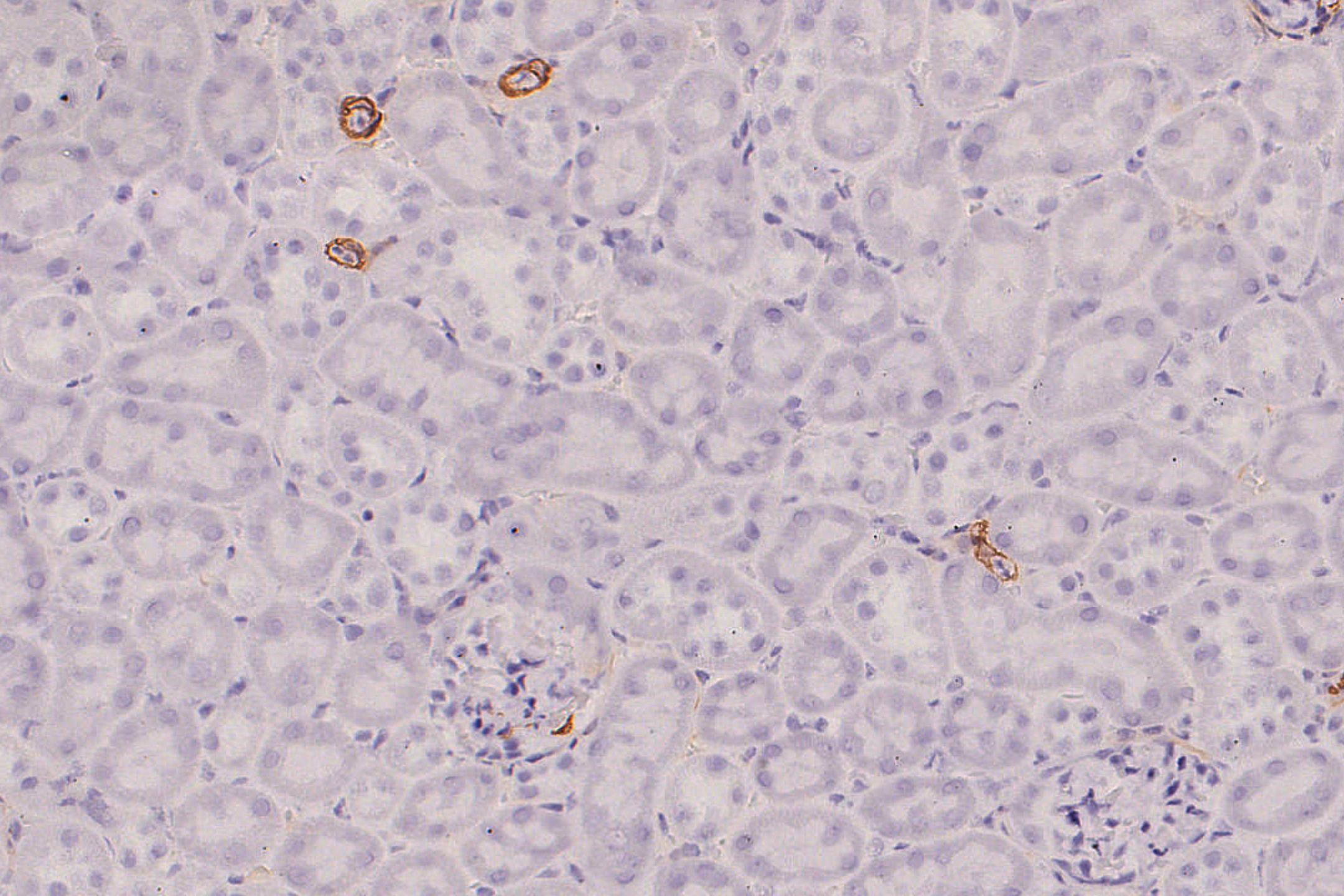

Supplement: Supplementary file 3 [file Data_Sheet_3.zip › Sham/Sham 6.jpg]

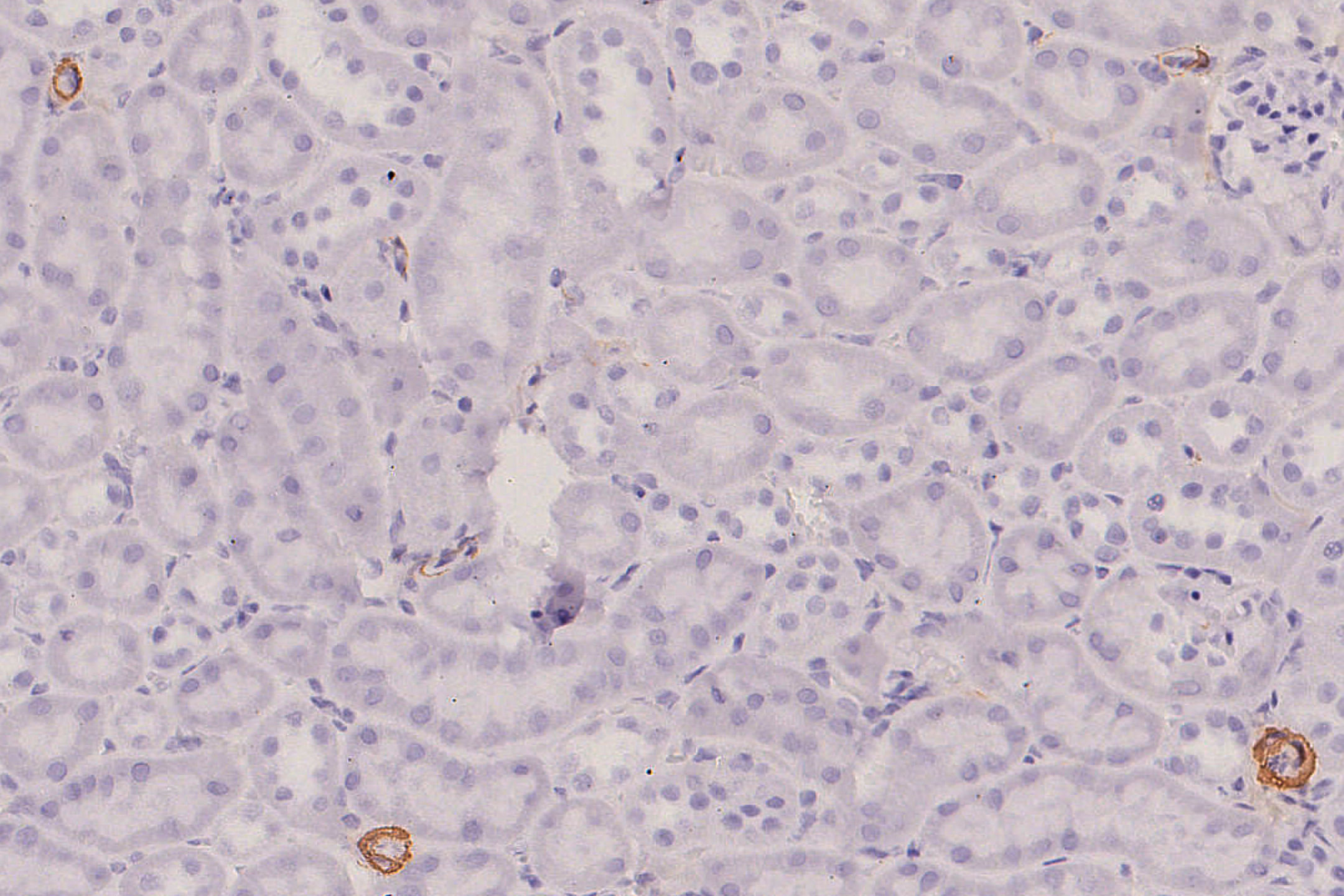

Supplement: Supplementary file 3 [file Data_Sheet_3.zip › Sham/Sham 7.jpg]

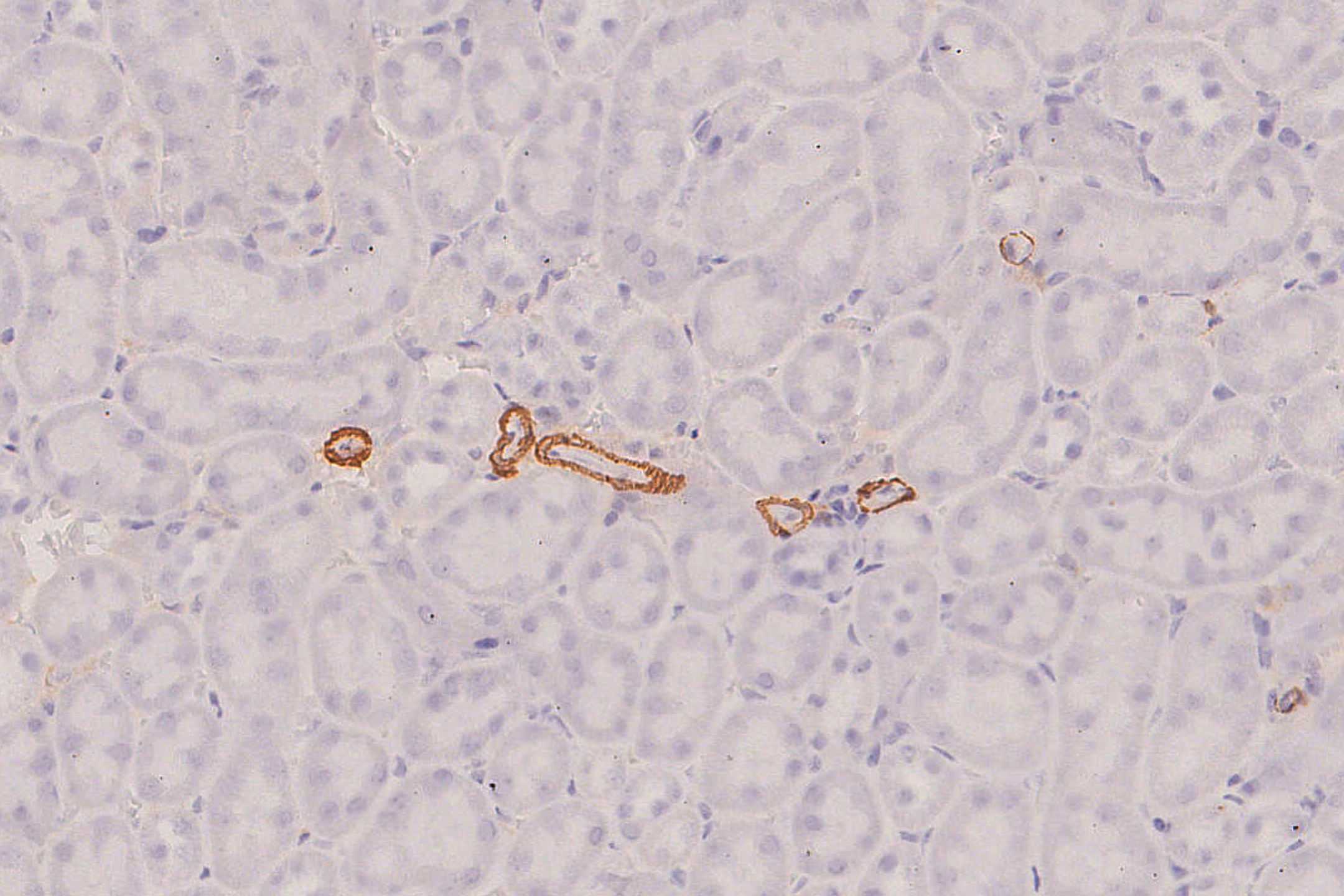

Supplement: Supplementary file 3 [file Data_Sheet_3.zip › Sham/Sham 8.jpg]

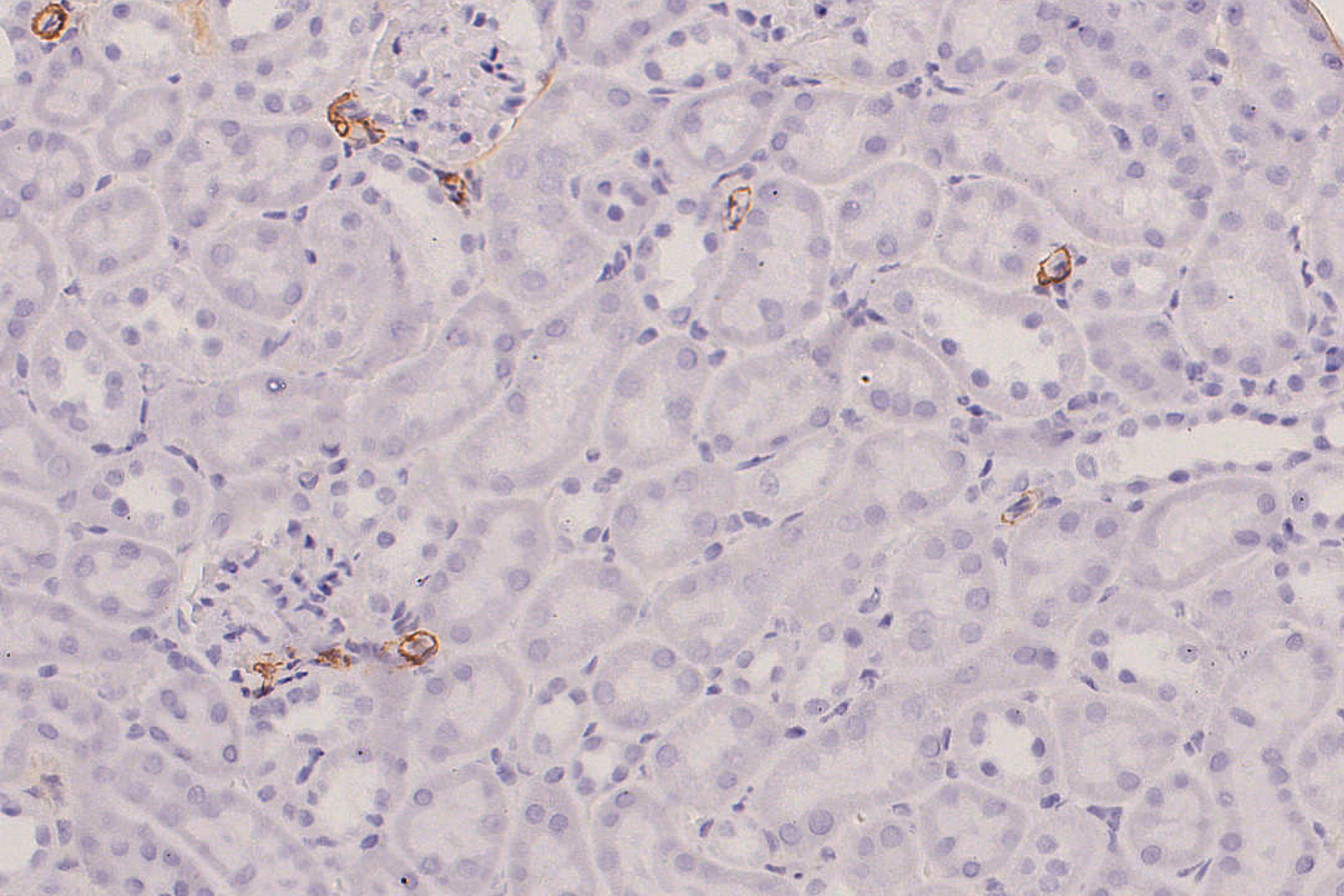

Supplement: Supplementary file 3 [file Data_Sheet_3.zip › Sham/Sham 9.jpg]

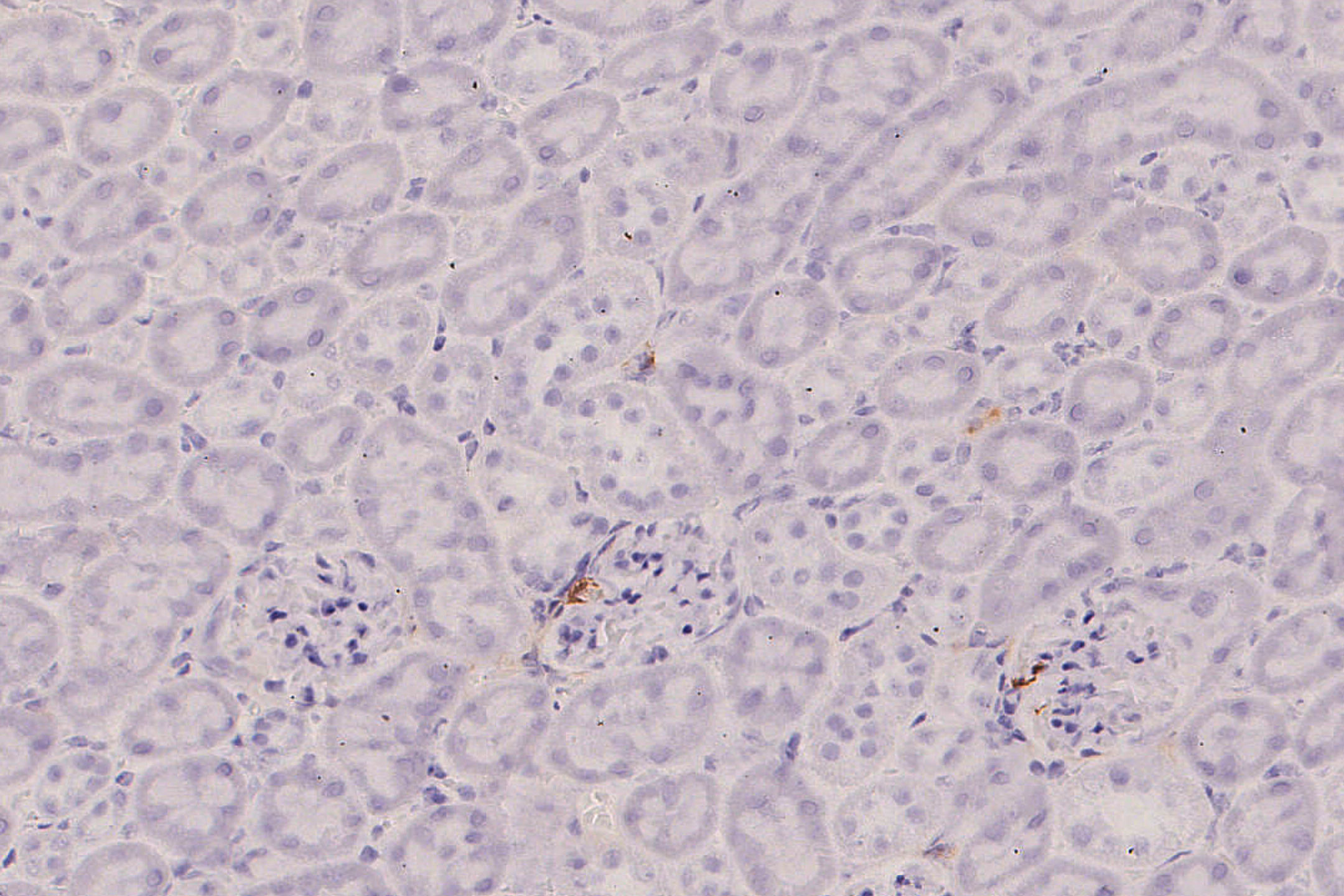

Supplement: Supplementary file 3 [file Data_Sheet_3.zip › Sham/Sham1.jpg]
